# Supplementary material for: Interferon stimulated immune profile changes in a humanized mouse model of HBV infection
Source: Nat Commun. 2023 Nov 15;14:7393. doi: 10.1038/s41467-023-43078-5 (PMC10652013; doi:10.1038/s41467-023-43078-5)
Supplement: Supplementary file 1 — Supplementary Information [file 41467_2023_43078_MOESM1_ESM.pdf]

SUPPLEMENTARY INFORMATION OF

Title： Interferon stimulated immune profile changes in a humanized mouse model of HBV infection

Author list: Yaping Wang[1]†, Liliangzi Guo[1]†, Jingrong Shi[1]†, Jingyun Li[2]†, Yanling Wen[3]†, Guoming Gu[4]†, Jianping Cui[1], Chengqian Feng[1], Mengling Jiang[1], Qinghong Fan[1], Jingyan Tang[1], Sisi Chen[1], Jun Zhang[1], Xiaowen Zheng[1], Meifang Pan[1], Xinnian Li[5,] Yanxia Sun[6], Zheng Zhang[3], Xian Li[4], Fengyu Hu[1], Liguo Zhang[2], Xiaoping Tang[1]*, Feng Li[1]*

Address:

[1]Institute of infectious Diseases, Guangzhou Eighth People's Hospital, Guangzhou Medical University, Guangzhou, China. 8 Huaying Road, Baiyun District, Guangzhou, Guangdong Province, China

[2]CAS Key Laboratory of Infection and Immunity, Institute of Biophysics, Chinese Academy of Sciences, Beijing, China

[3]Institute for Hepatology, National Clinical Research Center for Infectious Disease, Shenzhen Third People's Hospital; The Second Affiliated Hospital, School of Medicine, Southern University of Science and Technology, Shenzhen, China

[4]Guangzhou XY Biotechnology Co., LTD Room 2048, Building 1, NO.6, Nanjiang Second Road, Pearl River Street, Nansha District, Guangzhou, China

[5]Guangzhou Forevergen Medical Laboratory Room 802, NO.8, Luoxuan 3rd Road, Haizhu, Guangzhou，Guangdong，China

[6]Cytek (Shanghai) Biosciences Co, Ltd, Guangzhou, China

Correspondence:

Feng Li, E-mail: gz8h_lifeng@126.com;

Xiaoping Tang, E-mail: tangxp@gzhmu.edu.cn

Material and methods

Mouse genotyping by Polymerase Chain Reaction (PCR) and quantitative reverse transcription PCR

Approximately 0.4-0.6cm of tail tissue was cut and ground into fine powder in liquid nitrogen. DNA was extracted from the tail tissue using a genomic DNA extraction kit (Tiangen, China). PCR primers were ordered from Sangon Biotech (Shanghai, China). The PCR reaction system (Yisheng, China) consisted of Premix Taq 25 μL, forward and reverse primers 2 μL, DNA template (diluted template to 10ng/ul) 10 μL, and H2O up to 50 μL. Agarose gel (1%, g/volume, in 0.5 X TAE buffer) (Biowest, Spain) was prepared, including a nucleic acid dye GelRed (Yisheng, China). Then, 10 μL PCR products were loaded for analysis at 80 V electrophoresis voltage for 30 min, and gel images were taken using a gel imager (BioRad, USA). The amplified PCR product was sequenced by Sangon Biotech (Shanghai, China). The sequence alignment is provided in the supplementary information Appendix 2.

Western blot analysis

Approximately 200 mg of splenic tissue was cut into small chunks. The collected tissues were transferred to a 2 ml EP tube and 1 ml of cold RIPA buffer (including PMSF) was added. The tissue was ground twice in a tissue grinder at 60 Hz for 120 seconds. After allowing the tube to ice crack for 30

minutes, it was centrifuged at 15294g at 4°C for 10 minutes. The supernatant was carefully transferred to a new 1.5 ml EP tube, and an appropriate amount of 5X SDS loading buffer with 25 mM β-glycerol-phosphate was added. The mixture was then boiled for 10 minutes. The tissue lysates were separated by PAGE electrophoresis and transferred to a PVDF membrane with a pore size of 0.45 μm. The membrane was blocked with 5% skim milk in TBST for 1 hour at room temperature. Subsequently, the membrane was incubated with primary antibodies overnight at 4°C. Following incubation, the membrane was washed five times with TBST for 5 minutes per wash. For the secondary antibody incubation, the membrane was incubated for 2 hours at room temperature. After washing again with TBST, the proteins were visualized using a Bio-Rad Imaging system. The primary antibodies used in this study were anti-ACTIN (mouse, RM2001, Beijing Ray antibody) and anti-ISG15 (mouse, A2416, ABclonal).

Isolation of blood PBMC

Blood samples were collected from 1 healthy donor (Female, 35 years old) and 3 huIFNAR mice (6-10 weeks of age). Peripheral blood mononuclear cells (PBMCs) were isolated using Ficoll-Paque Plus (GE Healthcare Life Science, UK) according to the manufacturer’s instructions. Spleen was obtained from mice after euthanasia. Place the spleen in a 50 ml centrifuge tube with PBS (Invitrogen), press it through a 70 μm cell strainer, and centrifuge for 5 min at

450g. For red cell lysis, resuspend the pellet of spleen or PBMCs in 5 ml of 1 X RBC lysis buffer (BioLegend, USA) for 5 min at room temperature. Stop lysis by addition of 10ml of PBS and immediately centrifuge at 450g for 5 min.

Enzyme-linked immuno-spot assay

Cellular immune responses were assessed by interferon-γ(IFN-γ) enzyme-linked immuno-spot (ELISpot) assay, as previously described(ref 8) . Briefly, splenocyte suspensions were prepared by passing spleens through 70 μM cell strainers, followed by treatment with BD Pharm Lyse™ lysing solution and resuspension in complete minimum essential medium (Sigma). Splenocytes were restimulated separately with the three gE peptide pools at a final concentration of 2μg/ml in IPVH-membrane 96-well plates (Biolegend) previously coated with 5μg/mL anti-mouse IFN-γ Antibody (Biolegend). Plates were incubated for 18–20 h at 37°C with 5% CO_2 in a humidified incubator. Spotforming cells (SFCs) were detected with biotinylated antimouse IFN-γ (1 mg/mL) followed by streptavidinconjugated alkaline phosphatase (1 mg/mL) and alkaline phosphate substrate kit (BioRad). Spots were counted using an AID ELISpot reader and software (Autoimmun 2M. ULASZEWSKA ET AL.Diagnostika) and responses to each peptide pool were summed for analysis. Background responses (from splenocytes cultured with media alone, without peptide stimulation) were subtracted from responses in stimulated wells.

Cytokine analysis

Simultaneous quantification of cytokines in mouse sera was performed using LEGENDplex Mouse Inflammation Panel (13-plex) with V-bottom Plate (BioLegend Cat# 740446) according to manufacturer's instructions. In brief, samples were thawed completely, mixed, and centrifuged to remove particulates prior to use. To achieve measurement accuracy, serum samples were diluted 2-fold with Assay Buffer, and standards were mixed with Matrix solution (Biolegend) to account for additional components in the serum samples. Standards and samples were plated with capture beads for tumor necrosis factor-alpha (TNFα), IFN-γ, interleukin-1alpha (IL-1α), IL-1β, IL-6, IL-10, IL-17A, IL-12p70, granulocyte-macrophage colony-stimulating factor (GM-CSF), IL-23, IFNβ, monocyte chemoattractant protein-1 (MCP-1), IL-27 and incubated for two h at room temperature on a plate shake 68g . After washing the plate with Wash Buffer, Detection Antibodies were added to each well. The plate was incubated on a shaker for 1h at room temperature. Finally, without washing, streptavidin R-phycoerythrin (SA-PE) was added and incubated for 30 min. Samples were acquired on CytoFLEX flow cytometer (Beckman Coulter Life Sciences). Analysis was performed on a BD FACSymphony machine and using the LEGENDplex data analysis software (BioLegend).

Supplemental Table 1. Primer list for PCR and qRT-PCR

Primer ID	Sequence(5'-3')
5’arm fwd	GGTGGCTACCGTAATGTCGGTA
5’arm rev	AGCCTCCAGATGCACTTCAGGG
3’arm fwd	TGGACAACTGGATCAAGCTGTC
3’arm rev	GGCCCAGACTGTCAACATTACTCT
Full fwd	CTTAGAGGACAGATGTGACACGC
Full rew	GACATCAGTGTGCTGCTCCACAC
Hu IFNAR2 fwd	CACAAGCCTGAGATCAAG
Hu IFNAR2 rev	TAGACAGAGACACAGTAGTT
mMx2 fwd	GTGGCAGAGGGAGAATGTCG
mMx2 rev	TAAAACAGCATAACCTTTTGCGA
mMx1 fwd	AAGATGGTCCAAACTGCCTTCG
mMx1 rev	GCCTTGGTCTTCTCTTTCTCAGC
mIsg15 fwd	AACTGCAGCGAGCCTCTGA
mIsg15 rev	CACCTTCTTCTTAAGCGTGTCTACAG
mGAPDH fwd	AACTTTGGCATTGTGGAAGGGCTCA
mGAPDH rev	TTGGCAGCACCAGTGGATGCAGGGA
Oas1a fwd	ATTACCTCCTTCCCGACACC
Oas1a rev	CAAACTCCACCTCCTGATGC
Gbp2 fwd	CTGCACTATGTGACGGAGCTA
Gbp2 rev	CGGAATCGTCTACCCCACTC
Psrc1 fwd	GCATAAAGAAGGAATCACCCACT
Psrc1 rev	CACCGAACCCAGTTTTCCG
Sectm1b fwd	AGCCTCCCTGAATGCCTATAA
Sectm1b rev	ACGTCTCTGAGATTGTTGGAGAT
Ifi44 fwd	AACTGACTGCTCGCAATAATGT

Ifi44 rev	GTAACACAGCAATGCCTCTTGT
Apol6 fwd	GCTTTGGTGCAAACTCCAACC
Apol6 rev	GCGTTCTAATGCGCTTCTTCT
Usp18 fwd	TTGGGCTCCTGAGGAAACC
Usp18 rev	CGATGTTGTGTAAACCAACCAGA
Ifit1 fwd	CTGAGATGTCACTTCACATGGAA
Ifit1 rev	GTGCATCCCCAATGGGTTCT
Alox15 fwd	CAGGGATCGGAGTACACGTT
Alox15 rev	GATTGTGCCATCCTTCCAGT
Mxd3 fwd	GAGGCAGAGCACGGTTATG
Mxd3 rev	TGTAGTGTATCGGGTACAGTCAA
Trim10 fwd	AGCTGCCCTCTCTGCAAAG
Trim10 rev	CCTCCTCGCAAAAGAAGTAGATT
Tnk1 fwd	AAGGTTGTAGAGGTAGAG
Tnk1 rev	GTTACTAAGGTGGAAGAG
Cxcl10 fwd	CCAAGTGCTGCCGTCATTTTC
Cxcl10 rev	GGCTCGCAGGGATGATTTCAA
Gbp10 fwd	CATAACATGATGCTGAAG
Gbp10 rev	TTCTTATCCTTGATGACAT
Sectm1a fwd	CAGTGCCCGCTATCCCTAC
Sectm1a rev	TCGGTGAAGGTGTTAGAGATGT
Rsad2 fwd	TGCTGGCTGAGAATAGCATTAGG
Rsad2 rev	GCTGAGTGCTGTTCCCATCT
Cngb1 fwd	CAGAGGAGGAACACTACTGCG
Cngb1 rev	AAGTAATCCATGAGGAGCCAGA
Ifnar2 fwd	CTTCGTGTTTGGTAGTGATGGT
Ifnar2 rev	GGGGATGATTTCCAGCCGA
Stmn2 fwd	GAGGAGAACAACAACTTC
Stmn2 rev	CAATGATAGCAGCTAGATTA

Ifitm6 fwd	GAGGGATCCTGACTCAGC
Ifitm6 rev	AGCATGGGATTGGGCCCCAGTC
Oas3 fwd	TCTGGGGTCGCTAAACATCAC
Oas3 rev	GATGACGAGTTCGACATCGGT
Sifn4 fwd	GGCTCCCTGCGTAAAGGAAC
Sifn4 rev	GGGTAACATATTTTCGCGCTTGA
Itga2 fwd	TGTCTGGCGTATAATGTTGGC
Itga2 rev	CTTGTGGGTTCGTAAGCTGCT
Upk3b fwd	AGACCTGATTGCCTACGTGC
Upk3b rev	GGTGTCCTTAGTTGAGACATGCT
Rasal1 fwd	GCCAAGGACGTGTCTGGAAG
Rasal1 rev	TGAACGGTGTACTCCTCCCC
Cd209a fwd	CTCAACTTGTGGTCATCA
Cd209a rev	TGTAGACTCCTTGCTCAT
Nps fwd	AACTCAGCTTCGTCTTAG
Nps rev	AGAAAGTAATCAGGCTTCC
Pou4f3 fwd	ATGCGCCGAGTTTGTCTCC
Pou4f3 rev	GGGCTTGAACGGATGGTTCT
Dsp fwd	GGATTCTTCTAGGGAGACTCAGT
Dsp rev	TCCACTCGTATTCCGTCTGGG
Hoxb6 fwd	GCTCTACTCGTCTGGCTATGC
Hoxb6 rev	GTGGGTAATAGGAGGACGCC
Irg1 fwd	GACAGGCACAGAAGTG
Irg1 rev	AGGGTGCCATGTGTCATC
Fabp1 fwd	ATGAACTTCTCCGGCAAGTACC
Fabp1 rev	CTGACACCCCCTTGATGTCC
Cd79a fwd	TGCTGCTATTCAGGAAAC
Cd79a rev	TCATACATAGAACAGTCATCAA
Mmp9 fwd	TCTACAGAGTCTTTGAGT

Mmp9 rev	TCTATGATTCAGGAACTTC
Ido1 fwd	CAAGACACAATGAAGACT
Ido1 rev	CATAATGTAAGTATCTACTATTGC
Fcgr4 fwd	ATGTGGCAGCTACTACTACCA
Fcgr4 rev	ACCCACTTGGGGTCTAGGTTC
Nlrc3 fwd	CAGATTGGTAACAAAGGAGCCA
Nlrc3 rev	CGTTCGGTTTATCTTCAGAGCA
Tnnt1 fwd	CCTGTGGTGCCTCCTTTGATT
Tnnt1 rev	TGCGGTCTTTTAGTGCAATGAG
Ifit3 fwd	GCAACAATGTATGACTTA
Ifit3 rev	TCTGACTTCCTTAAATCT
Fgf21 fwd	CTGCTGGGGGTCTACCAAG
Fgf21 rev	CTGCGCCTACCACTGTTCC
Ccl8 fwd	AGAGAATCAACAATATCCAG
Ccl8 rev	GATCTCCATGTACTCACT
Nupr1 fwd	CCCTTCCCAGCAACCTCTAAA
Nupr1 rev	TCTTGGTCCGACCTTTCCGA
Cd163 fwd	ATGGGTGGACACAGAATGGTT
Cd163 rev	CAGGAGCGTTAGTGACAGCAG
Alas2 fwd	TCAGAACAATATGACTGGAAG
Alas2 rev	GTCTTGAACACACGGTAG
HBV DNA fwd	GAGTGTGGATTCGCACTCC
HBV DNA rev	GAGGCGAGGGAGTTCTTCT
HBV pgRNA fwd	GAGTGTGGATTCGCACTCC
HBV pgRNA rev	GAGGCGAGGGAGTTCTTCT
HBV total mRNA fwd	TCACCAGCACCATGCAAC
HBV total mRNA rev	AAGCCACCCAAGGCACAG

Supplemental Table 2. Antibodies used for flow cytometry analysis.

Marker	Fluorochrome	Manufacturer	Dilution μl/10^6 cell /100 μl	Clone	Cataloge No.
NK1.1	APC	Biolegend	1.0	PK136	108710
B220	Percp-Cy5.5	Biolegend	0.5	RA3-6B2	103236
TCRγ/δ	BV421	Biolegend	2.5	GL3	118120
F4/80	PE-Dazzle594	Biolegend	3.0	BM8	123146
CD49b	PE/Cy7	Biolegend	0.4	HMα2	103518
CD8	BV570	Biolegend	0.5	53-6.7	100740
CD25	BV605	Biolegend	2.5	PC61	102036
CD19	Pacific Blue	Biolegend	0.5	6D5	115523
Ly6c	APC-Fire750	Biolegend	0.4	HK1.4	128046
CD3	Alexa Fluor 488	Biolegend	0.6	145-2C11	100321
PD1	PE	Biolegend	5.0	RMP1-14	114118
CD62L	BV785	Biolegend	0.5	MEL-14	104440
CXCR5	BV650	Biolegend	5.0	L138D7	145517
CD4	BV750	Biolegend	2.5	GK1.5	100467
CD11c	SB436	Thermo	2.5	N418	62-0114-82
MHCII	AF700	Biolegend	1.0	M5/114.15.2	107622
CD44	BV480	BD Biosciences	2.0	IM7	566200
CD11b	BV711	BD Biosciences	3.0	M1/70	563168
CD49a	Alexa Fluor 647	BD Biosciences	5.0	Ha31/8	562113
CD45	Alexa Fluor 532	Thermo	0.3	30-F11	58-0451-82
FVS575V	BV570	BD Biosciences	0.2	/	565694

Supplementary Fig 4b-related DNA gel: Raw DNA gel map No-cut

Details to see Supplementary Fig. 4b.

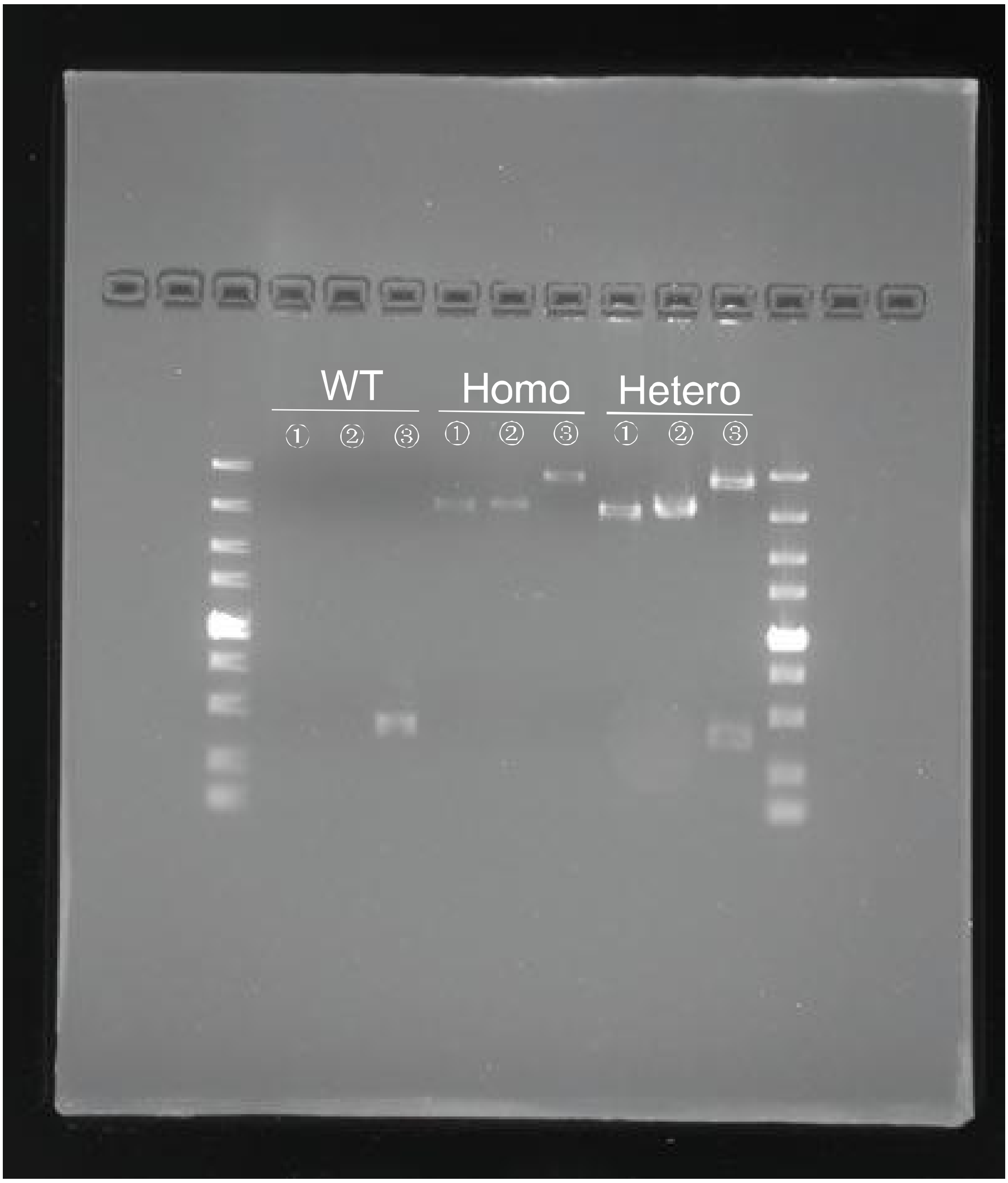

Supplementary Fig. 5a-related Raw picture of mouse ISG15 and mouse ACTIN

Details to see Supplementary Fig. 5a.

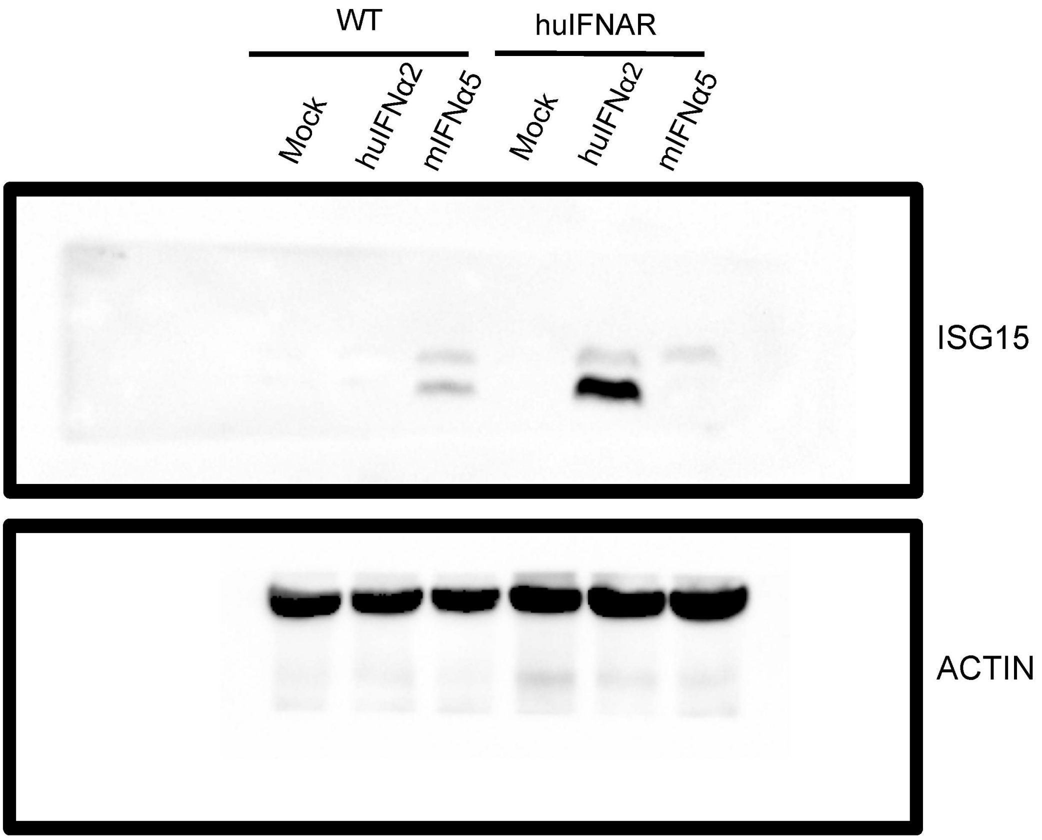

Supp. Figure 1

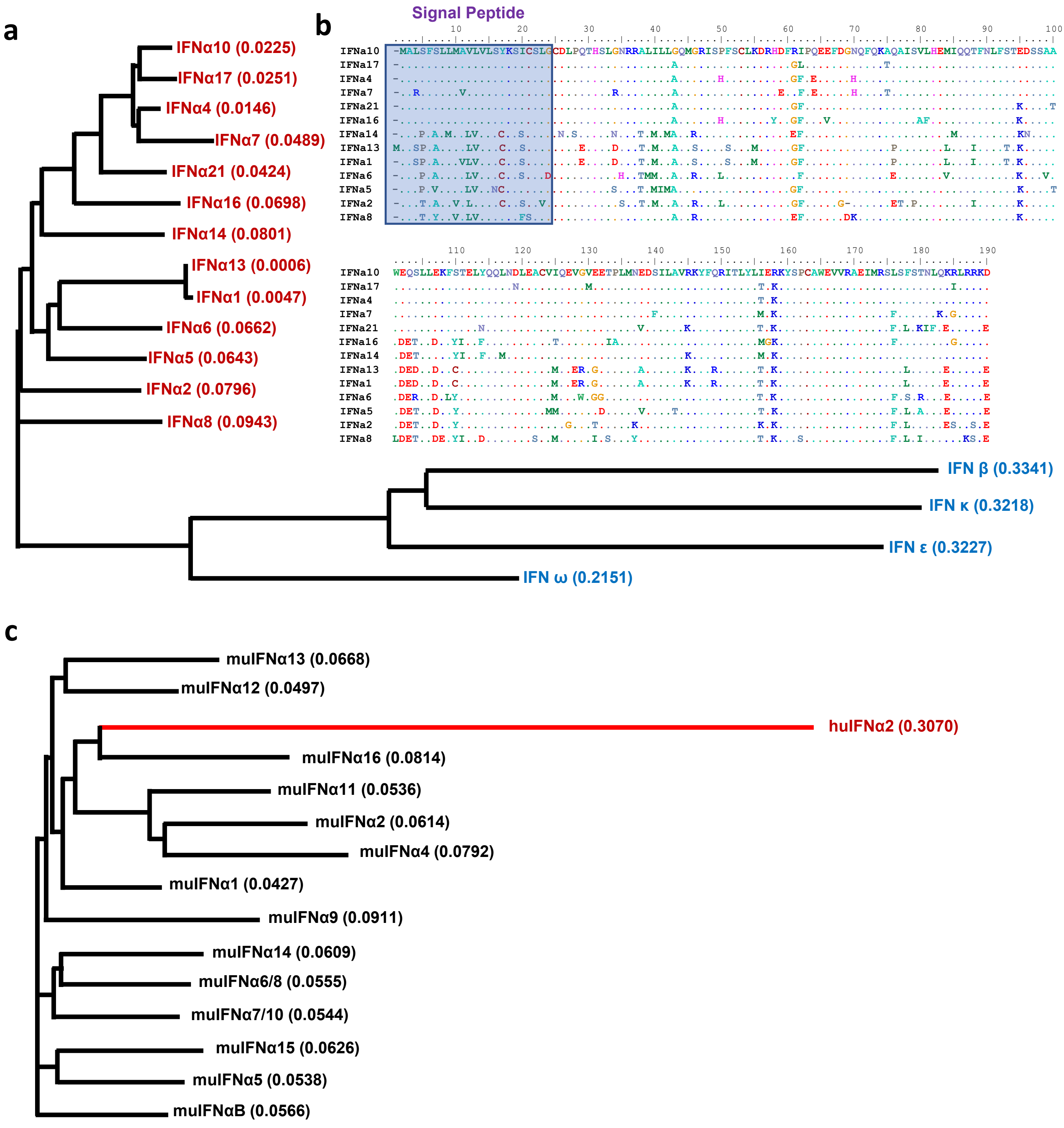

Supplementary Figure 1：Sequence alignment of Type I interferons. (**a**) Phylogenetic tree alignment of the type I interferon family. The phylogenetic tree was constructed using the neighbor-joining (NJ) method by MEGA through the Kimura two-parameter model with 1000 bootstrap replications. The branch lengths are shown in brackets. (**b**) Sequence alignment of 13 isotypes of interferon alpha, corresponding to the sequence in the (a) from top to bottom. Only varied amino acids are shown. (**c**) Phylogenetic tree alignment of human IFNα2 to all the mouse type I interferons.

Supp. Figure 2

a

IFNAR1 extracellular domain

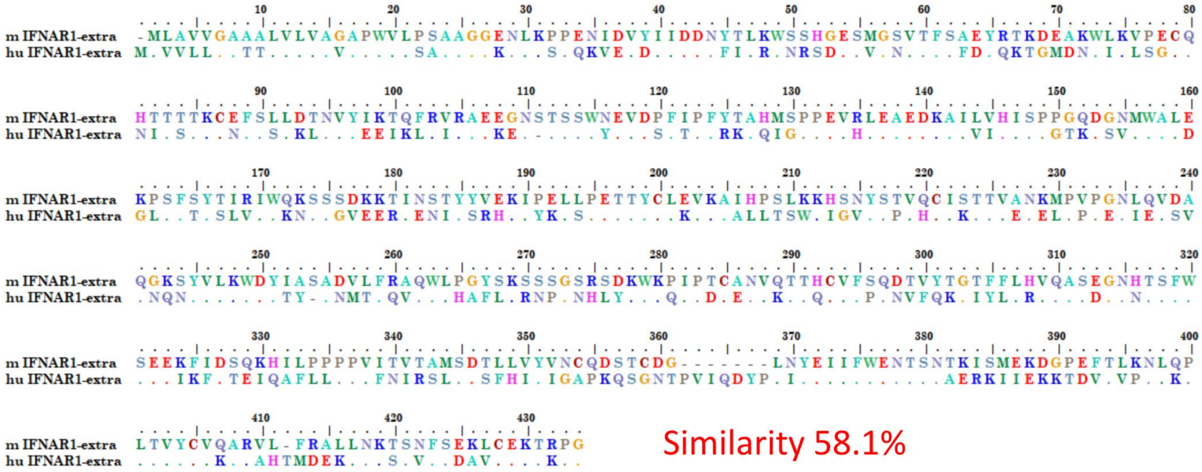

b

IFNAR2 extracellular domain

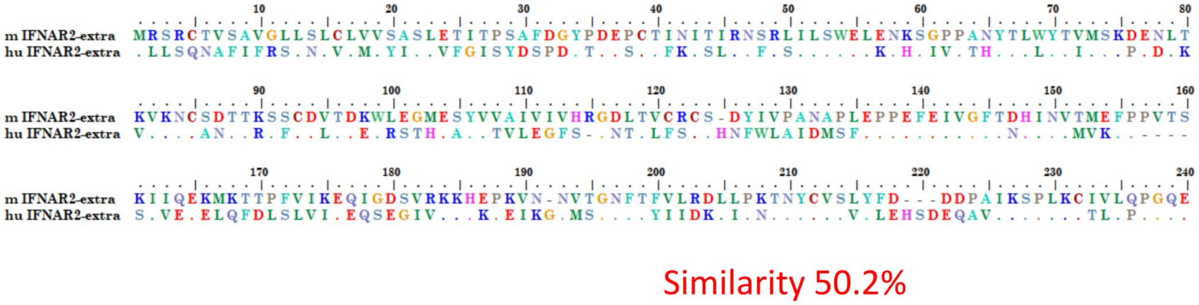

Supplementary Figure 2: Amino acid sequence alignment of the extracellular domains of type I interferons receptor between human and mouse. (**a**) Interferon receptor subunit 1 (IFNAR1) extracellular domain. (**b**) Interferon receptor subunit 2 (IFNAR2) extracellular domain. Similarity is indicated as percentage. m, mouse. hu human. Extra, extracellular domain.

Supp. Figure 3

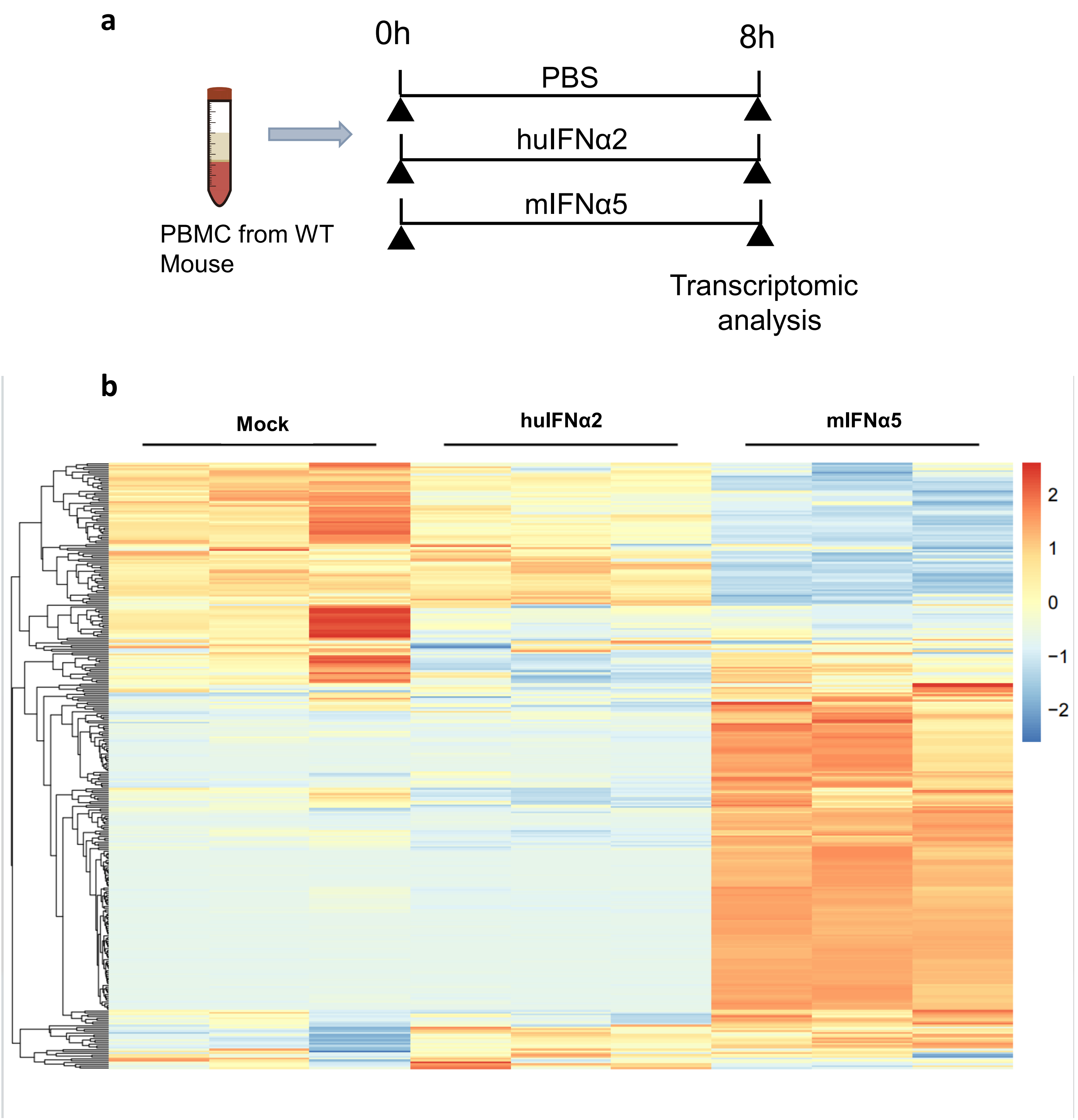

Supplementary Figure 3：Modulation of Gene Expression in Wildtype Mice Subject to Human and Mouse Interferons. (**a**) Schematic of the experimental strategy. PBMCS from wild-type mice were isolated and stimulated with human IFNα2 (huIFNα2, 800ng/ml) and mouse IFNα5 (mIFNα5, 275ng/ml) for 8 hours in vitro, respectively, for transcriptome sequencing. (**b**) The heatmap illustrates the differential expression of interferon-stimulated genes (ISGs) within peripheral blood mononuclear cells (PBMCs) derived from wild-type mice following an 8-hour exposure to huIFNα2 and mIFNα5.

Supp. Figure 4

a

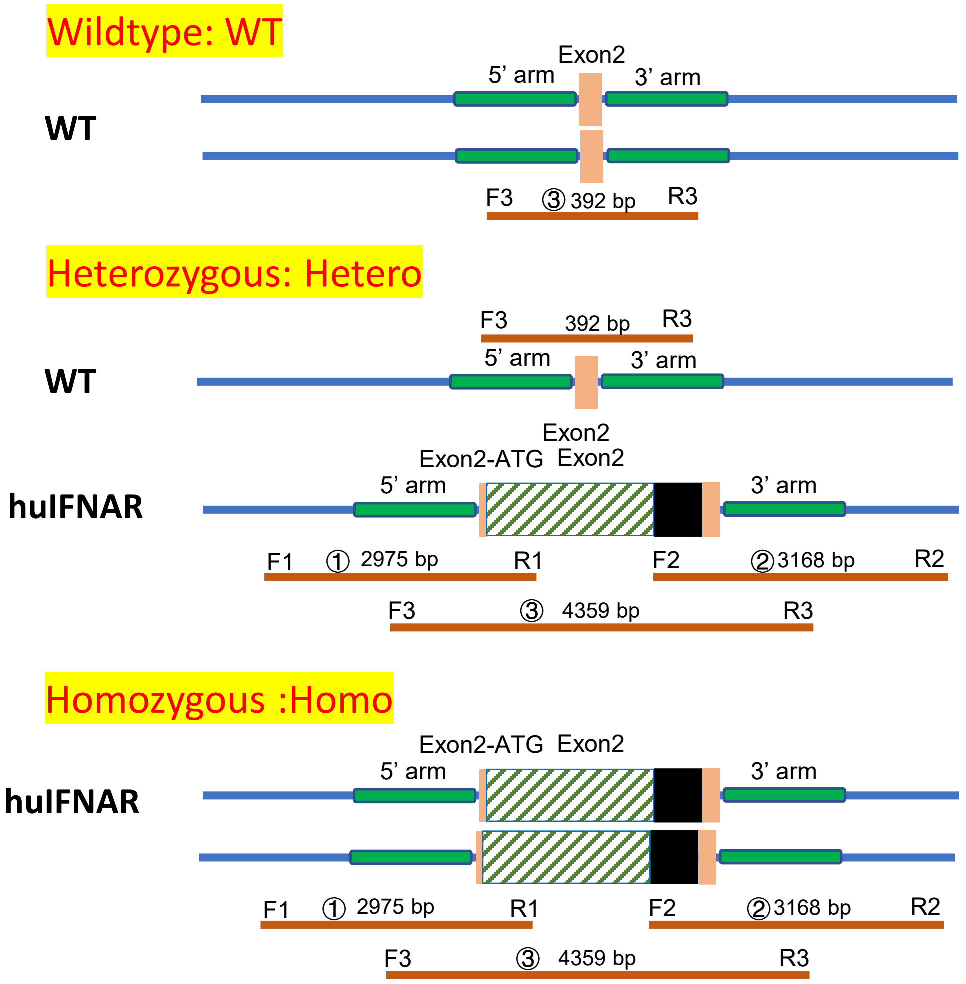

b

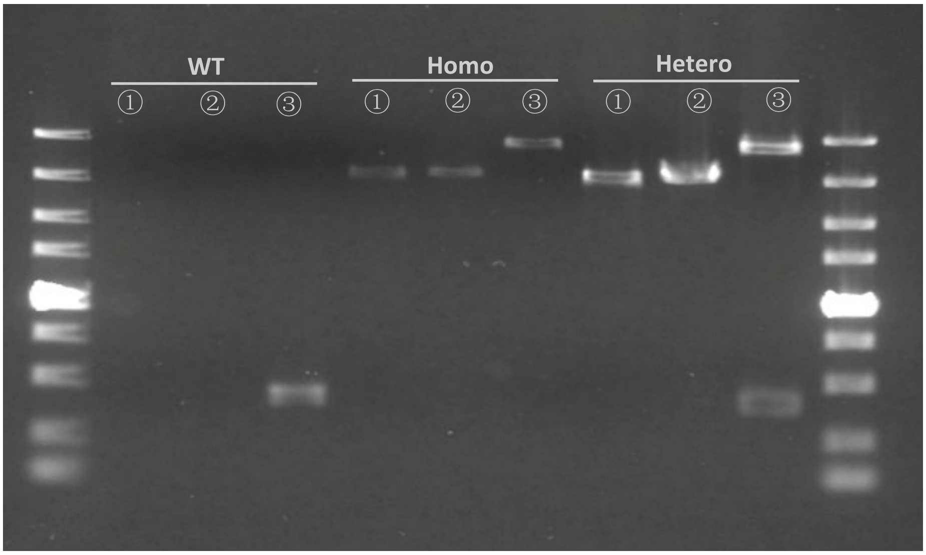

Supplementary Figure 4：The huIFNAR knock-in mouse genotyping. (**a**) An overview illustration of the genotyping strategy. Homologous recombination arms at the 5’ and 3’ of the knock-in fragment are highlighted. Primer sites and product sizes are labelled. (**b**) PCR results. WT, C57BL/6J wildtype mouse. Homo, homozygous C57BL/6J-huIFNAR mouse. Hetero, heterozygous C57BL/6J-huIFNAR mouse. DNA ladder, 5000, 3000, 2000, 1500, 1000, 750, 500, 250 and 100 bp. This PCR experiment has been repeated for 4 times having similar results.

Supp. Figure 5

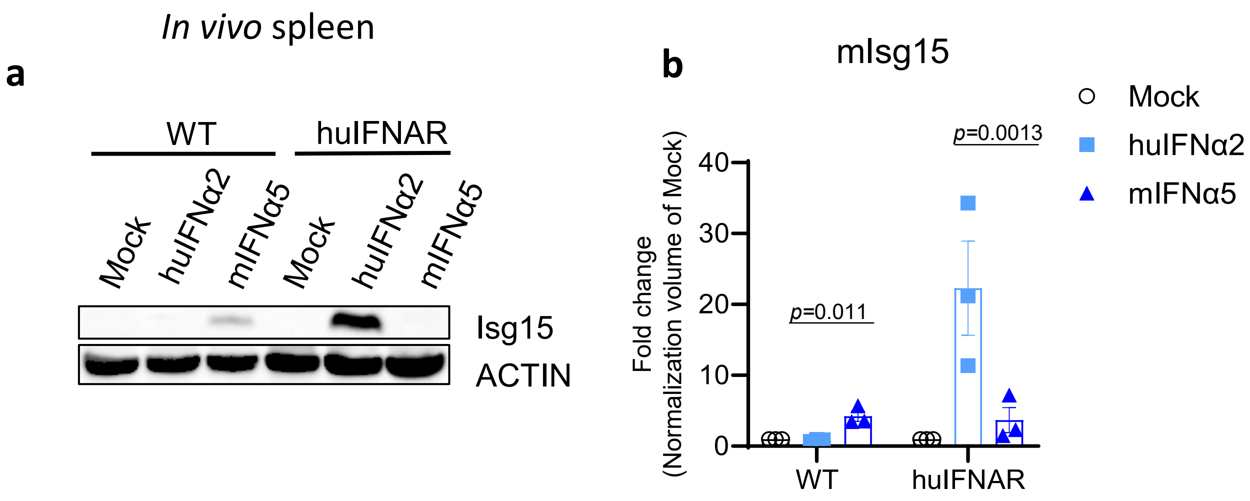

Supplementary Figure 5： Western blot analysis of mouse Isg15 proteins. (**a**) Mice were subcutaneously injected with huIFNα2 (2μg/mouse) and/or mIFNα5 (0.684μg/mouse). Mouse Isg15 levels were analyzed using Western blotting 16 hours post-injection (Blots were cropped and presented). This blotting experiment has been repeated for 3 times having similar results. (**b**) The bar graph represents a densitometric plot of mIsg15 protein expression across different groups. Data are means ± SEM, *p* values determined by two-sided one-way ANOVA. For each group, n=3 mice. Source data are provided as a Source Data file.

Supp. Figure 6

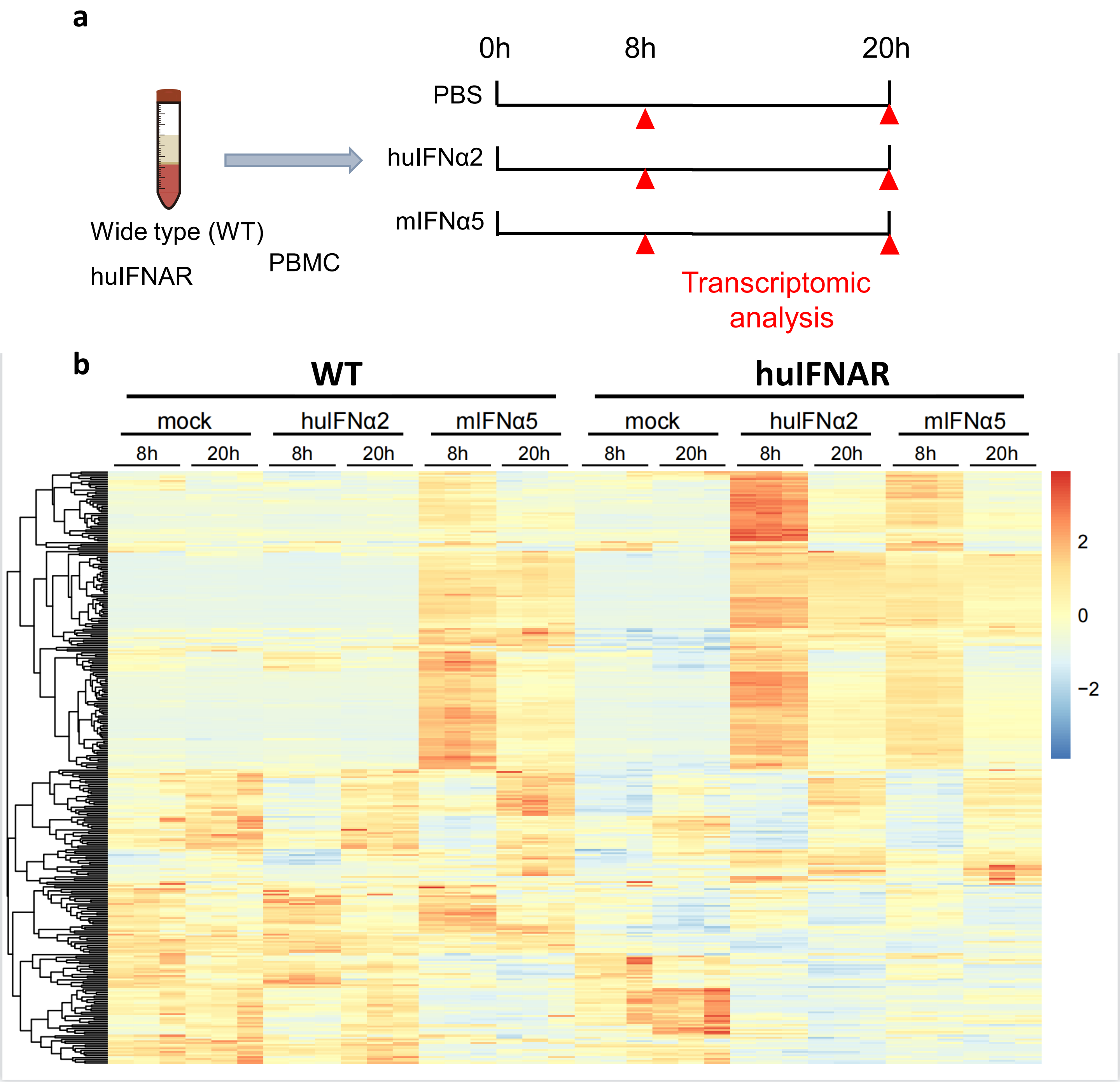

Supplementary Figure 6：Transcriptomic Response to Human and Mouse interferons in Wildtype and huIFNAR Mice. (**a**) Schematic of Experimental Approach. PBMCs from both wild-type and huIFNAR mice were isolated. These cells were then stimulated in vitro with either huIFNα2 or mIFNα5 for durations of 8 and 20 hours, respectively. Subsequent to this stimulation, transcriptome sequencing was performed. (**b**) The figure illustrates a heatmap that depicts the modulation of gene expression in interferon-stimulated genes (ISGs) within peripheral blood mononuclear cells (PBMCs) harvested from wildtype and huIFNAR mice. Cells were subjected to an in vitro stimulation period of 8 or 20 hours using either a saline solution (PBS; mock), human IFNα2 (huIFNα2, 800ng/ml), or mouse IFNα5 (mIFNα5, 275ng/ml). The color of the heatmap is determined by the normalized counts per million (CPM) values obtained from edgeR, providing a detailed representation of the differential gene expression across different treatment groups.

Supp. Figure 7

a

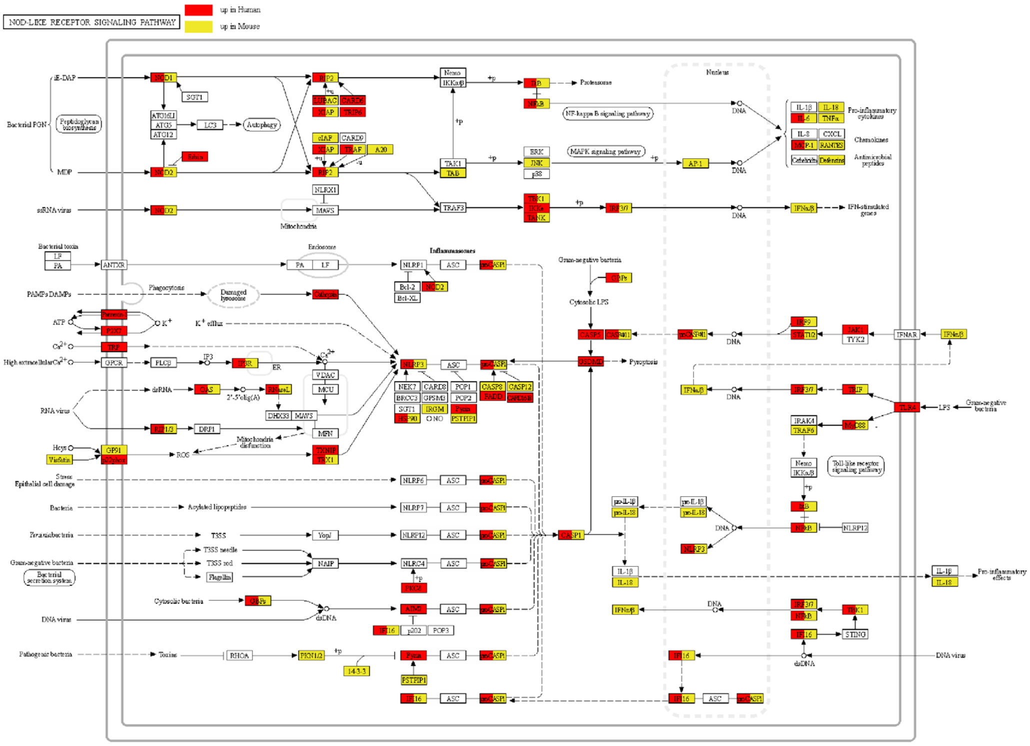

b

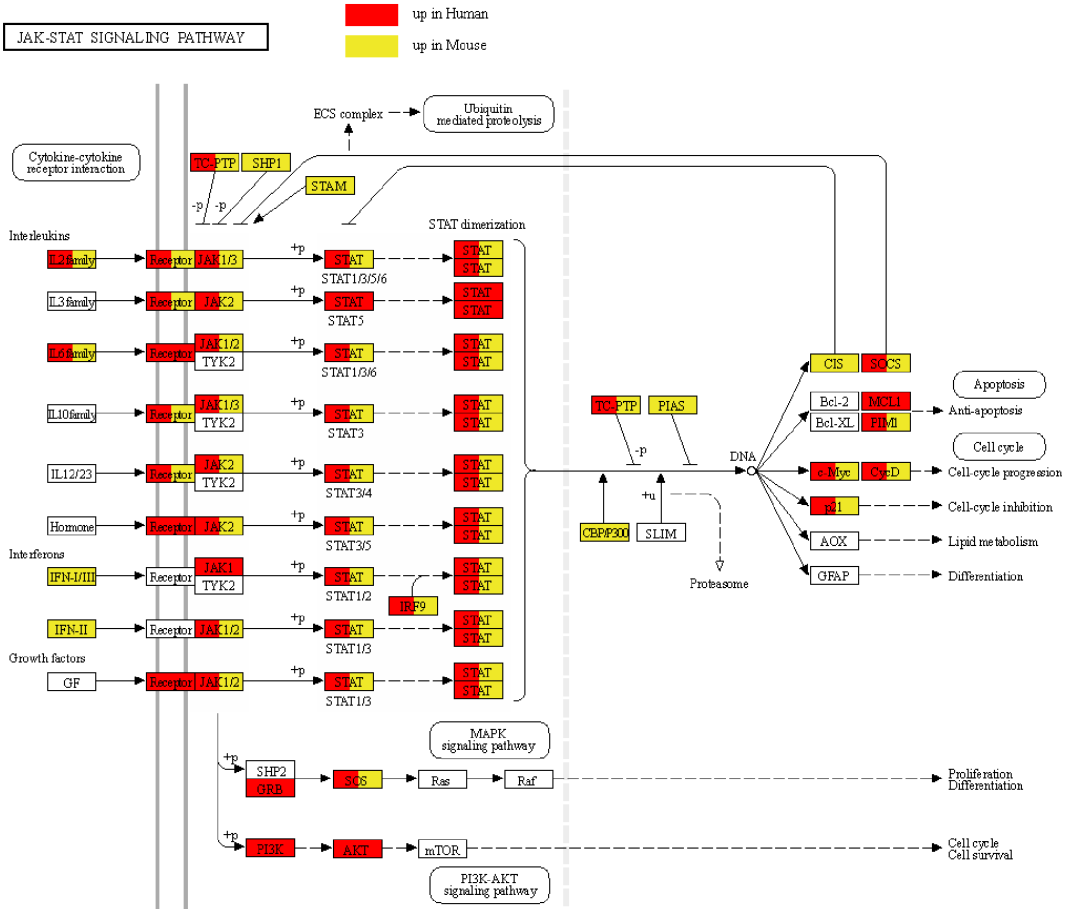

Supplementary Figure 7: Representative KEGG enrichment in response to human PEG-IFNa2 stimulation shared by both huIFNAR mouse and human PBMCs. (**a**) NOD-like receptor signaling pathway. (**b**) JAK-STAT signaling pathway. Red, up-regulated in human PBMCs. Yellow, up-regulated in huIFNAR mouse PBMCs.

Supp. Figure 8

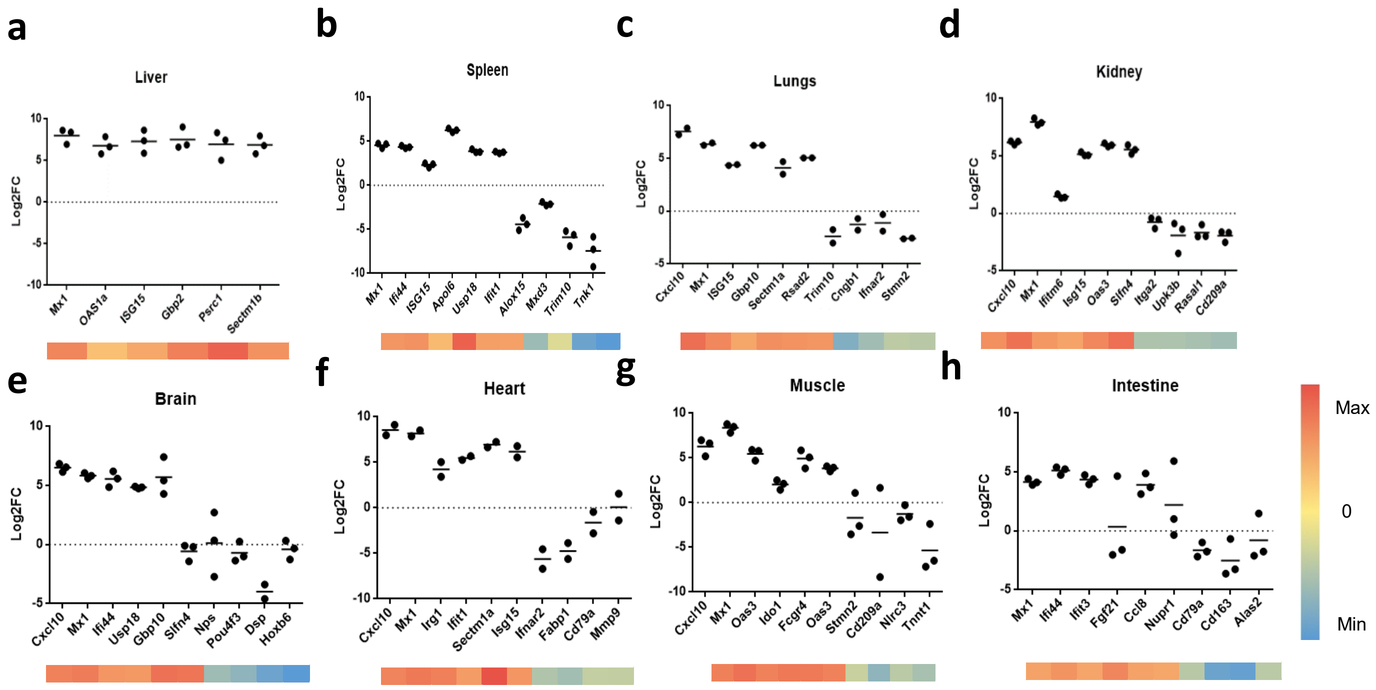

Supplementary Figure 8：Q-PCR confirmation of differentially expressed genes identified by RNA-seq results. The up-regulated (red) and down-regulated (blue) genes in RNA-seq data of each tissue were randomly verified by q-PCR as indicated. Data are presented as mean +/- SEM. Source data are provided as a Source Data file.

Supp. Fig. 9

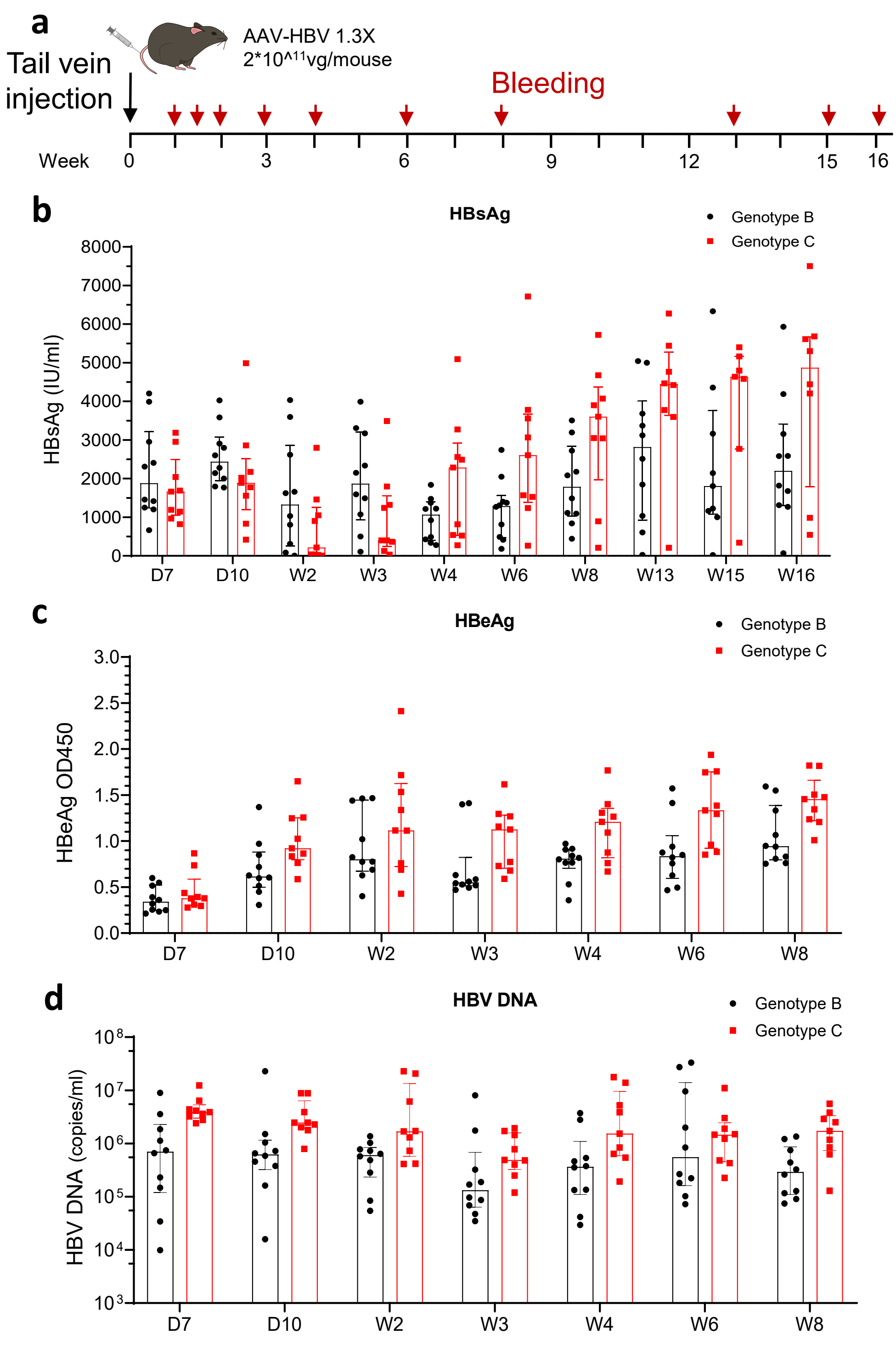

Supplementary Figure 9: Characterization of rAAV-1.3HBV mouse model with genotype B and C HBV. (**a**) Experiment outline. The recombinant adeno-associated virus rAAV8-1.3 HBV (2×10^11ug/mouse) with genotype B and C was injected into C57BL/6J mice (>8-week-old) through tail vein on day 0, respectively. Blood was collected from tail vein on day 7, day 10, and at week 2, week 3, week 4, week 6, week 8, week 13, week 15 and week 16 (termination time points). (**b**) Kinetics of HBsAg (IU/ml). (**c**) Kinetics of HBeAg (OD450). (**d**) Kinetics of HBV DNA (copies/ml). Genotype B, n=10. Genotype C, n=9. Data are presented as mean +/- SEM. Source data are provided as a Source Data file.

Supp. Fig. 10

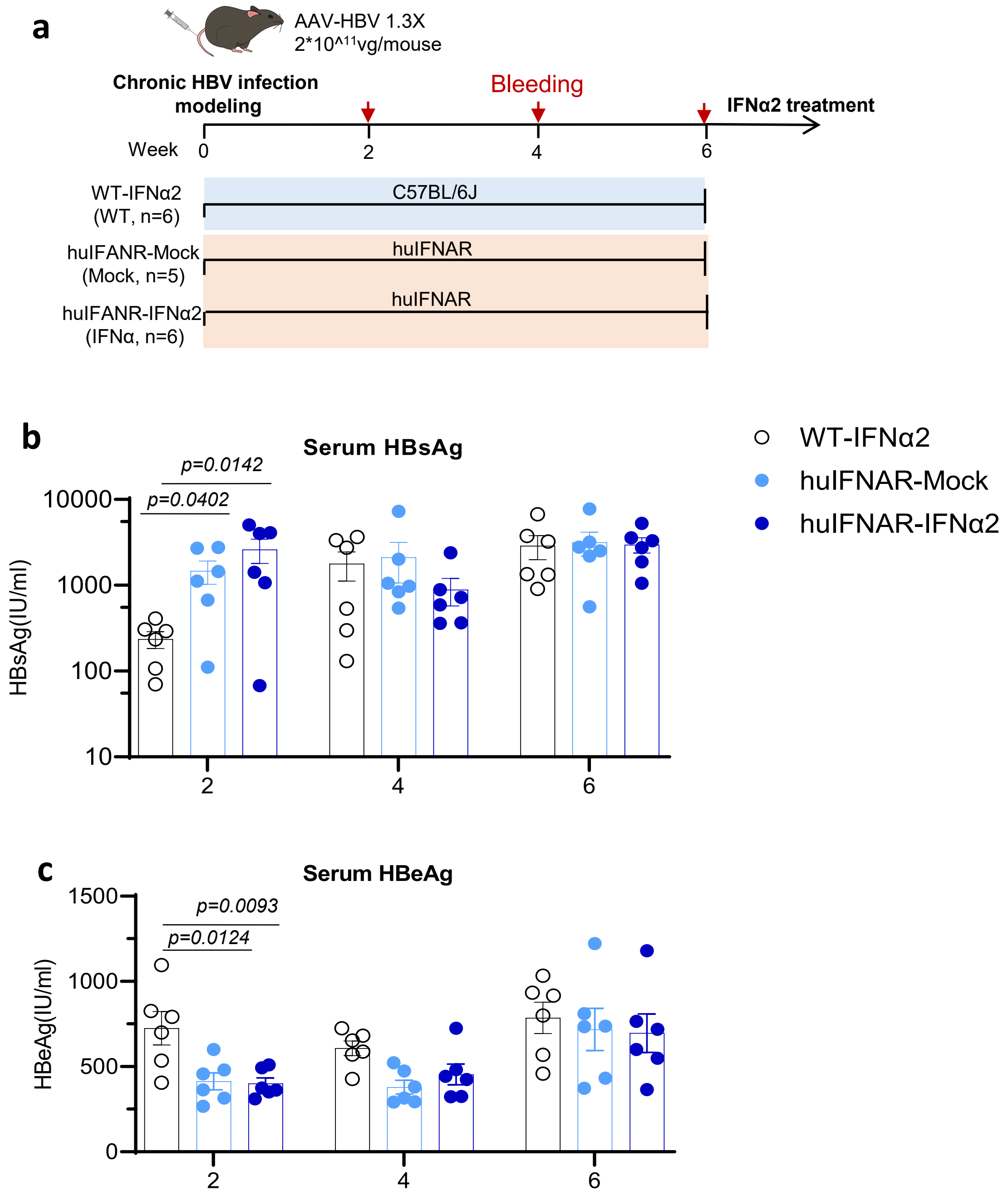

Supplementary Figure 10: Construction of the Chronic HBV infection huIFNAR AAV-HBV mouse model. (**a**) Experimental protocol. huIFNAR mouse model of chronic AAV-HBV infection was established by tail vein injection of a 1.3-fold HBV genome (AAV-1.3×HBV). After that, Peg-IFNα2(2ug/mouse) was injected. (**b**.**c**) Kinetics of HBsAg (IU/ml) and HBeAg (IU/ml)of each mouse prior to the IFNα2 treatment. Data are presented as means ± SEM, *p* values determined by one-way ANOVA. For each group, n=6 mice. Source data are provided as a Source Data file.

Supp. Fig.11

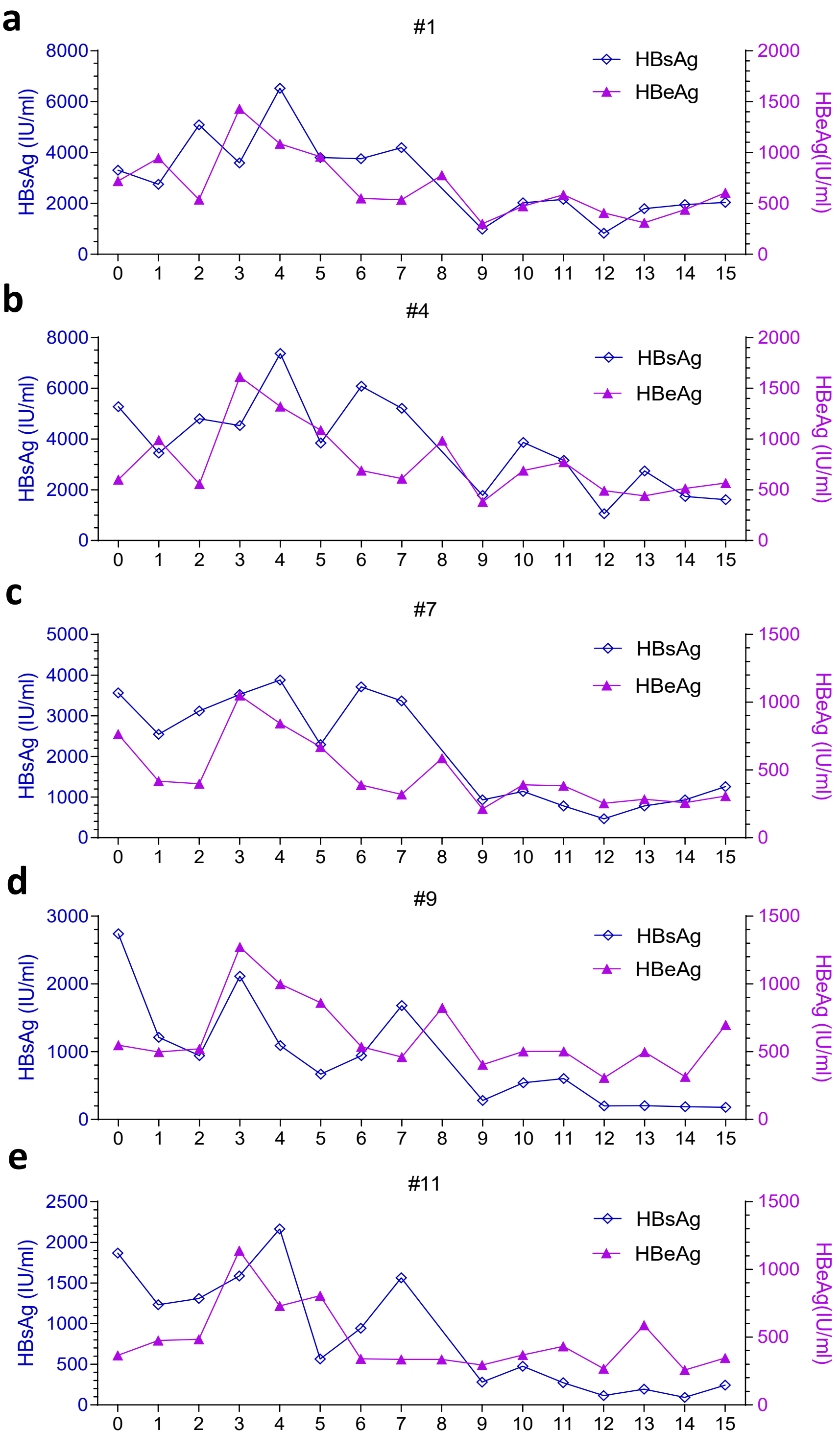

Supplementary Figure 11：The Kinetics of serum HBsAg and HBeAg of each mouse in IFNα2-treated huIFNAR group. HBsAg (IU/ml), blue, left Y-axis. HBeAg (IU/ml), purple, right Y-axis. Source data are provided as a Source Data file.

Supp. Fig. 12

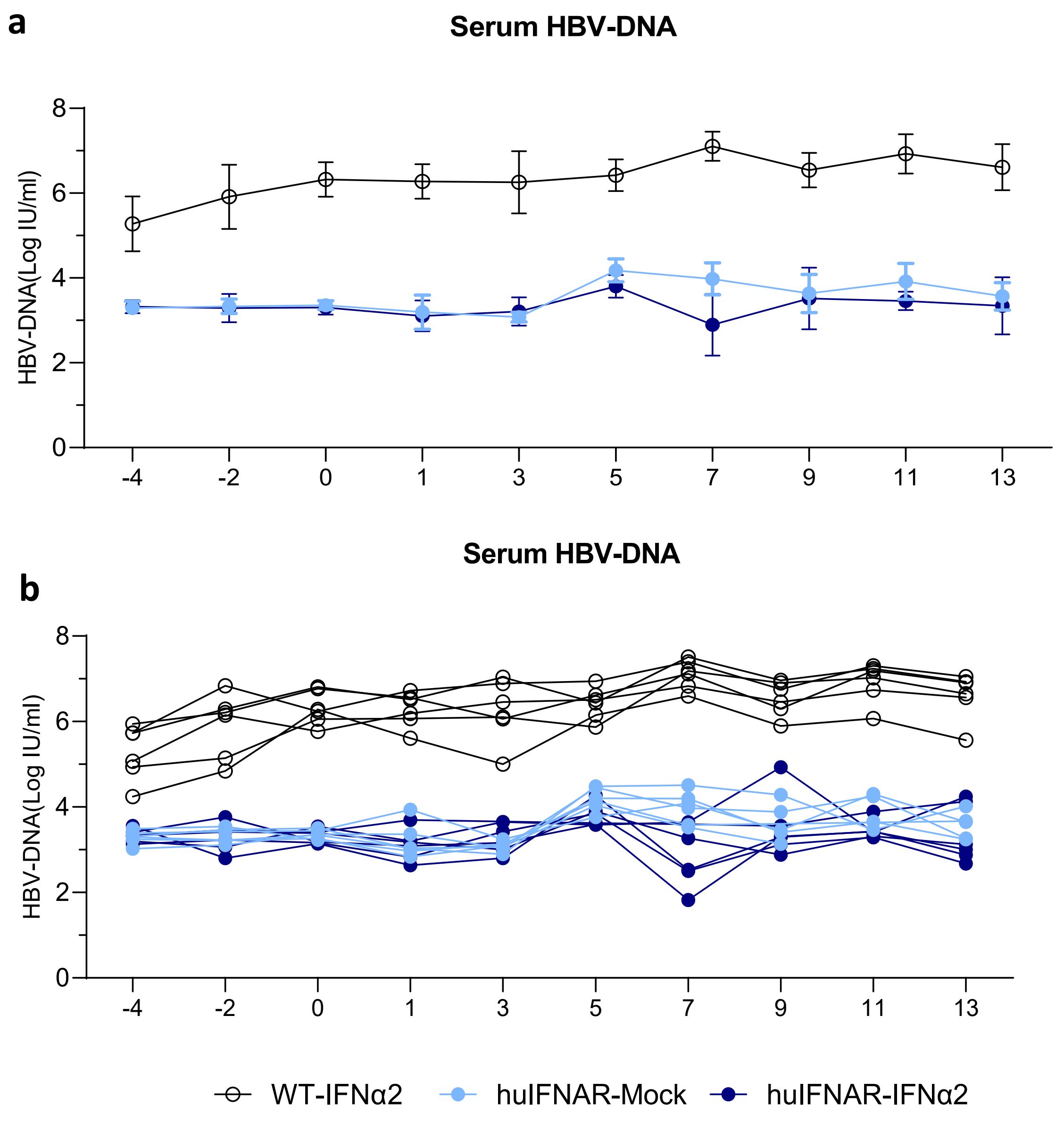

Supplementary Figure 12：The kinetics of serum HBV DNA between wildtype C57BL/6J and huIFNAR mouse. (**a**) The serum HBV DNA among C57BL/6J AAV-HBV IFNα treated wildtype group (WT-IFNα2, black, n=6), huIFNAR AAV-HBV mock-treated group (huIFNAR-Mock, light blue, n= 5) and PEG-IFNα2-treated group (huIFNAR-IFNα2, n=6). (**b**) Serum HBV DNA in the individual mouse as indicated in **a**. Data are presented as mean +/- SEM. Source data are provided as a Source Data file.

Supp. Fig. 13

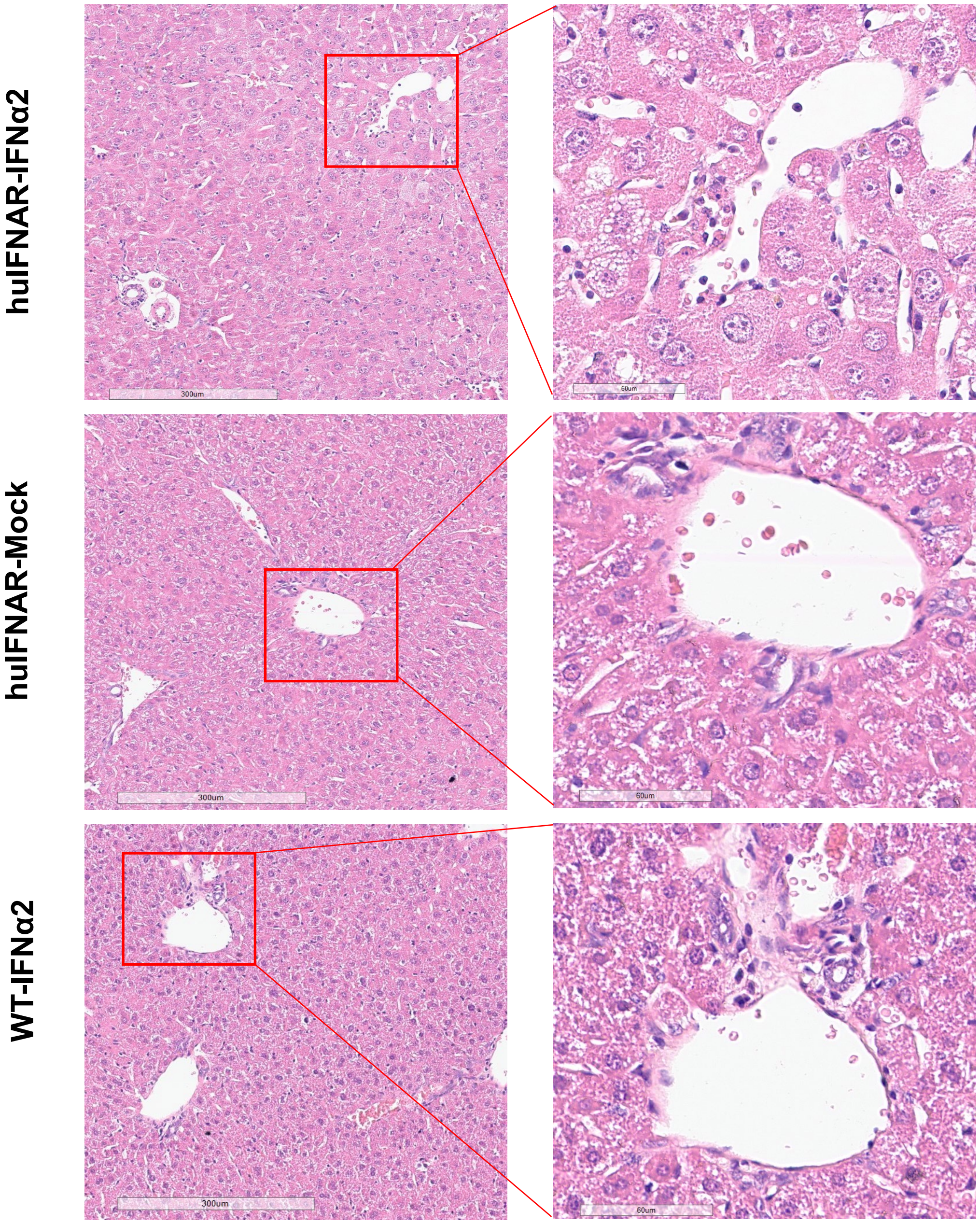

Supplementary Figure 13：Photomicrographs of liver sections stained with hematoxylin and eosin. Liver from the three groups all display normal hepatocytes arranged in cords, obvious sinusoids and central vein. This experiment has been repeated for 1 time, yielding similar results.

Supp. Fig. 14

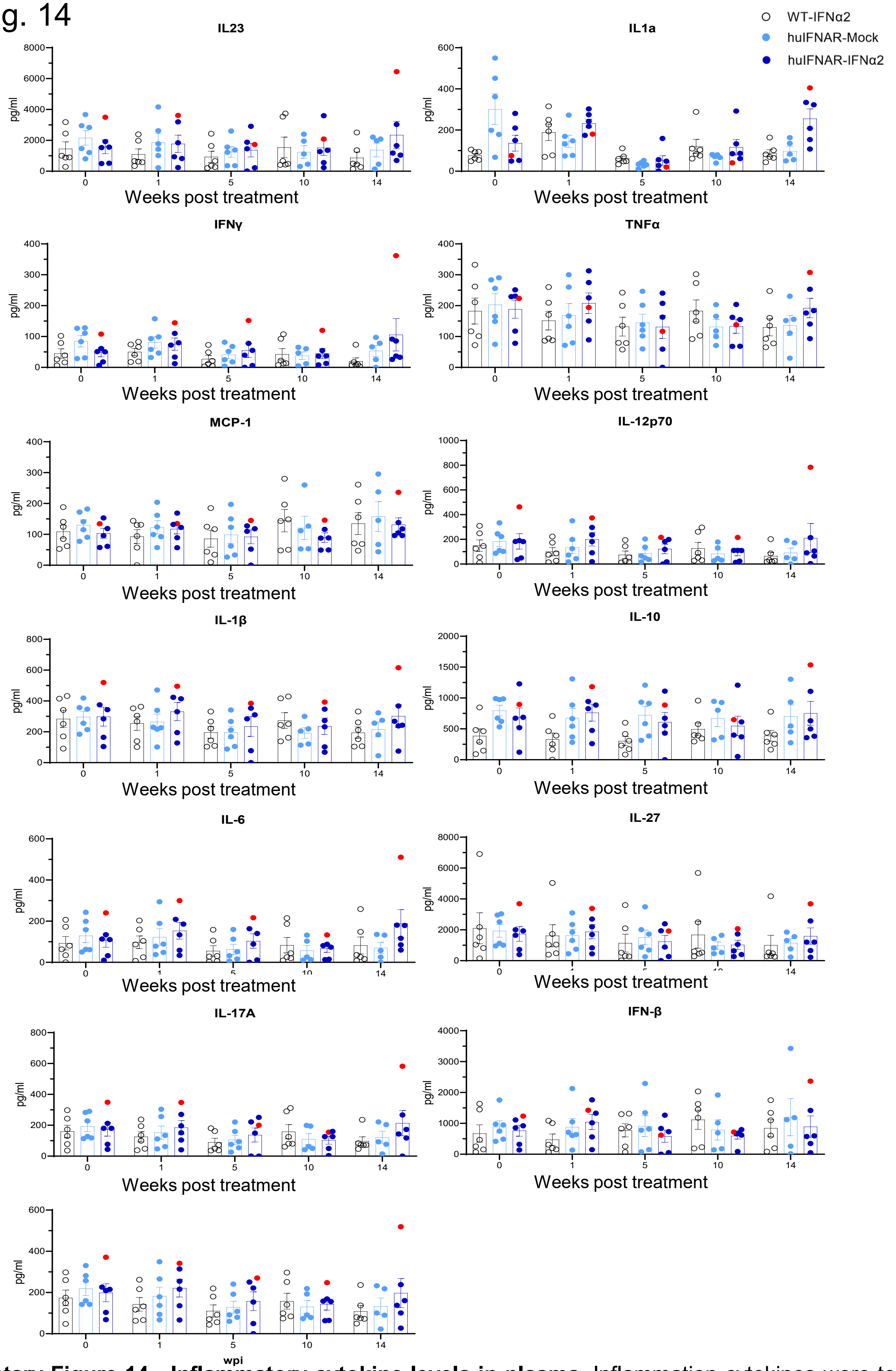

Supplementary Figure 14：Inflammatory cytokine levels in plasma. Inflammation cytokines were tested using Mouse Inflammation Panel (13-plex) with V-bottom Plate at 0, 1, 5, 10 and 14w.p.i., the dots in red represent mouse #3 in the huIFNAR-IFNα2 group. Bars represent the mean concentration of biological replicates ± SEM, p values determined by one-way ANOVA, but there is no statistical difference observed in each comparison. Source data are provided as a Source Data file.

Supp. Fig. 15

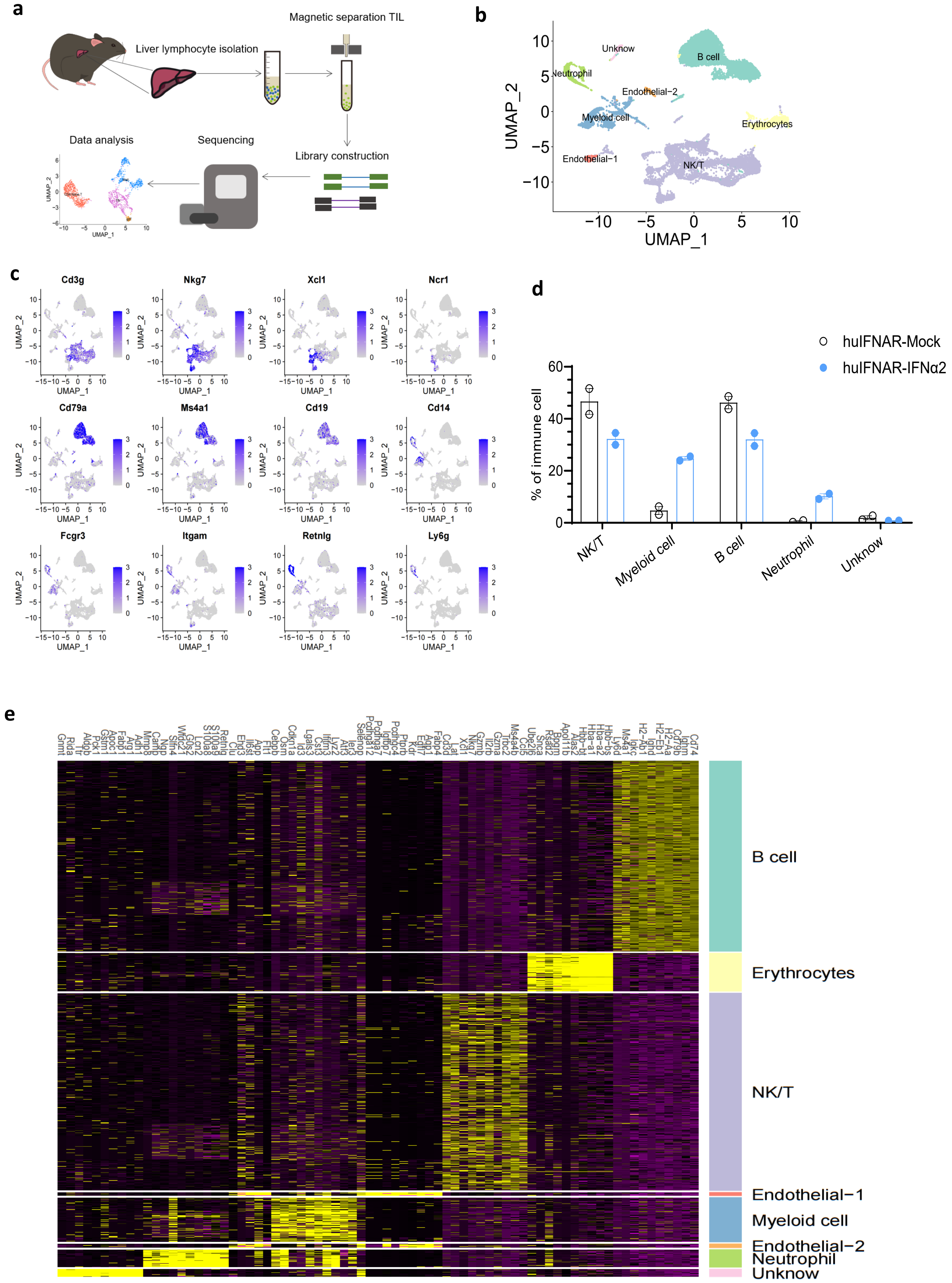

a
Liver lymphocyte isolation
Magnetic separation TIL
Library construction
Sequencing
Data analysis
b
UMAP_2
UMAP_1
Unknow
B cell
Neutrophil
Endothelial−2
Myeloid cell
Erythrocytes
Endothelial−1
NK/T
c
Cd3g
Nkg7
Xcl1
Ncr1
Cd79a
Ms4a1
Cd19
Cd14
Fcgr3
Itgam
Retnlg
Ly6g
d
huIFNAR-Mock
huIFNAR-IFNα2
% of immune cell
NK/T
Myeloid cell
B cell
Neutrophil
Unknow
e
B cell
Erythrocytes
NK/T
Endothelial−1
Myeloid cell
Endothelial−2
Neutrophil
Unknow

Supp. Fig. 15 Legend

Supplementary Figure 15: Constitution analysis of the intrahepatic immune cell profile with single-cell RNA-sequencing technology. (**a**) Graphic abstract of single-cell sorting pipeline. First, mouse intrahepatic immune cells were prepared. Then, the sequencing library was generated, followed by sequencing and analysis. (**b**) A UMAP plot of intrahepatic immune cells from all scRNA-seq samples, colored according to cell type. (**c**) Expression levels of selected representative genes in each cluster. (**d**) The proportion of each cluster between huIFANR-Mock and huIFANR-IFNα2 groups. Data are presented as mean +/- SEM. (**e**) Heatmap of top10 upregulated genes for each cluster by expression; each row represents a gene and each column represents a cell. The left bar indicates cluster ID. Selected differential genes are denoted on the top. Source data are provided as a Source Data file.

Supp. Fig. 16

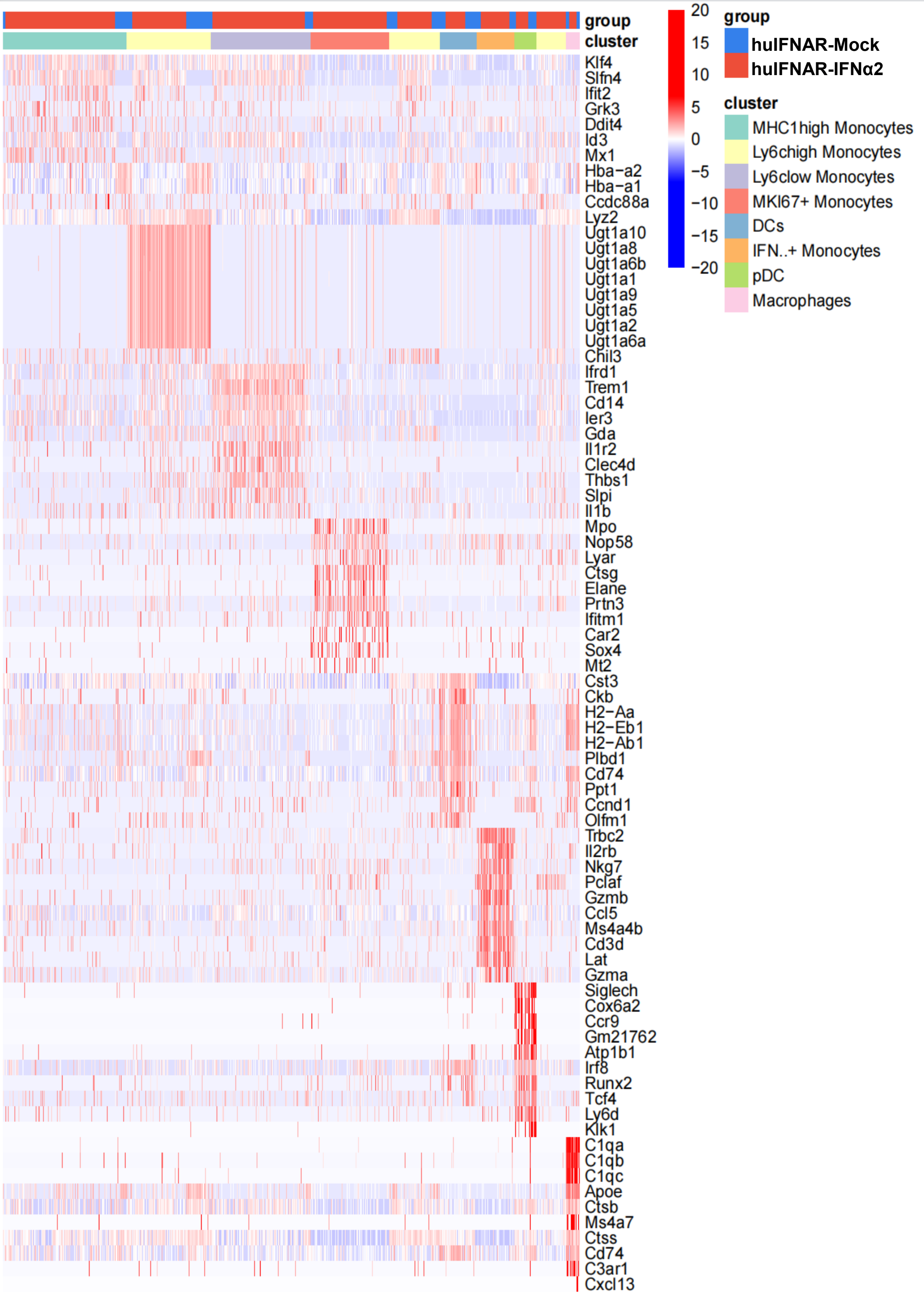

Supplementary Figure 16: Heatmap of eight myeloid cell clusters with unique signature genes. Information of clonal status is colored for each cell. The top bar indicates groups and cluster ID. Selected specifically expressed genes to each population are marked to the right column.

Supp. Fig. 17

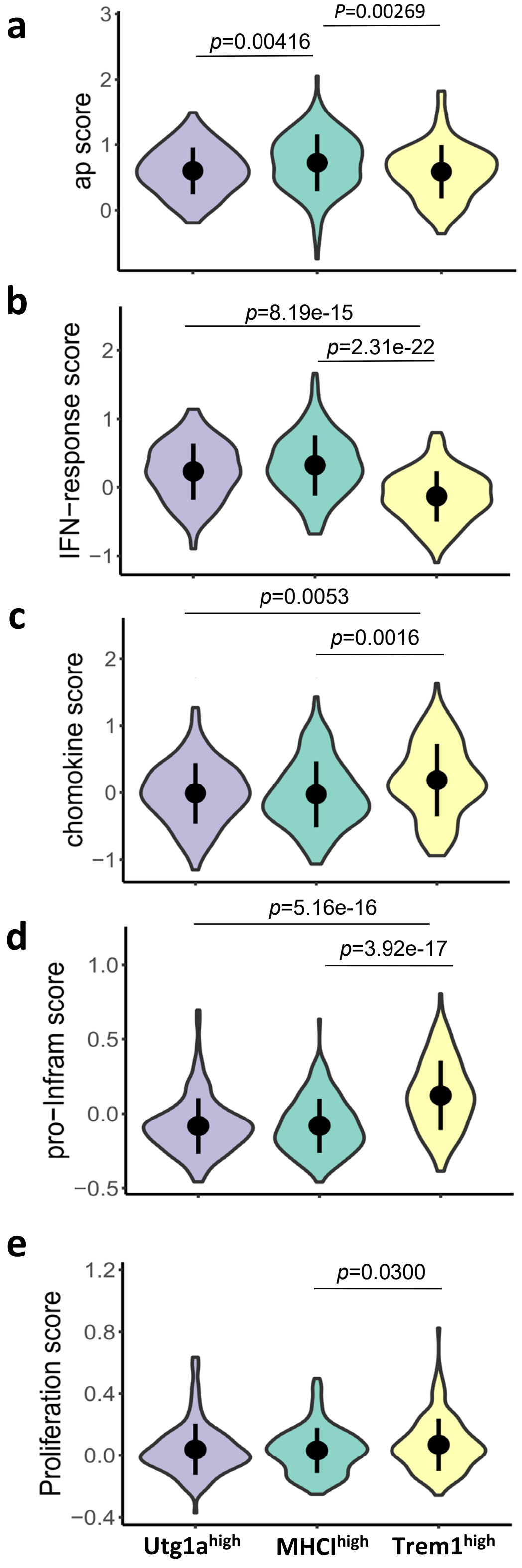

Supplementary Figure 17: Violin plots of gene set enrichment analysis scores of three clusters of Utg1a^{high}, MHC I^{high} and Trem1^{high}. (**a**) Antigen presentation score. (**b**) IFN responsiveness score. (**c**) Chemokine score. (**d**) Pro-inflammatory score. (**e**) Proliferation score. Data are presented as mean +/- SEM, and *p* values determined by one-way ANOVA test, adjusted for multiple comparisons, n=number of cell. Only *p*-values less than 0.05 are indicated.

Supp. Fig. 18

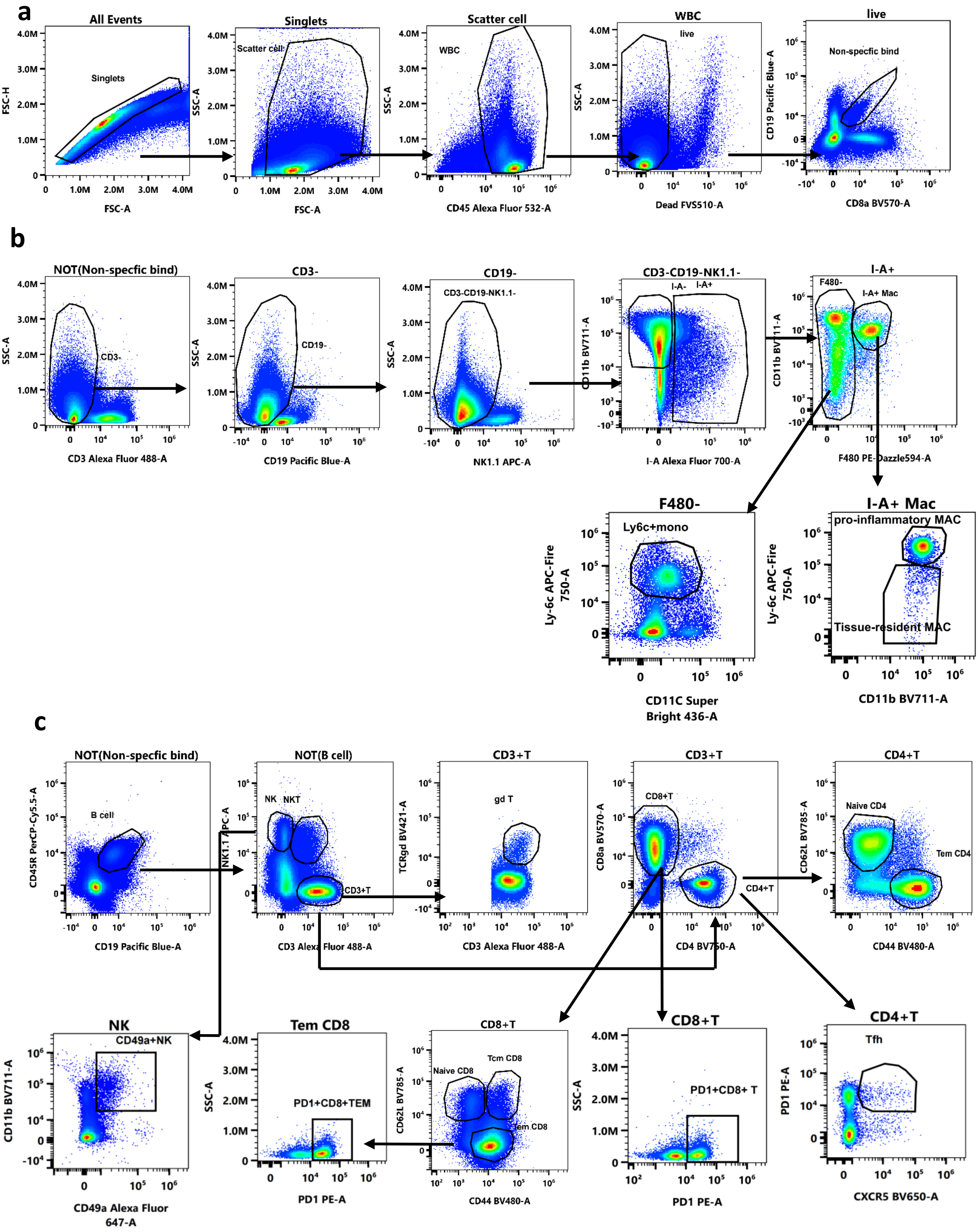

a
All Events
Singlets
FSC-H
FSC-A
Scatter cell
SSC-A
WBC
CD45 Alexa Fluor 532-A
live
Dead FVS510-A
Non-specfic bind
CD19 Pacific Blue-A
CD8a BV570-A
b
NOT(Non-specfic bind)
CD3-
CD3 Alexa Fluor 488-A
CD19-
CD19 Pacific Blue-A
CD3-CD19-NK1.1-
NK1.1 APC-A
I-A-
I-A+
CD11b BV711-A
I-A Alexa Fluor 700-A
F480-
I-A+ Mac
F480 PE-Dazzle594-A
Ly6c+mono
Ly-6c APC-Fire 750-A
CD11C Super Bright 436-A
pro-inflammatory MAC
Tissue-resident MAC
CD11b BV711-A
c
B cell
CD45R PerCP-Cy5.5-A
NOT(B cell)
NK
NKT
CD3+T
NK1.1 APC-A
gd T
TCRgd BV421-A
CD8+T
CD4+T
CD8a BV570-A
CD4 BV750-A
Naive CD4
Tem CD4
CD62L BV785-A
CD44 BV480-A
NK
CD49a+NK
CD49a Alexa Fluor 647-A
Tem CD8
PD1+CD8+TEM
PD1 PE-A
Naive CD8
Tcm CD8
Tem CD8
PD1+CD8+ T
Tfh
PD1 PE-A
CXCR5 BV650-A

Supp. Fig. 18 Legend

Supplementary Figure 18: Gating strategy of a 20-parameter panel for flow cytometry analysis of the intrahepatic immune cells.(**a**) Flow cytometry quality control. Single cells were selected (FSC-H vs. FSC-A) before gating on live (FVS510) $CD45^+$ lymphocytes. The non-specific binding signals of BV570-CD19 and Pacific Blue-CD8α double positive were removed. (**b**) Gating strategy of mouse myeloid cell subsets in the liver. Identification of monocytes as well as of Macrophages. After excluding any cells expressing either CD3, CD19 or NK1.1, $MHCII^+$ macrophages were identified within the subset expressing F480 and/or CD11b. Ly6c and CD11c were also selected for subsequent phenotypic analysis. Gating on $MHCII^+$ $F480^-$ cells is followed by discrimination of $Ly6c^+$ monocytes. (**c**) Gating strategy of mouse CD4 and CD8 T cell subsets from the liver. Exclusion of B cells, NK cells and NKT cells and gating on $CD3\varepsilon^+$ cells and $CD4^+CD8\alpha^-$ cells ($CD4^+T$) or $CD4^-CD8\alpha^+$ cells ($CD8^+T$). Naïve, effector and memory T cell populations can then be defined within CD8 T cells using CD44 and CD62L expression to identify $CD44^{lo}CD62L^{hi}$ naïve cells, $CD44^{hi}CD62L^{hi}$ central memory cells, and $CD44^{hi}CD62L^{lo}$ effector memory and effector cells. The expression of the Inhibitory factor Pd1 is detected in CD8+T and CD8+TEM($CD44^{hi}CD62L^{lo}$).

Supp. Fig. 19

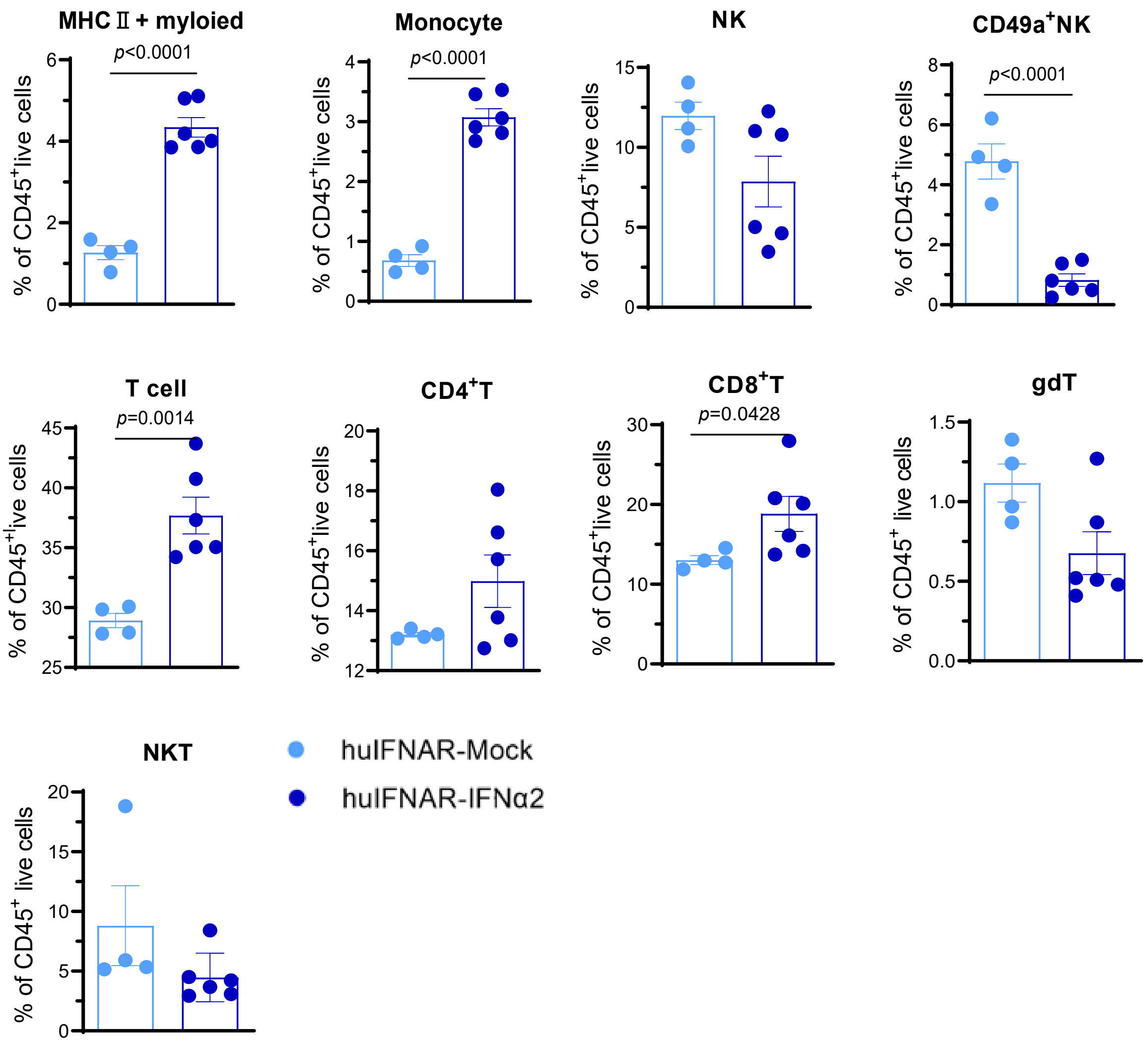

Supplementary Figure 19: Flowcytometry analysis of the intrahepatic immune cells. The frequencies of intrahepatic MHC II+ myeloid, monocyte, NK, CD49a+NK, T Cells, CD4+T, CD8+T, GammaDelta (gd) T and NKT Cells between huIFNAR-Mock (light blue,n=4) and huIFNAR-IFNα2 groups (dark blue, n=6) are indicated, respectively. Data are presented as mean +/- SEM, and *p* values are determined by two-tailed, unpaired, Student's t-test. Source data are provided as a Source Data file.

Supp. Fig. 20

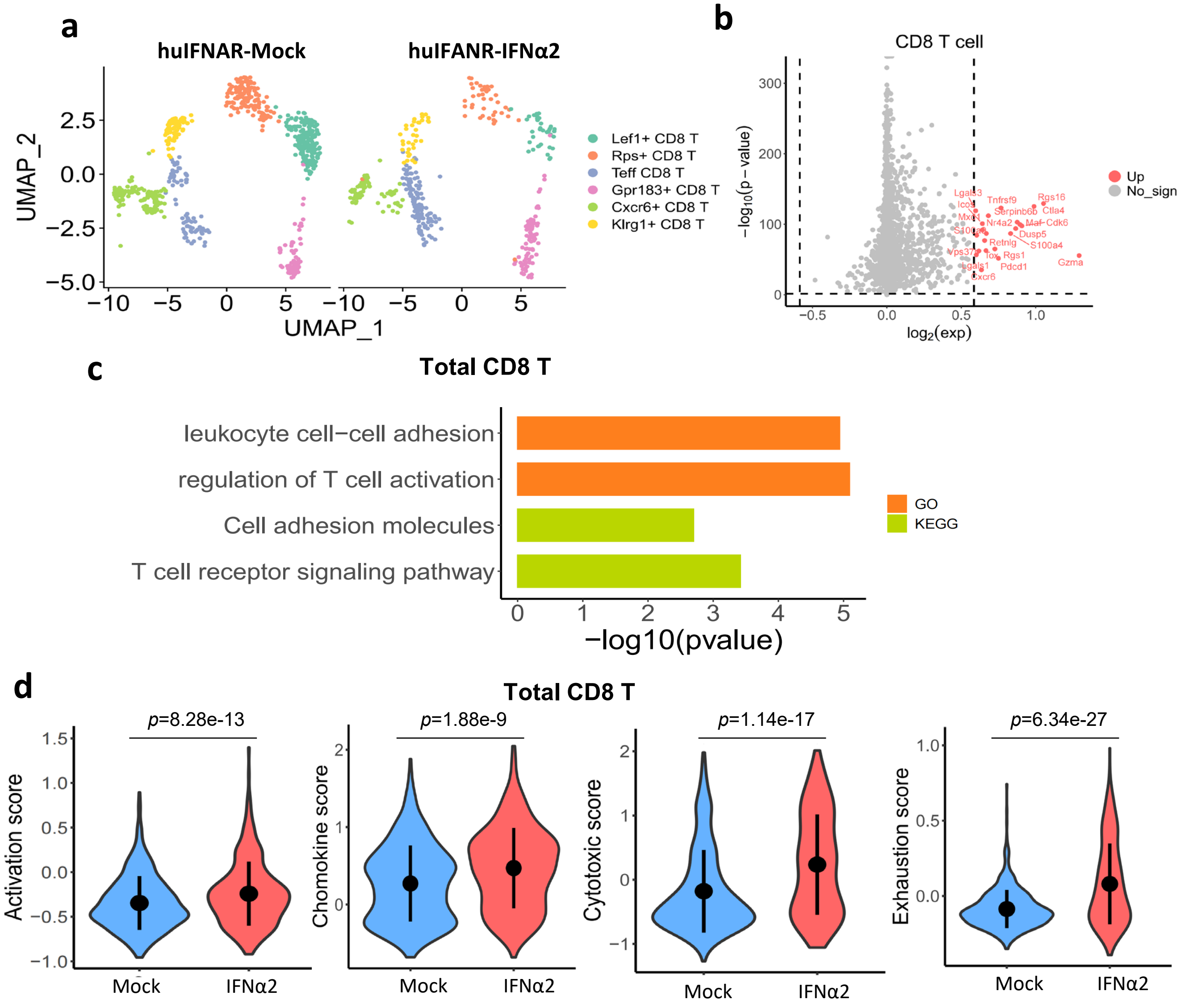

Supplementary Figure 20: Characterization of the intrahepatic CD8⁺T cells by single-cell RNA-sequencing. (**a**) UMAP split by condition, huIFNAR-Mock (left) and huIFANR-IFNα2 (right). (**b**) Volcano plots showing differentially expressed genes between Mock and IFNα2 mice in CD8⁺T cells. positive log2 fold change indicates upregulation in IFNα2 (red) relative to Mock mice. Non-significantly differentially expressed genes (*p*>0.05) or genes with a log2 fold change between -0.5 and 0.5 are shown in grey. (**c**) GO and KEGG Biological Process analysis of differentially expressed genes in CD8⁺T cells. Top 2 significantly altered pathways are presented. (**d**) Violin plots showing gene set enrichment analysis scores of huIFNAR-Mock and of huIFANR-IFNα2 in activation, chemokine, cytotoxic and exhaustion scores. *p* values determined by two-tailed, unpaired, Student's t-test, n=number of cell. Data are presented as mean +/- SEM.

Supp. Fig. 21

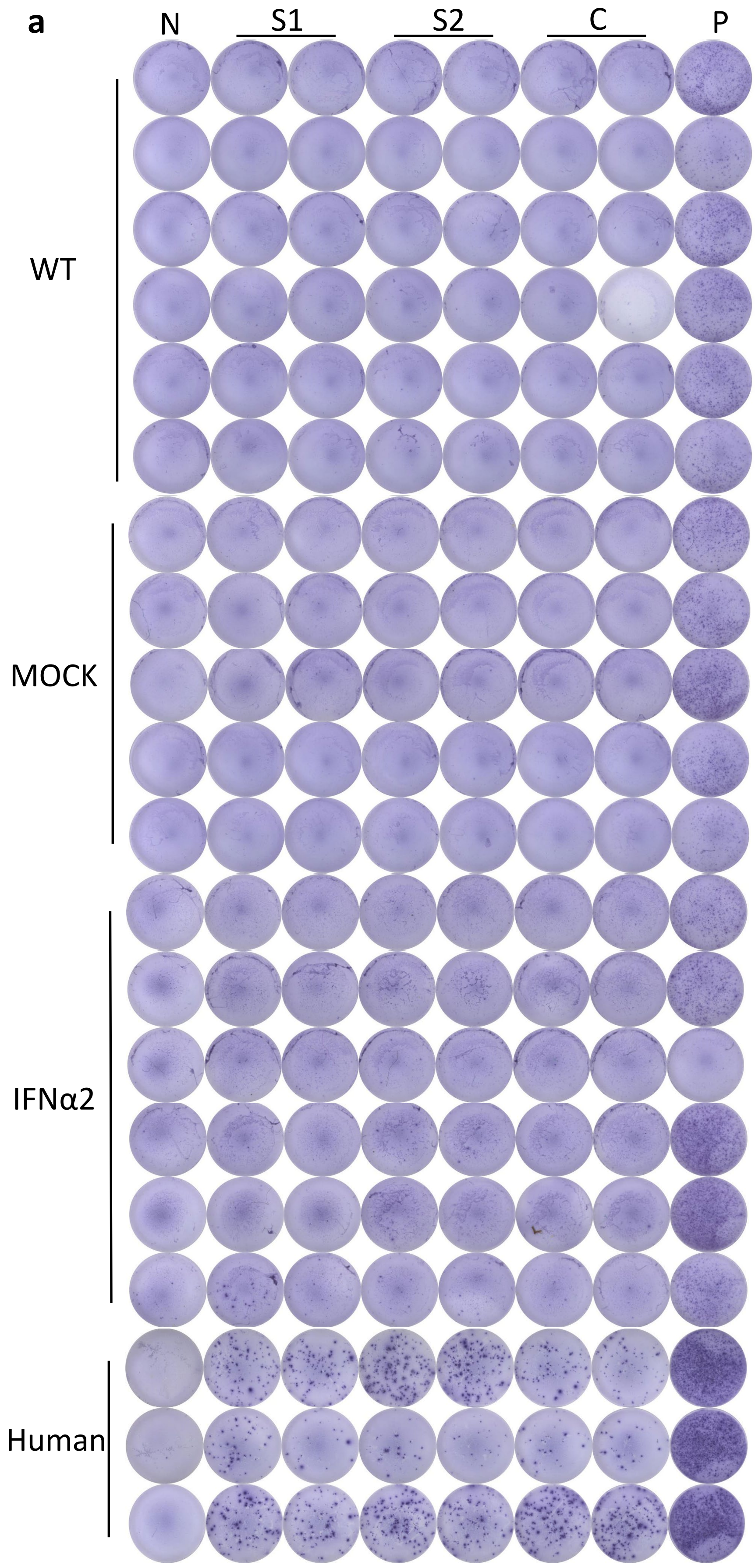

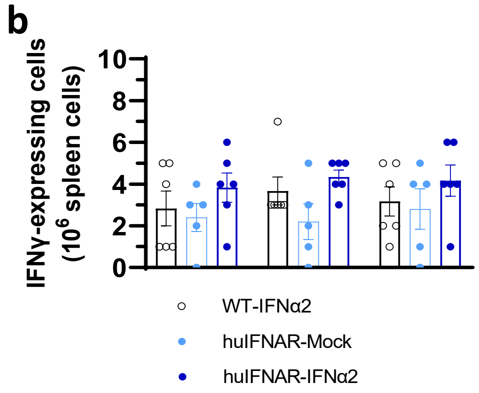

Supplementary Figure 21: ELISpot analysis of IFNγ secretion in mouse splenocytes as indicated. (**a**) Sample wells with spots with mock or HBV peptide stimulation. Each condition was performed in duplicates. (**b**) Splenocytes were collected after 15 weeks after IFNα2 injected and the number of IFNγ secreting cells generated in response to S1, S2 and core peptides; Frequencies of HBV peptide-specific spots per million splenocytes in WT-IFNα2, huIFANR-Mock or huIFANR-IFNα2 mice. Data in are means ± SEM (n= mock:IFNα2-treated = 4:6;), Data are means ± SEM , *p* values determined by one-way ANOVA. Source data are provided as a Source Data file.
