# Supplementary material for: Interferon stimulated immune profile changes in a humanized mouse model of HBV infection
Source: Nat Commun. 2023 Nov 15;14:7393. doi: 10.1038/s41467-023-43078-5 (PMC10652013; doi:10.1038/s41467-023-43078-5)
Supplement: Supplementary file 3 — Description of Additional Supplementary Files [file 41467_2023_43078_MOESM3_ESM.pdf]

Supplementary Data 1 Legend

Humanized IFNAR Cas9-KI Targeted vector sequence information

Recombinant Arms is Red, Knock-in fragment is yellow, exon2 is blue, polyA is gray.

Supplementary Data 2 Legend

Humanized IFNAR Sanger-sequencing information

Gene sequencing results validating the successful generation of homozygous human interferon receptor humanized (huIFNAR) mice through CRISPR/Cas9-assisted pronuclear microinjection.

Supplementary Data 3 Legend

Comprehensive GO details associated with Fig. 2

The initial sheet, titled “Figure 2d Data,” showcases the top 30 most enriched GO terms, alongside the calculated gene ratio, *p*-value, and gene counts for all biological processes (BP) in both the human and mouse groups. Subsequent sheets provide insights into the immune and metabolic-associated sub GO terms, mirroring the data presented in Figure 2d.
