# Supplementary material for: Interferon stimulated immune profile changes in a humanized mouse model of HBV infection
Source: Nat Commun. 2023 Nov 15;14:7393. doi: 10.1038/s41467-023-43078-5 (PMC10652013; doi:10.1038/s41467-023-43078-5)
Supplement: Supplementary file 4 — Supplementary Data 1 [file 41467_2023_43078_MOESM4_ESM.pdf]

Supplementary Data 1

Humanized IFNAR Cas9-KI Targeted vector sequence information

Recombinant Arms is Red, Knock-in fragment is yellow, exon2 is blue, polyA is gray.

ACCAGA ATTTGG CTACTT TTTAAT TTATTT TTAAAG ACAGGT AGCTCC TCTAGC TGGCTT CAAACT
TGCTAT GTAGCC GAGGAT GACCTT GAATGC CTGACC TTTCCC CCACCT CTCTGC TTAGAG GACAGA
TGTGAC ACGCAC AGTAAA CTCATG CAAGTT TAACCC TAATCC TAACCA ATCCAG GGCTAC CACGGG
GCCGCA TCTGCA GCTAAA TCTGGC TCGTTC TTACTC GTCTCT CGTTAG CGTGTA TGTGTC TATCAT
GTAAAT TACAAT ATAATT GGGTGC TTCTGA GTTTTG ACCAAC TCAATA TTGATC TCTTTC AGGTGT
GAGAGC AGAAAA ACGGAC TTAAGA GCTGAG CAGGAT GCTGCT GTCCCA GAATGC CTTCAT CTTCCG
CTCCCT GAACCT GGTGCT GATGGT CTACAT CTCCCT GGTCTT TGGCAT CTCCTA TGACTC CCCTGA
CTACAC AGATGA GTCCTG CACCTT CAAGAT CTCCCT GAGGAA CTTCAG GTCCAT CCTGTC CTGGGA
GCTGAA GAACCA CTCCAT TGTGCC CACCCA CTACAC CCTGCT GTACAC CATCAT GTCCAA GCCTGA
GGACCT GAAGGT GGTGAA GAACTG TGCCAA CACCAC CAGGTC CTTCTG TGACCT GACCGA TGAGTG
GAGGTC CACCCA TGAGGC CTATGT GACAGT GCTGGA GGGCTT CTCTGG CAACAC CACCCT GTTCTC
CTGCTC CCACAA CTTCTG GCTGGC CATTGA CATGTC CTTTGA GCCCCC TGAGTT TGAGAT TGTGGG
CTTCAC CAACCA CATCAA TGTGAT GGTGAA GTTCCC ATCCAT TGTGGA GGAGGA GCTGCA GTTTGA
CCTGTC CCTGGT GATTGA GGAGCA GTCTGA GGGCAT TGTGAA GAAGCA CAAGCC TGAGAT CAAGGG
CAACAT GTCTGG CAACTT CACCTA CATCAT TGACAA GCTGAT CCCCAA CACCAA CTACTG TGTCTC
TGTCTA CCTGGA GCACTC TGATGA GCAGGC TGTGAT CAAGTC CCCCCT GAAGTG CACCCT GCTGCC
CCCTGG CCAGGA GTCTGA GTCTGC TGAGTC TGCCAT TGTGGG CATCAC CACCTC CTGCCT GGTGGT
GATGGT CTTTGT CTCCAC CATTGT GATGCT GAAGAG GATTGG CTACAT CTGCCT GAAGGA CAACCT
GCCCAA TGTGCT GAACTT CAGGCA CTTCCT GACCTG GATCAT CCCTGA GAGGTC CCCATC TGAGGC
CATTGA CAGGCT GGAGAT CATCCC CACCAA CAAGAA GAAGAG GCTGTG GAACTA TGACTA TGAGGA
TGGCTC TGACTC TGATGA GGAGGT GCCCAC AGCCTC TGTGAC AGGCTA CACCAT GCATGG CCTGAC
AGGCAA GCCCCT GCAGCA GACCTC TGACAC CTCTGC CTCCCC TGAGGA CCCCCT GCATGA GGAGGA
CTCTGG CGCTGA GGAGTC TGATGA GGCTGG CGCTGG CGCTGG CGCTGA GCCTGA GCTGCC CACAGA
GGCTGG CGCTGG CCCATC TGAGGA CCCCAC AGGCCC ATATGA GAGGAG GAAGTC TGTGCT GGAGGA
CTCCTT CCCCAG GGAGGA CAACTC CTCCAT GGATGA GCCTGG CGACAA CATCAT CTTCAA TGTGAA
CCTGAA CTCTGT CTTCCT GAGGGT GCTGCA TGATGA GGATGC CTCTGA GACCCT GTCCCT GGAGGA
GGACAC CATCCT GCTGGA TGAGGG TCCCCA GAGGAC AGAGTC TGACCT GAGGAT TGCTGG CGGCGA
CAGGAC CCAGCC CCCCCT GCCATC CCTGCC ATCCCA AGACCT GTGGAC AGAGGA TGGCTC CTCTGA
GAAGTC TGACAC CTCTGA CTCTGA TGCTGA TGTGGG CGATGG CTACAT CATGAG GGGATC CGGCTC
TGGCTC TGGCTC TGGCTC TGGCTC TGGCGC CACCAA CTTCTC CCTGCT GAAGCA GGCTGG CGATGT
GGAGGA GAACCC TGGGCC CATGAT GGTGGT GCTGCT GGGCGC CACCAC CCTGGT GCTGGT GGCTGT
GGCCCC ATGGGT GCTGTC TGCTGC TGCTGG CGGCAA GAACCT GAAGTC CCCCCA GAAGGT GGAGGT
GGACAT CATTGA TGACAA CTTCAT CCTGAG GTGGAA CAGGTC TGATGA GTCTGT GGGCAA TGTGAC
CTTCTC CTTTGA CTACCA GAAGAC AGGCAT GGACAA CTGGAT CAAGCT GTCTGG CTGCCA GAACAT
CACCTC CACCAA GTGCAA CTTCTC CTCCCT GAAACT GAATGT CTATGA GGAGAT CAAGCT GAGGAT
CAGGGC TGAGAA GGAGAA CACCTC CTCCTG GTATGA GGTGGA CTCCTT CACCCC ATTCCG CAAGGC

CCAGAT TGGCCC CCCTGA AGTGCA TCTGGA GGCTGA GGACAA GGCCAT TGTGAT CCACAT CTCCCC
TGGCAC CAAGGA CTCTGT GATGTG GGCTCT GGATGG CCTGTC CTTCAC CTACTC CCTGGT GATCTG
GAAGAA CTCCTC TGGCGT GGAGGA GAGGAT TGAGAA CATCTA CTCCAG GCACAA GATCTA CAAGCT
GTCCCC TGAGAC CACCTA CTGCCT GAAGGT GAAGGC TGCCCT GCTGAC CTCCTG GAAGAT TGGCGT
CTACTC CCCTGT GCACTG CATCAA GACCAC AGTGGA GAATGA GCTGCC CCCCCC TGAGAA CATTGA
GGTCTC TGTGCA GAACCA GAACTA TGTGCT GAAGTG GGACTA CACCTA TGCCAA CATGAC CTTCCA
AGTGCA GTGGCT GCATGC CTTCCT GAAGAG GAACCC TGGCAA CCATCT GTACAA GTGGAA GCAGAT
CCCTGA CTGTGA GAATGT GAAGAC CACCCA GTGTGT CTTCCC CCAAAA TGTCTT CCAGAA GGGCAT
CTACCT GCTGAG GGTGCA GGCCTC TGATGG CAACAA CACCTC CTTCTG GTCTGA GGAGAT CAAGTT
TGACAC AGAGAT CCAGGC CTTCCT GCTGCC CCCTGT CTTCAA CATCCG CTCCCT GTCTGA CTCCTT
CCACAT CTACAT TGGCGC CCCCAA GCAGTC TGGCAA CACCCC TGTGAT CCAGGA CTACCC CCTGAT
CTATGA GATCAT CTTCTG GGAGAA CACCTC CAATGC TGAGAG GAAGAT CATTGA GAAGAA GACAGA
TGTGAC AGTGCC CAACCT GAAGCC CCTGAC AGTCTA CTGTGT GAAGGC CAGGGC TCACAC CATGGA
TGAGAA GCTGAA CAAGTC CTCTGT CTTCTC TGATGC TGTCTG TGAGAA GACCAA GCCTGG CTCCTT
CTCCAC CATCTG GATCAT CACCGG CCTGGG CGTGGT CTTCTT CTCTGT GATGGT GCTGTA TGCCCT
GAGGTC TGTCTG GAAGTA CCTGTG CCATGT CTGCTT CCCCCC CCTGAA ACCCCC CCGCTC CATTGA
TGAGTT CTTCTC TGAGCC CCCATC CAAGAA CCTGGT GCTGCT GACAGC TGAGGA GCACAC AGAGCG
CTGCTT CATCAT TGAGAA CACAGA CACAGT GGCTGT GGAGGT GAAGCA TGCCCC TGAGGA GGACCT
GAGGAA GTACTC CTCCCA GACCTC CCAAGA CTCTGG CAACTA CTCCAA TGAGGA GGAGGA GTCTGT
GGGCAC AGAGTC TGGCCA GGCTGT GCTGTC CAAGGC CCCATG TGGCGG CCCATG CTCTGT GCCATC
CCCCCC TGGCAC CCTGGA GGATGG CACCTG CTTCCT GGGCAA TGAGAA GTACCT GCAGTC CCCTGC
CCTGAG GACAGA GCCTGC CCTGCT GTGCTA AAACTC CTCAGG TGCAGG CTGCCT ATCAGA AGGTGG
TGGCTG GTGTGG CCAATG CCCTGG CTCACA AATACC ACTGAG ATCTTT TTCCCT CTGCCA AAAATT
ATGGGG ACATCA TGAAGC CCCTTG AGCATC TGACTT CTGGCT AATAAA GGAAAT TTATTT TCATTG
CAATAG TGTGTT GGAATT TTTTGT GTCTCT CACTCG GAAGGA CATATG GGAGGG CAAATC ATTTAA
AACATC AGAATG AGTATT TGGTTT AGAGTT TGGCAA CATATG CCCATA TGCTGG CTGCCA TGAACA
AAGGTT GGCTAT AAAGAG GTCATC AGTATA TGAAAC AGCCCC CTGCTG TCCATT CCTTAT TCCATA
GAAAAG CCTTGA CTTGAG GTTAGA TTTTTT TTATAT TTTGTT TTGTGT TATTTT TTTCTT TAACAT
CCCTAA AATTTT CCTTAC ATGTTT TACTAG CCAGAT TTTTCC TCCTCT CCTGAC TACTCC CAGTCA
TAGCTG TCCCTC TTCTCT TATGGA GATCCC TCGACC TGCAGA TGCGTT CACGGT GCACAG TCTCTG
CCGTCG GTCTCC TCAGCT TGTGTC TTGTGG GTAAGG GCTACT TCTCAG CACAGC CCTTAG AGGAGA
AAGCCT CTGTTT CTGTCA TCACAG AGAGCC CTGGTG TGGAGC AGCACA CTGATG TCCATA TCTGGA
GAACCC AGATCA GCACGG CCAGCA TCAGGC ACCCCA CGGGGG TCTTCC CCTTCA TTTTAG CTAAGC
CAGAAT AATATA GGCTAC AGCCAT ATTCAG GAAACG GCCTTG TTTATA ATTCAA AGGGTT GCGGCT
CTGCAC ACCCTG AATCTC ACGCCC GGTGGC GTTTAG AAGGTG GCCATC CCTTTA TCTCTT CCCATA
TAAACT AACTTG AAAAAT CCATCC CTACAC ATTGAT TTATAC TCTTCC TTTCTT
