# Supplementary figures and images for: Interferon stimulated immune profile changes in a humanized mouse model of HBV infection
Source: Nat Commun. 2023 Nov 15;14:7393. doi: 10.1038/s41467-023-43078-5 (PMC10652013; doi:10.1038/s41467-023-43078-5)

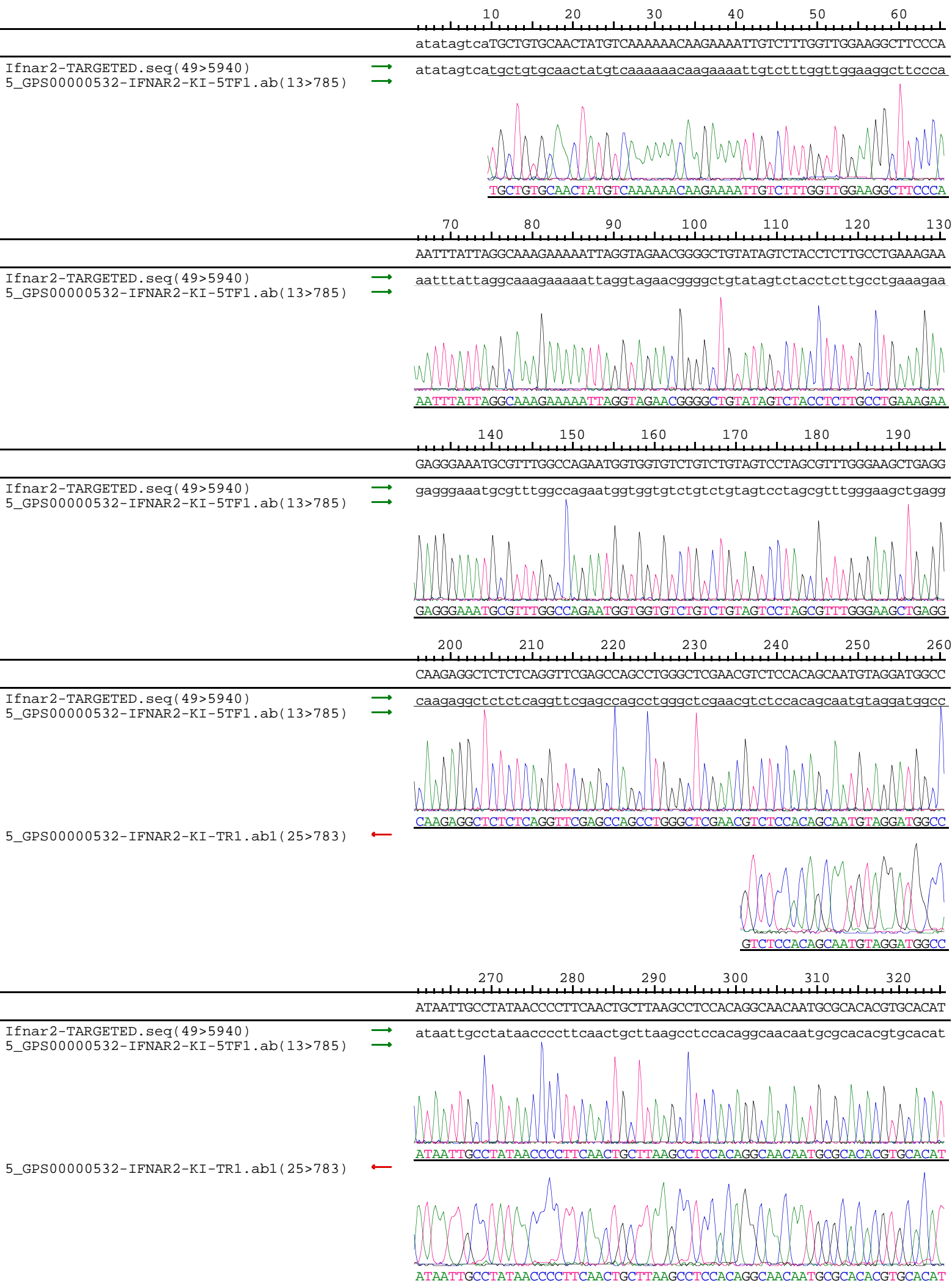

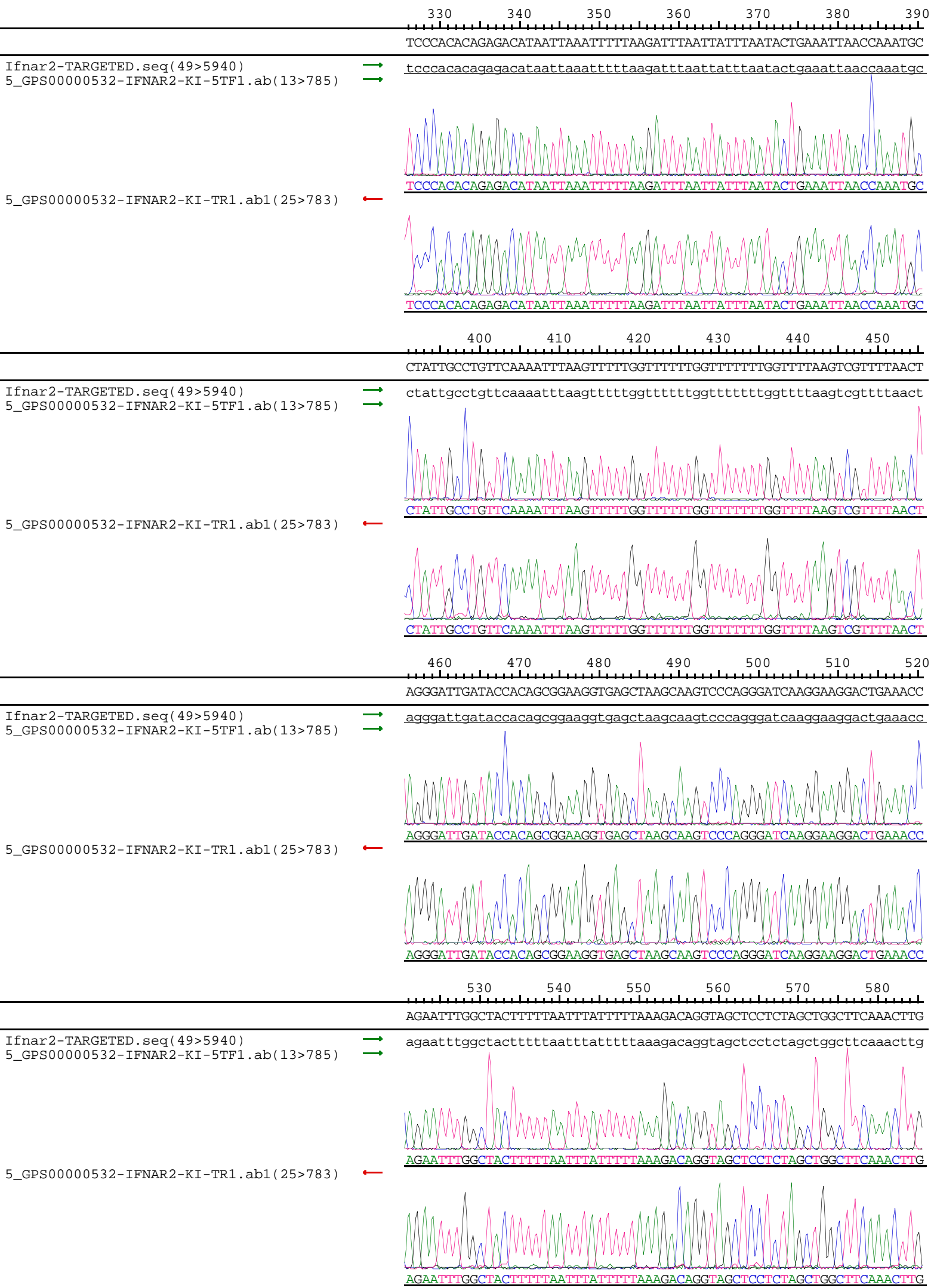

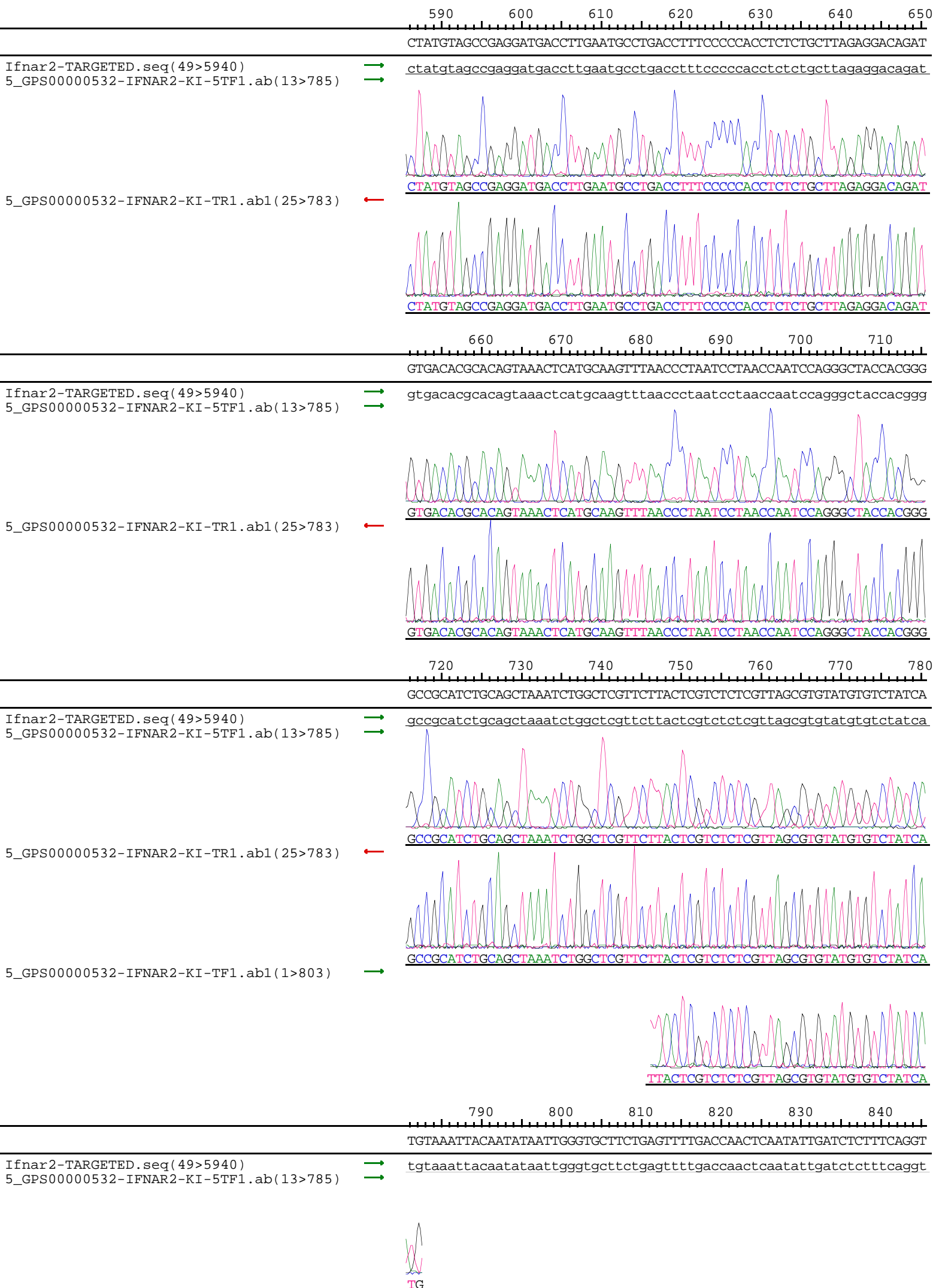

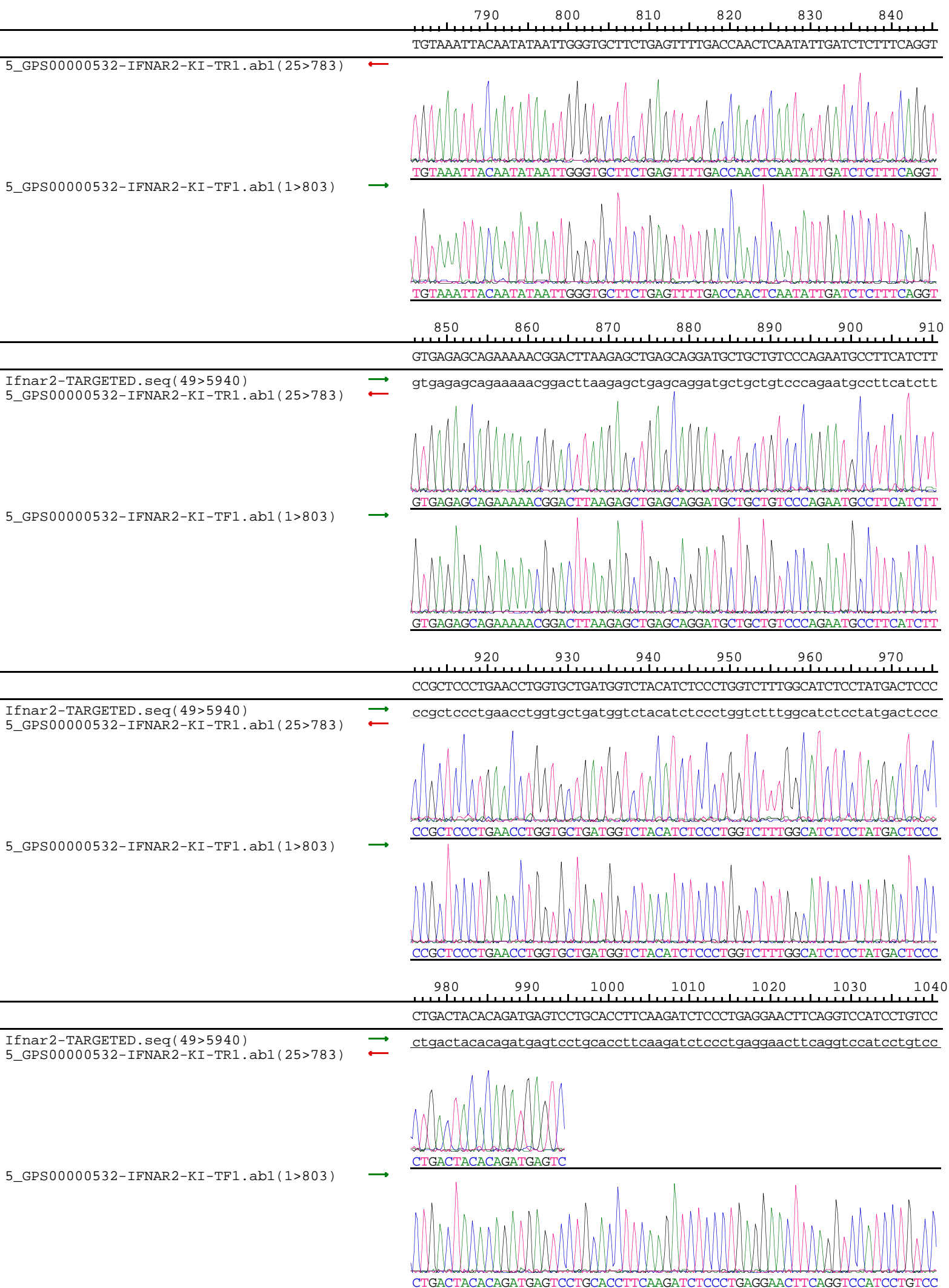

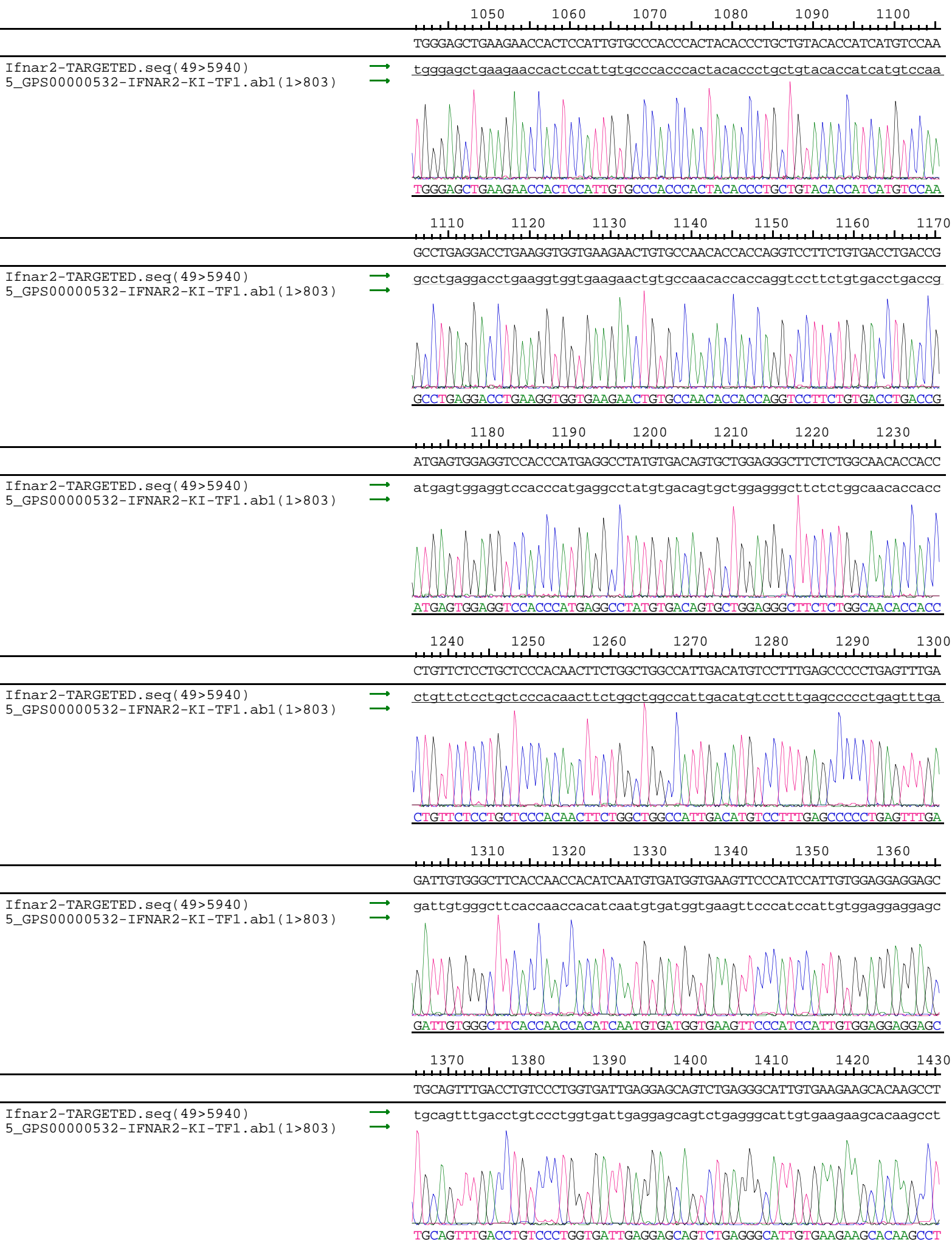

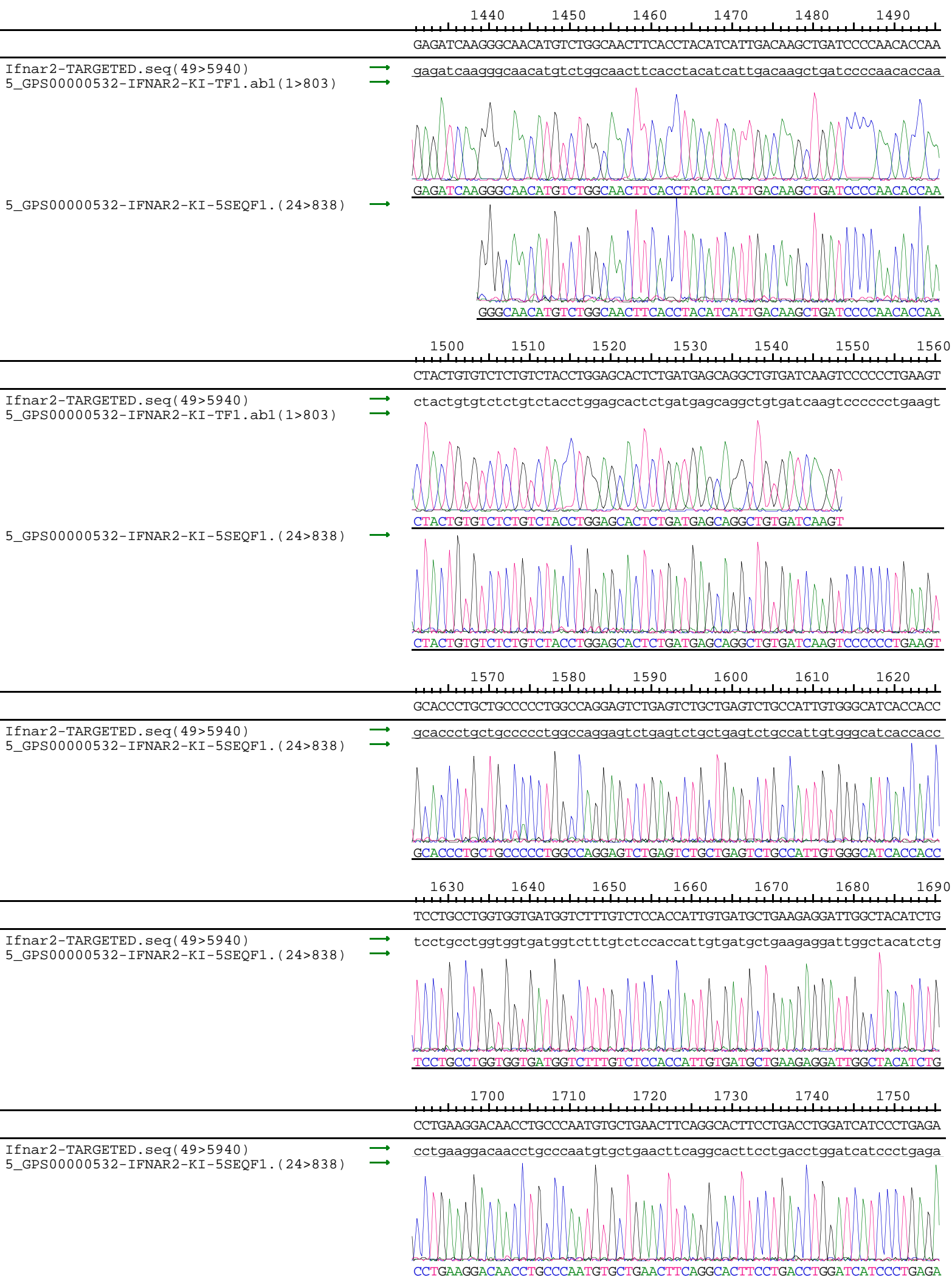

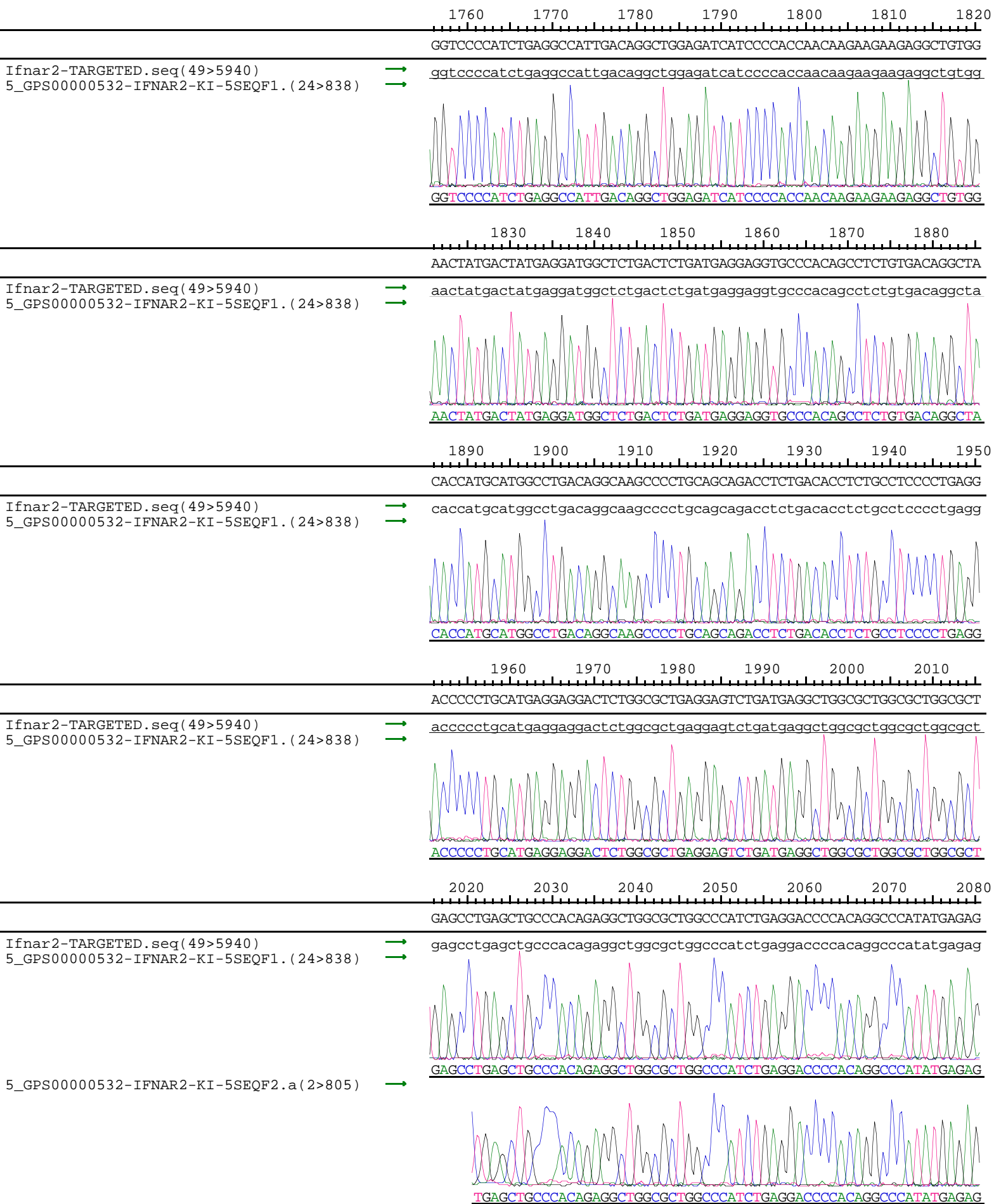

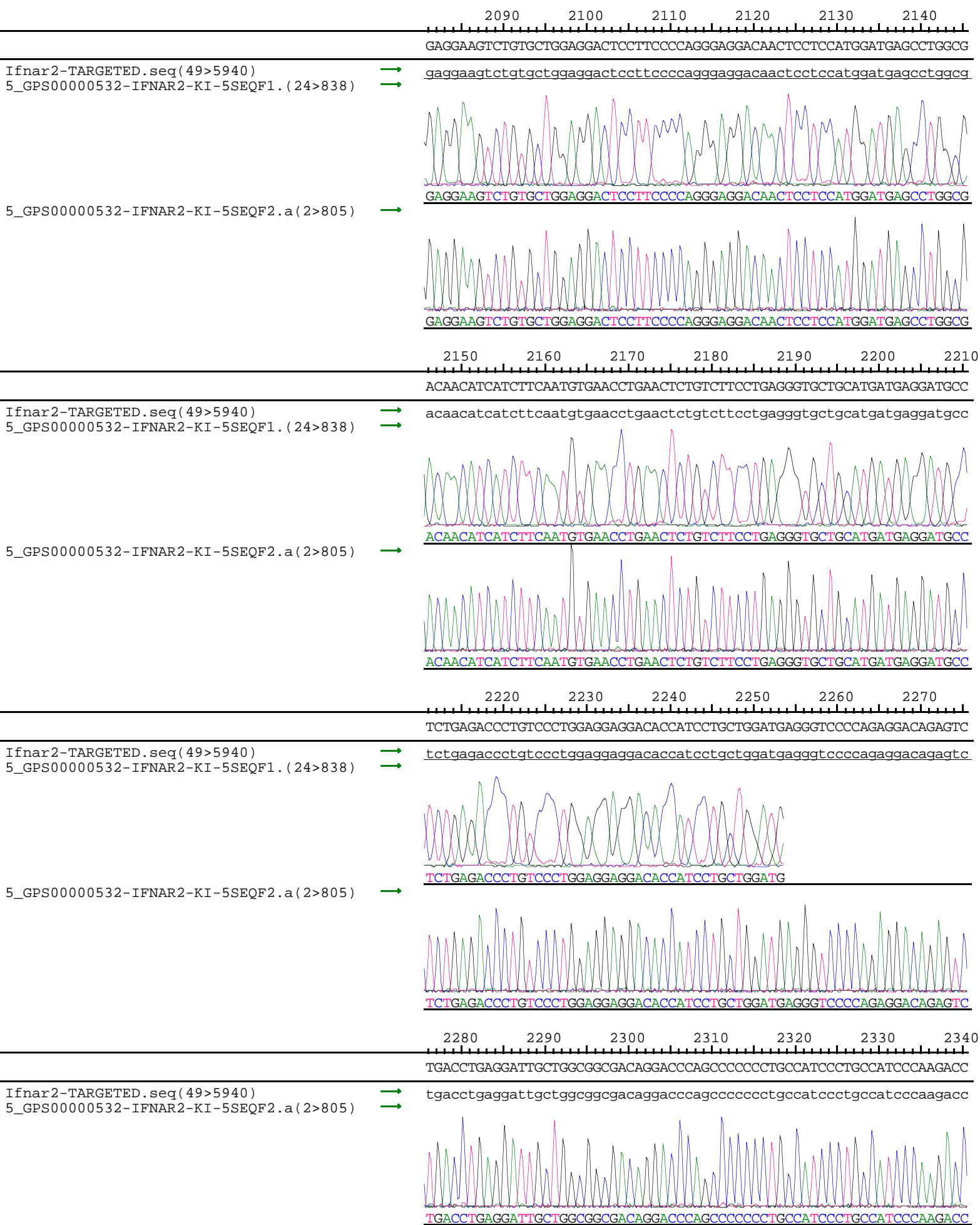

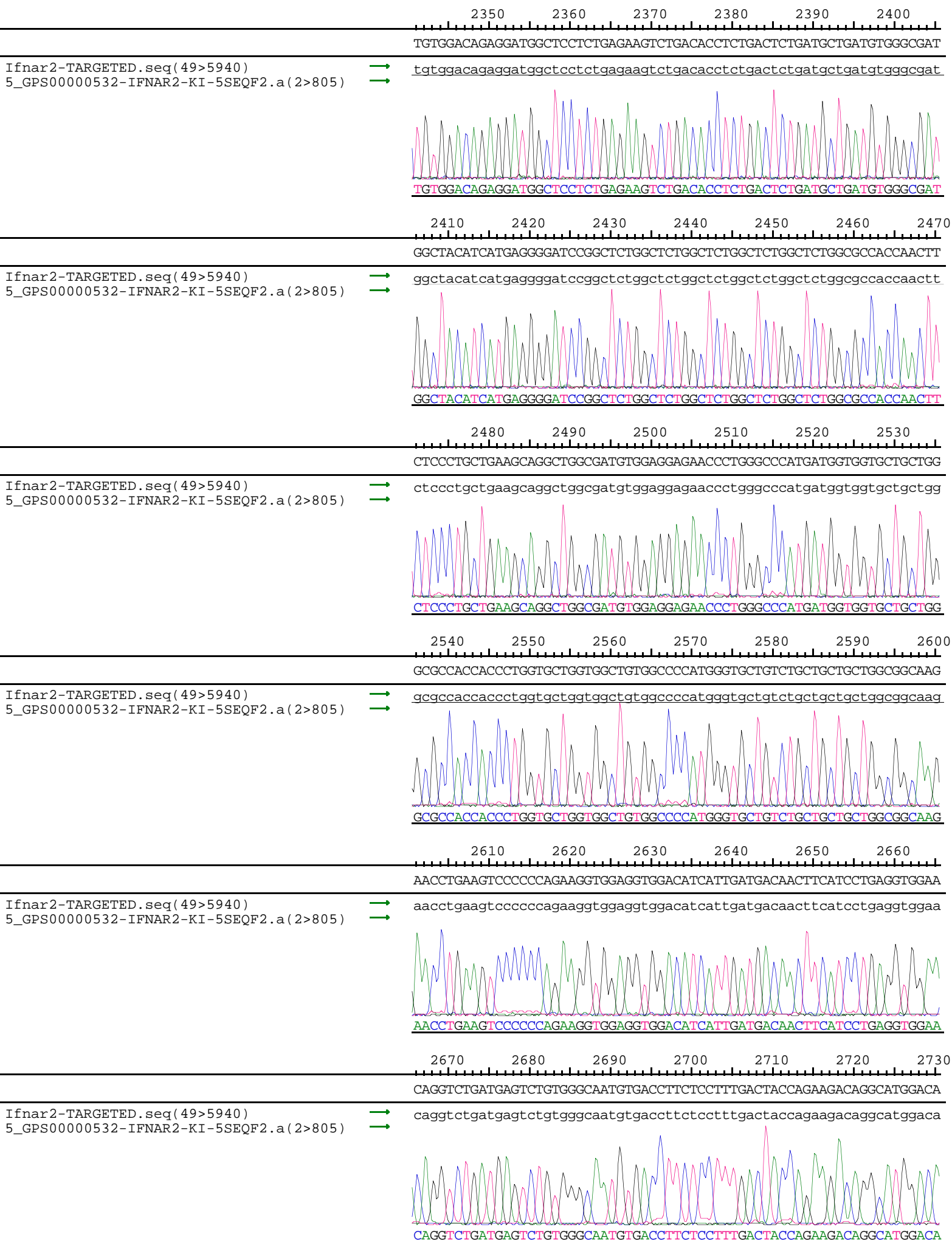

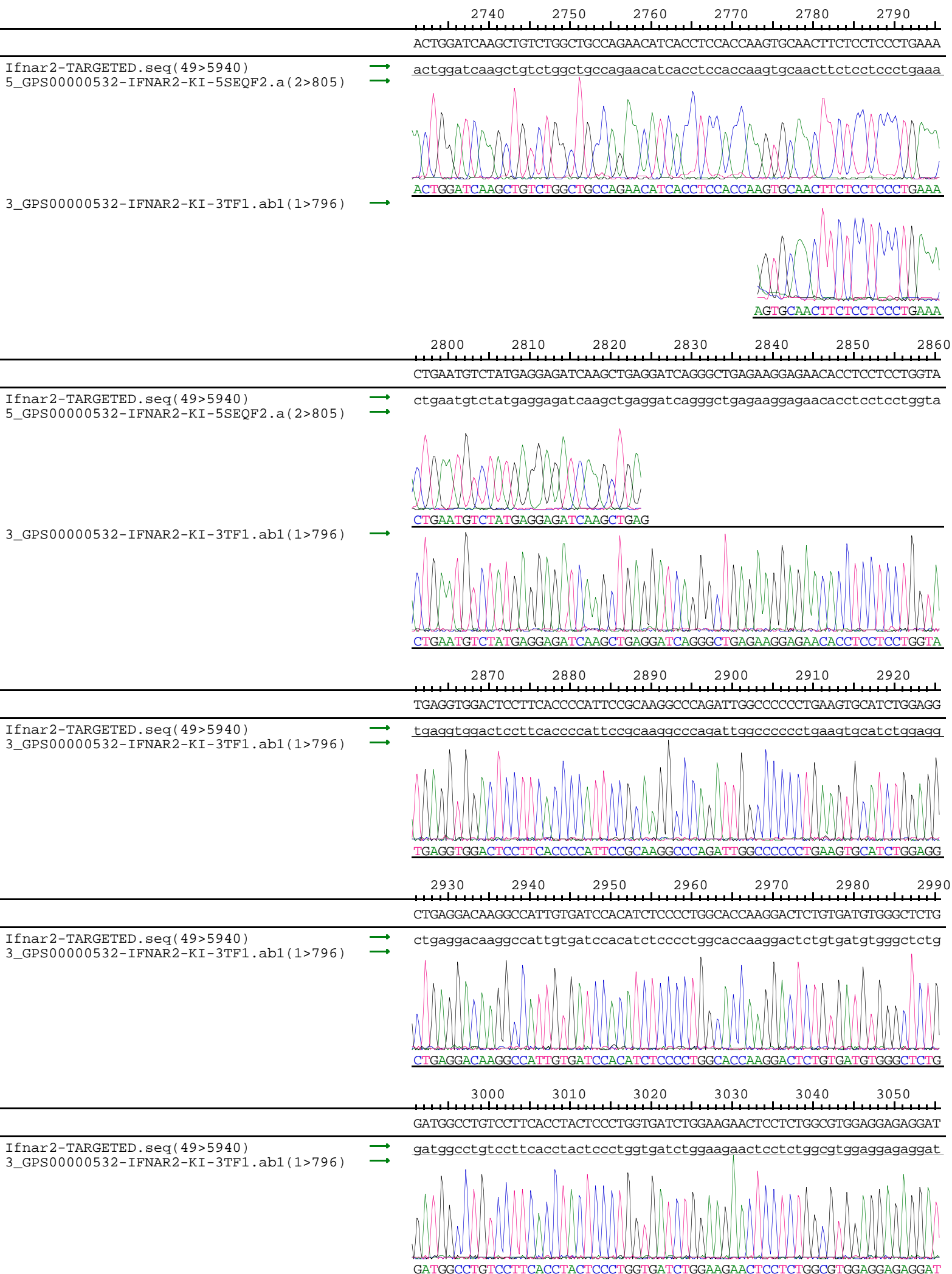

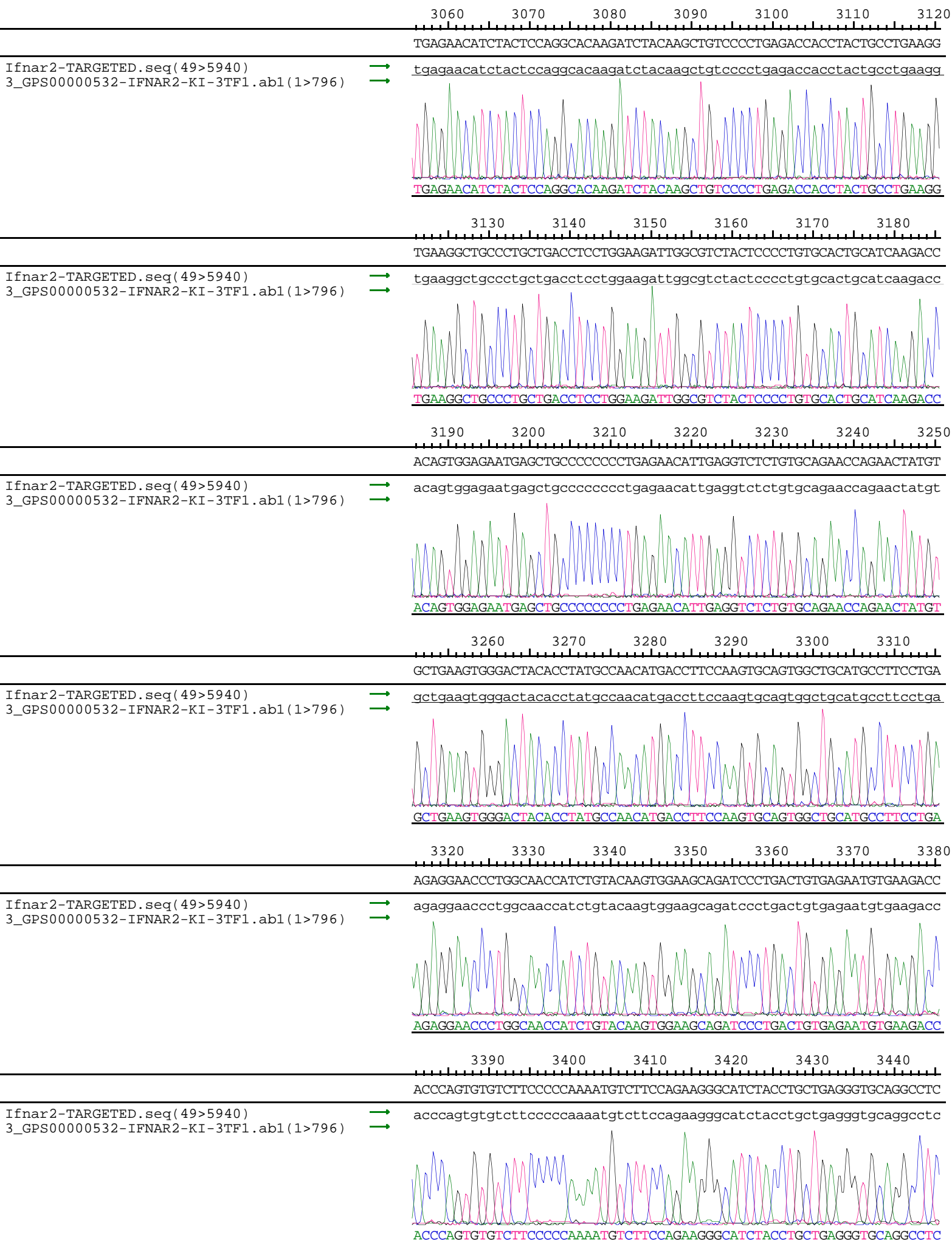

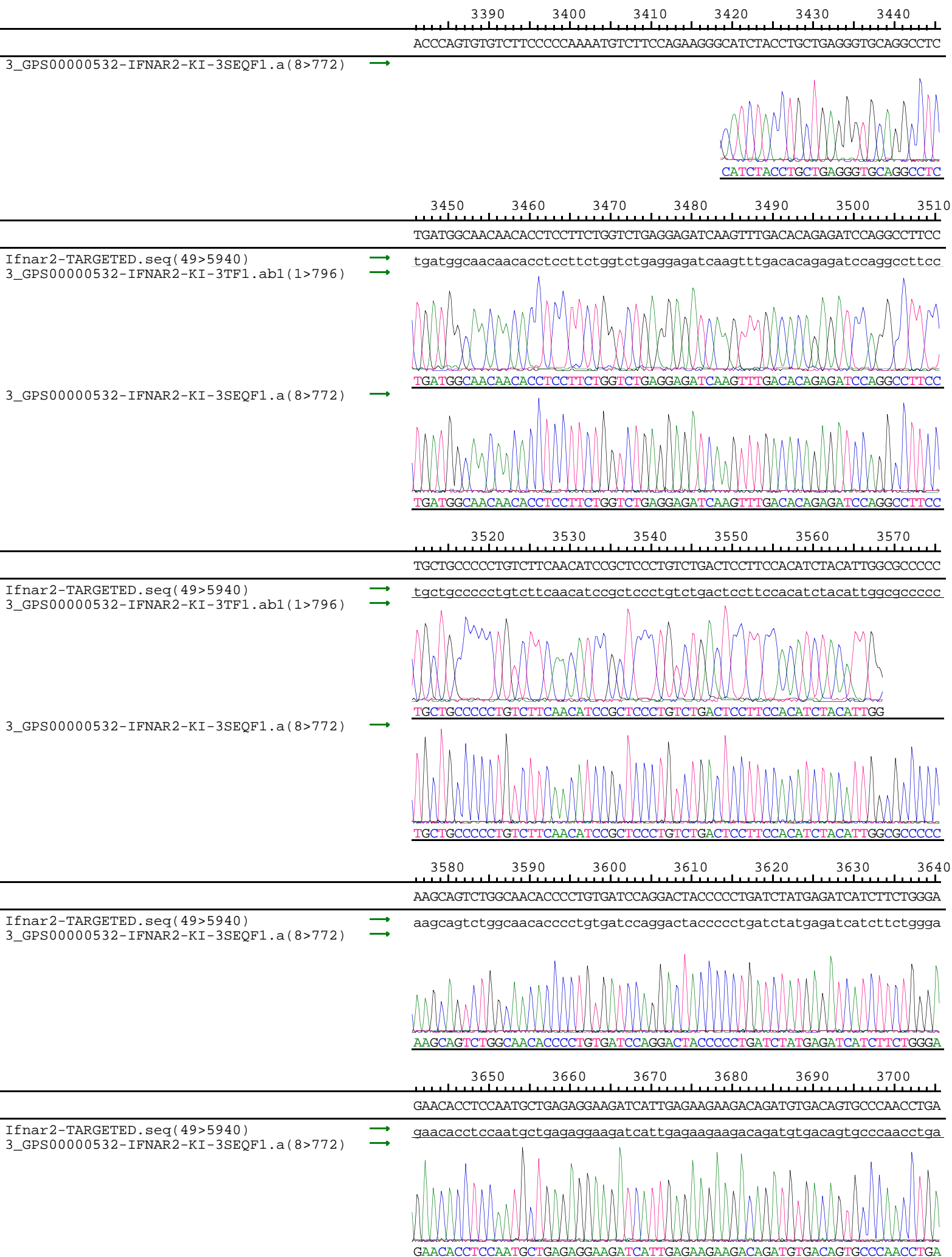

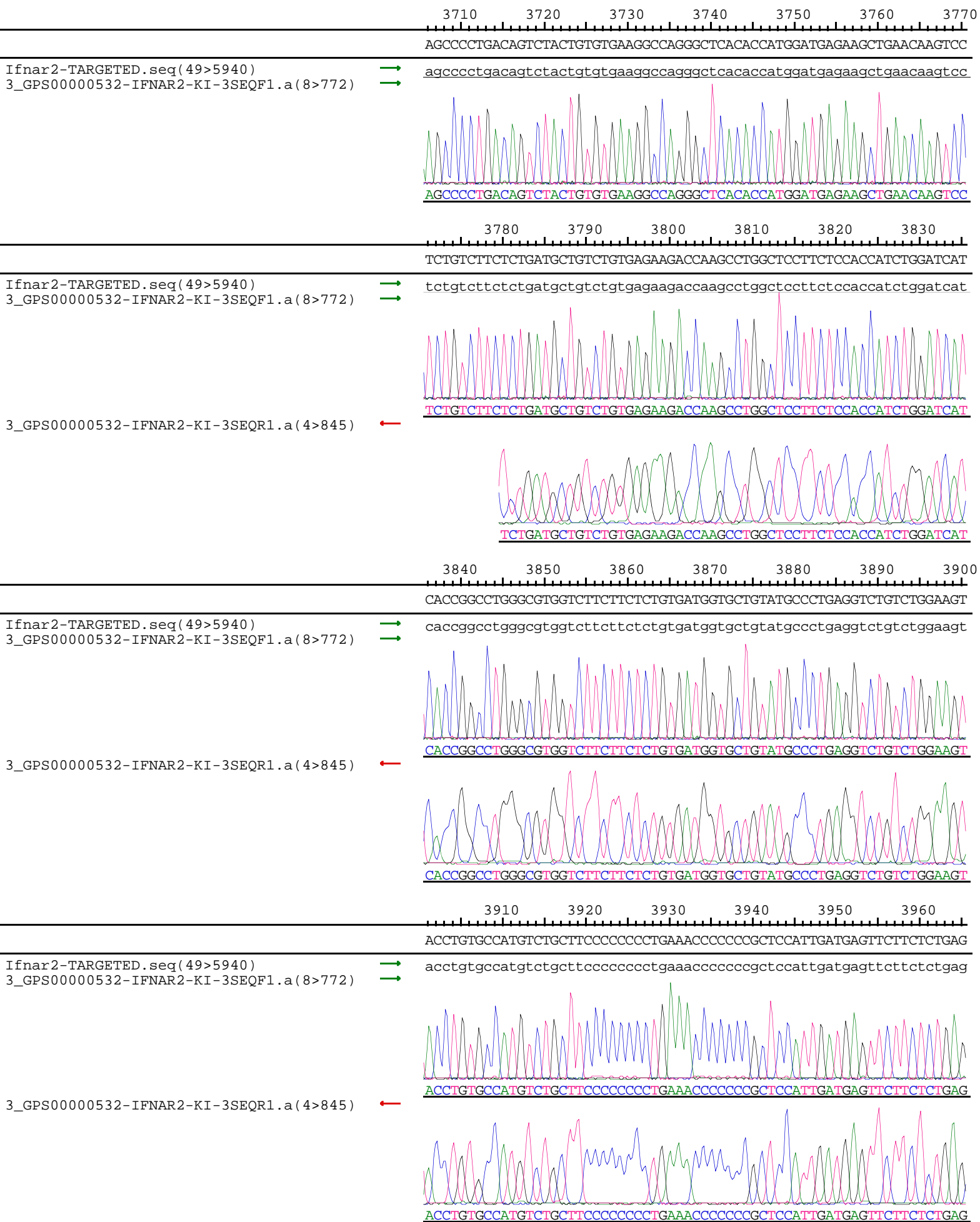

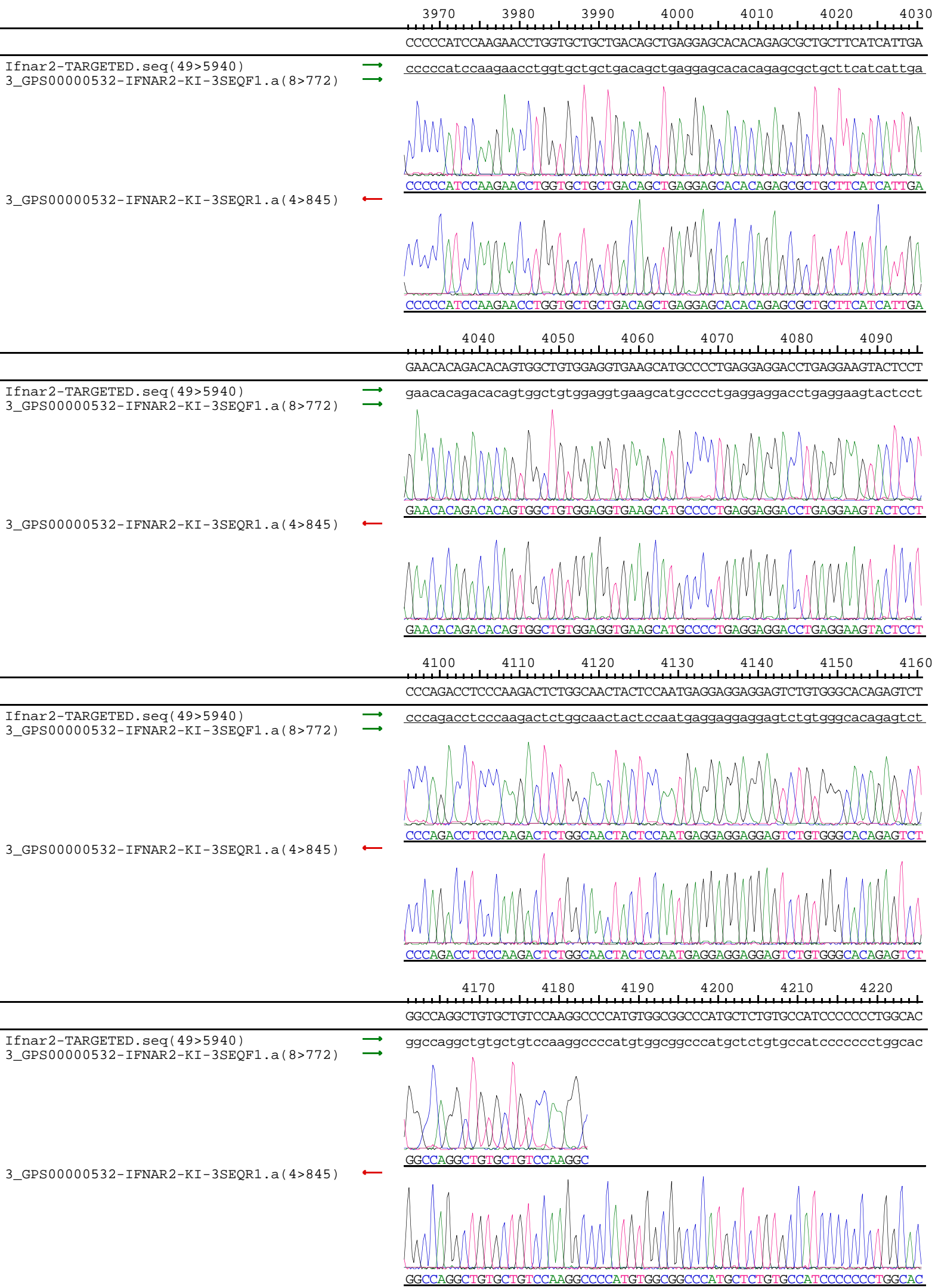

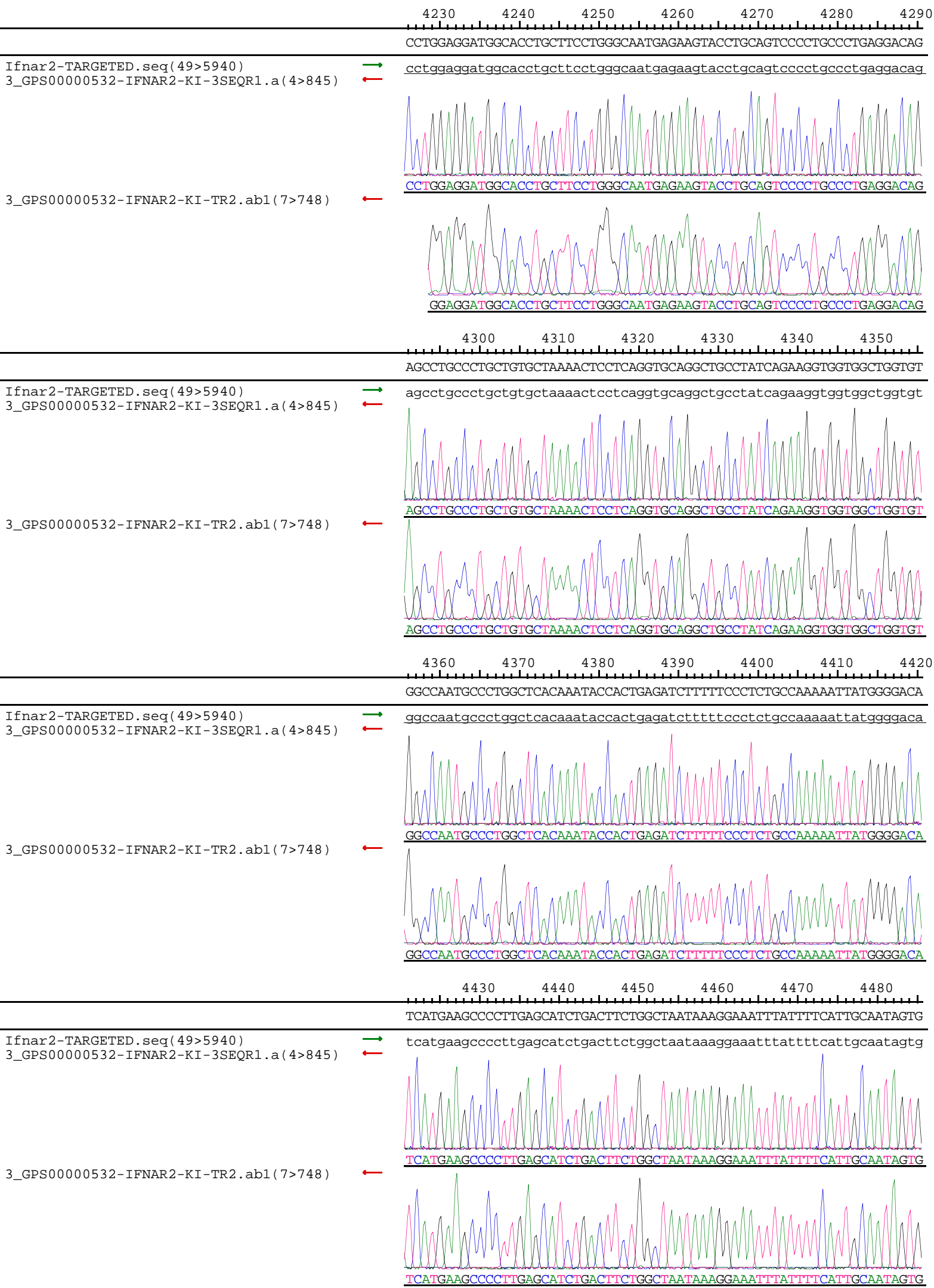

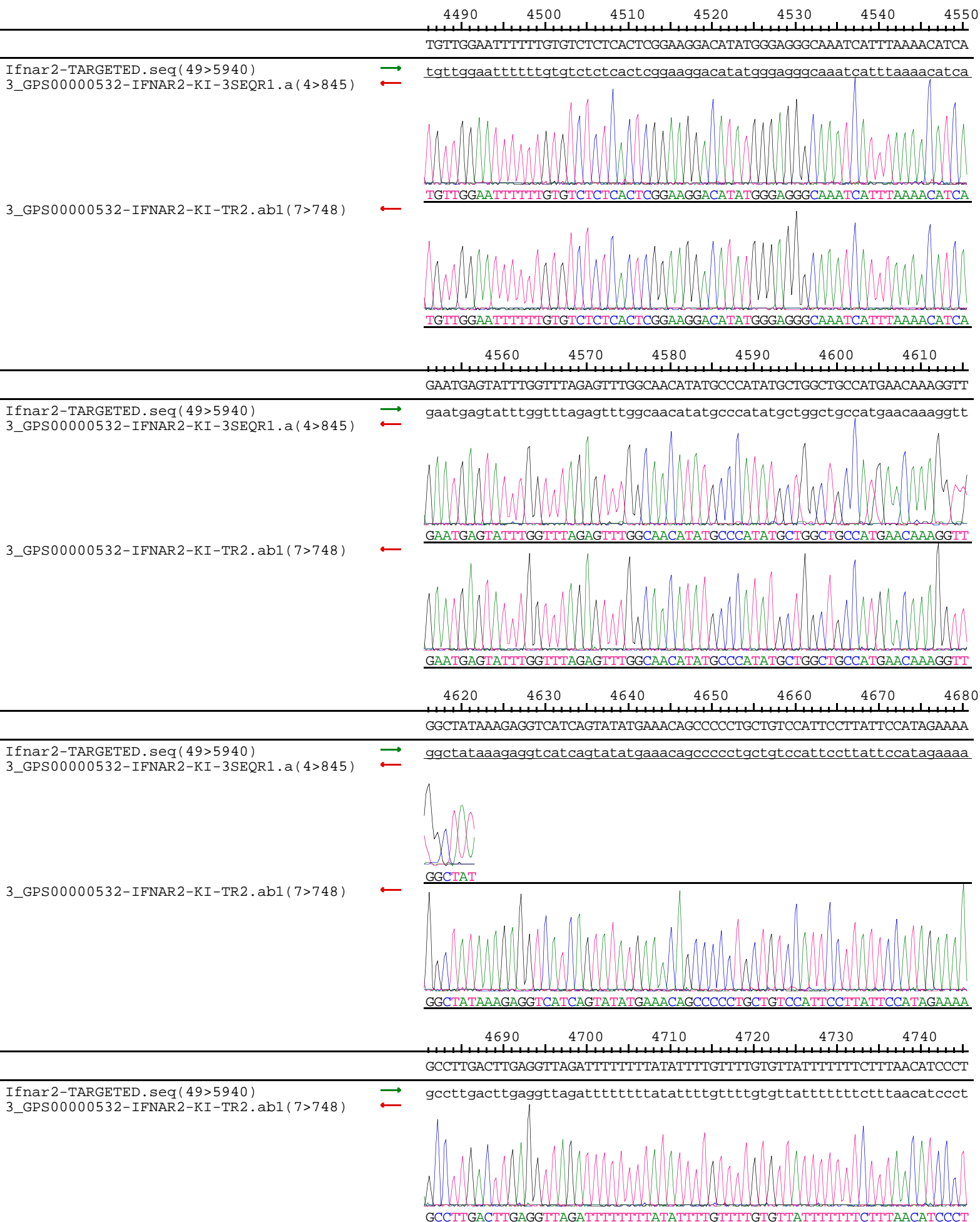

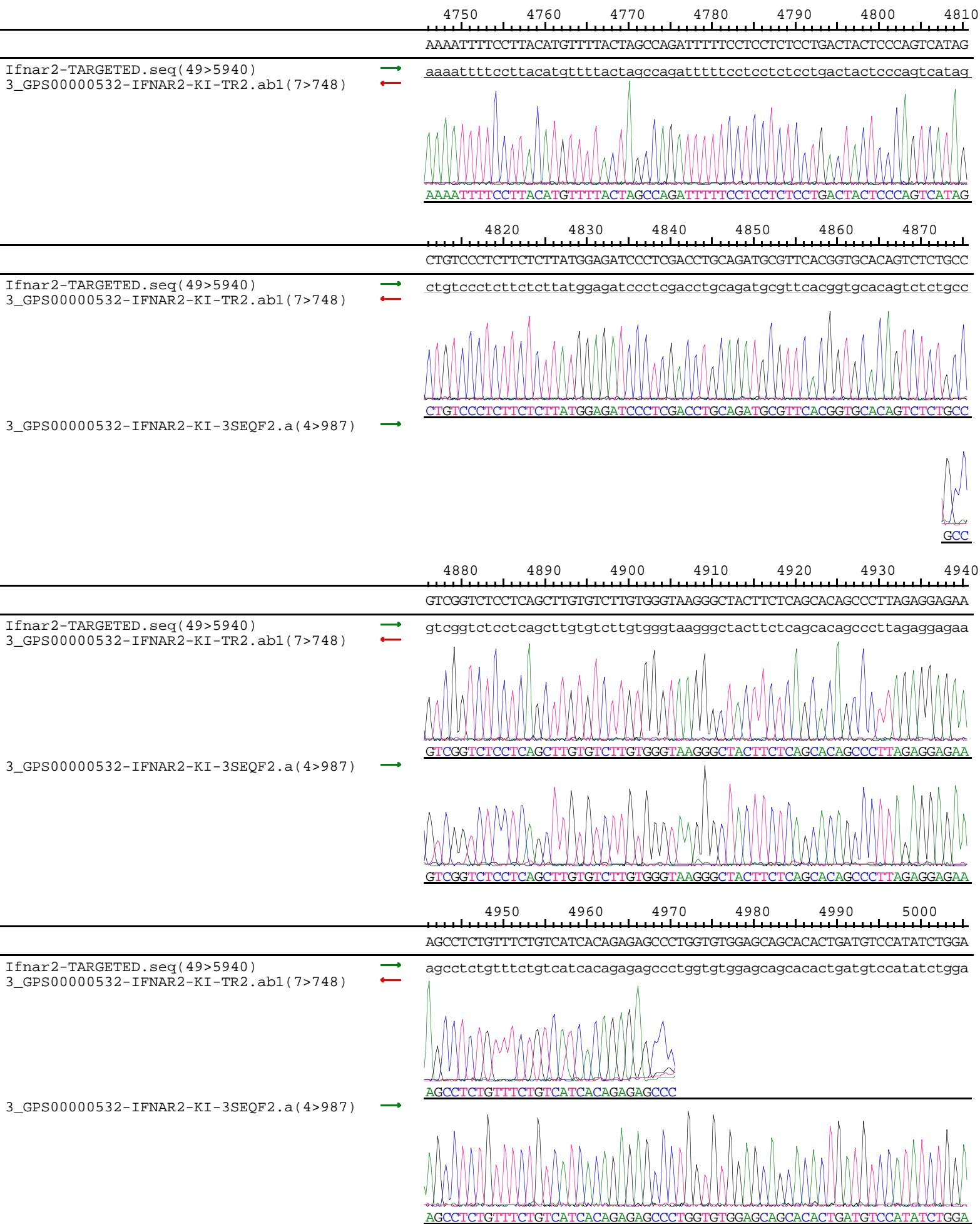

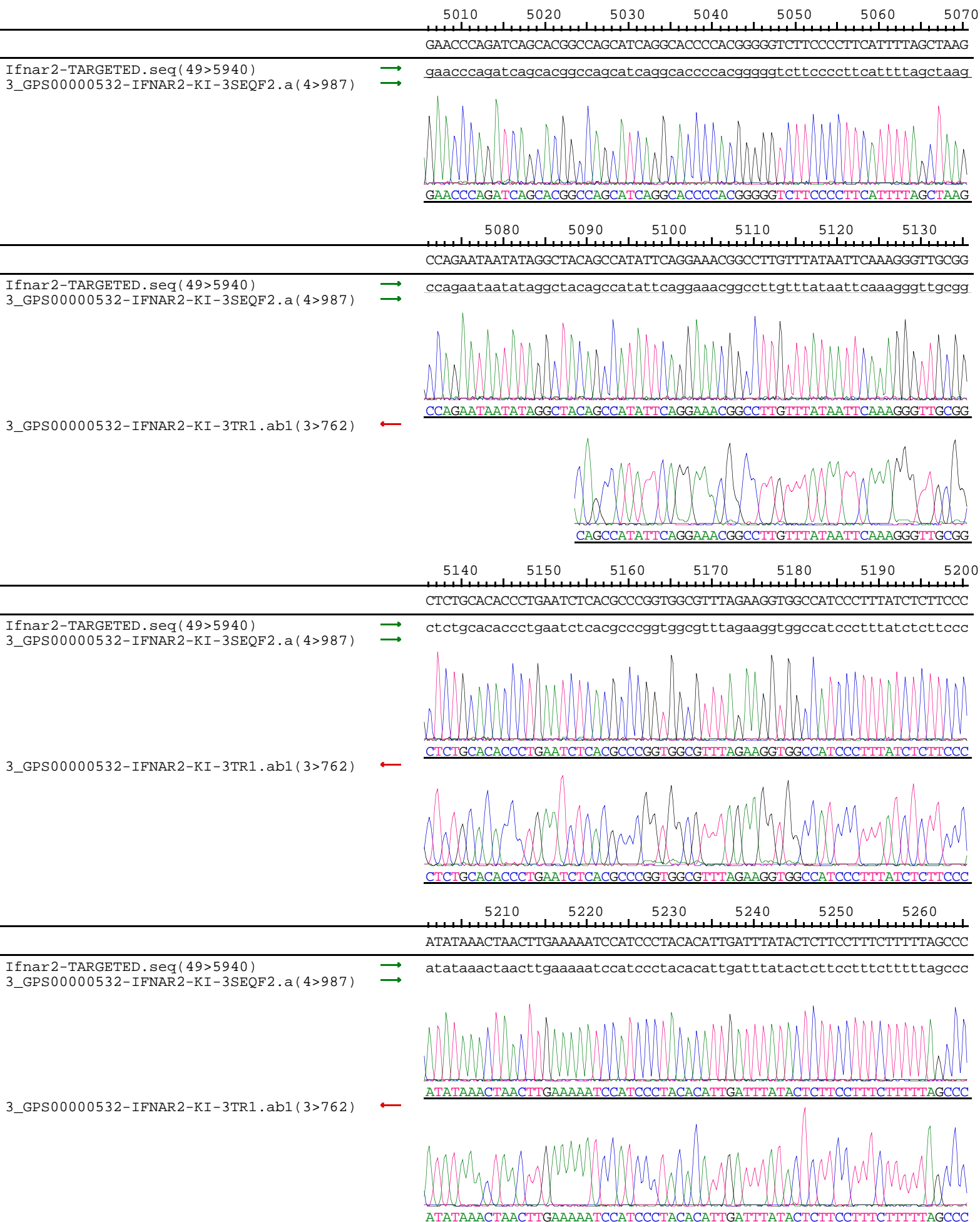

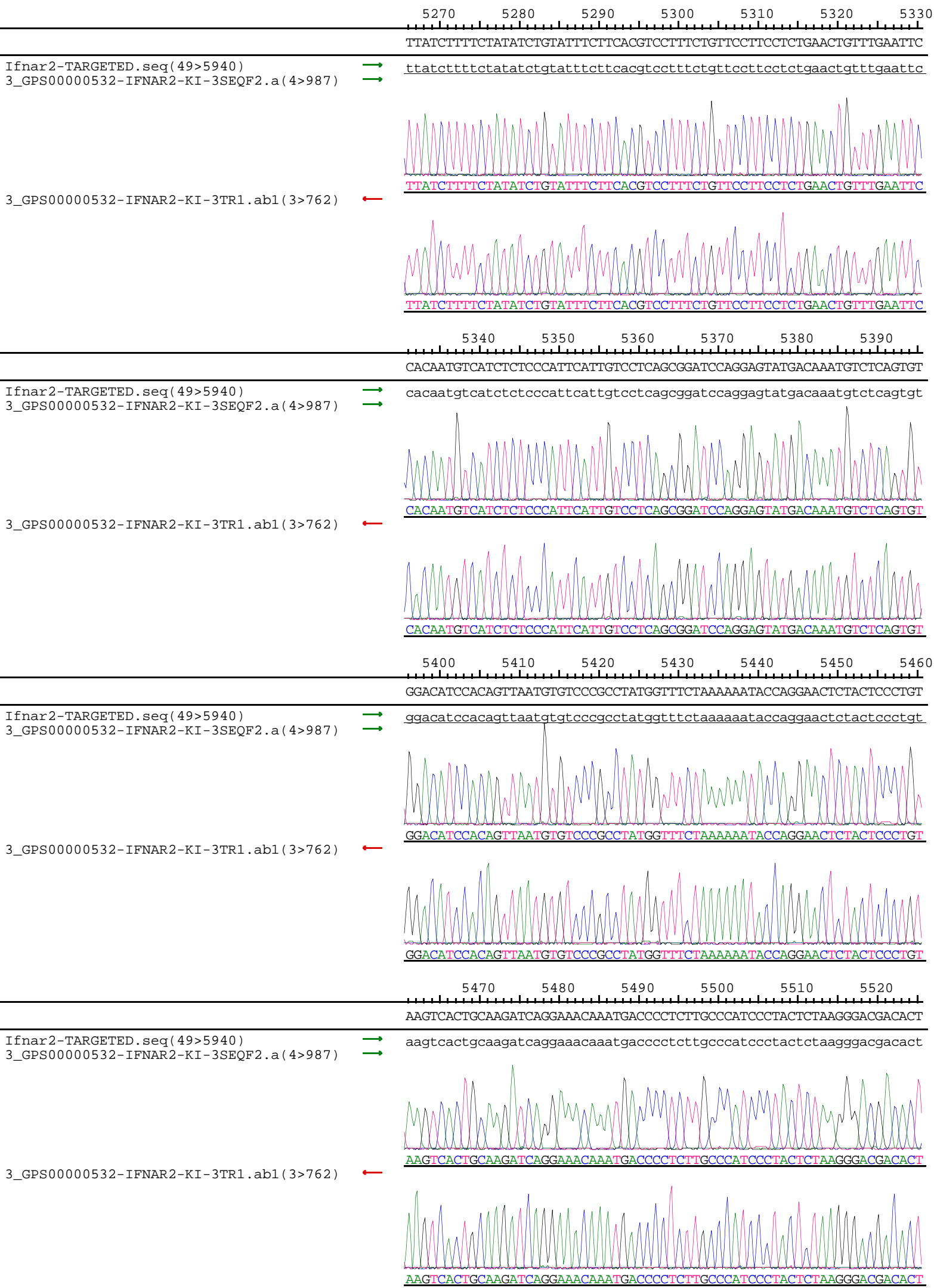

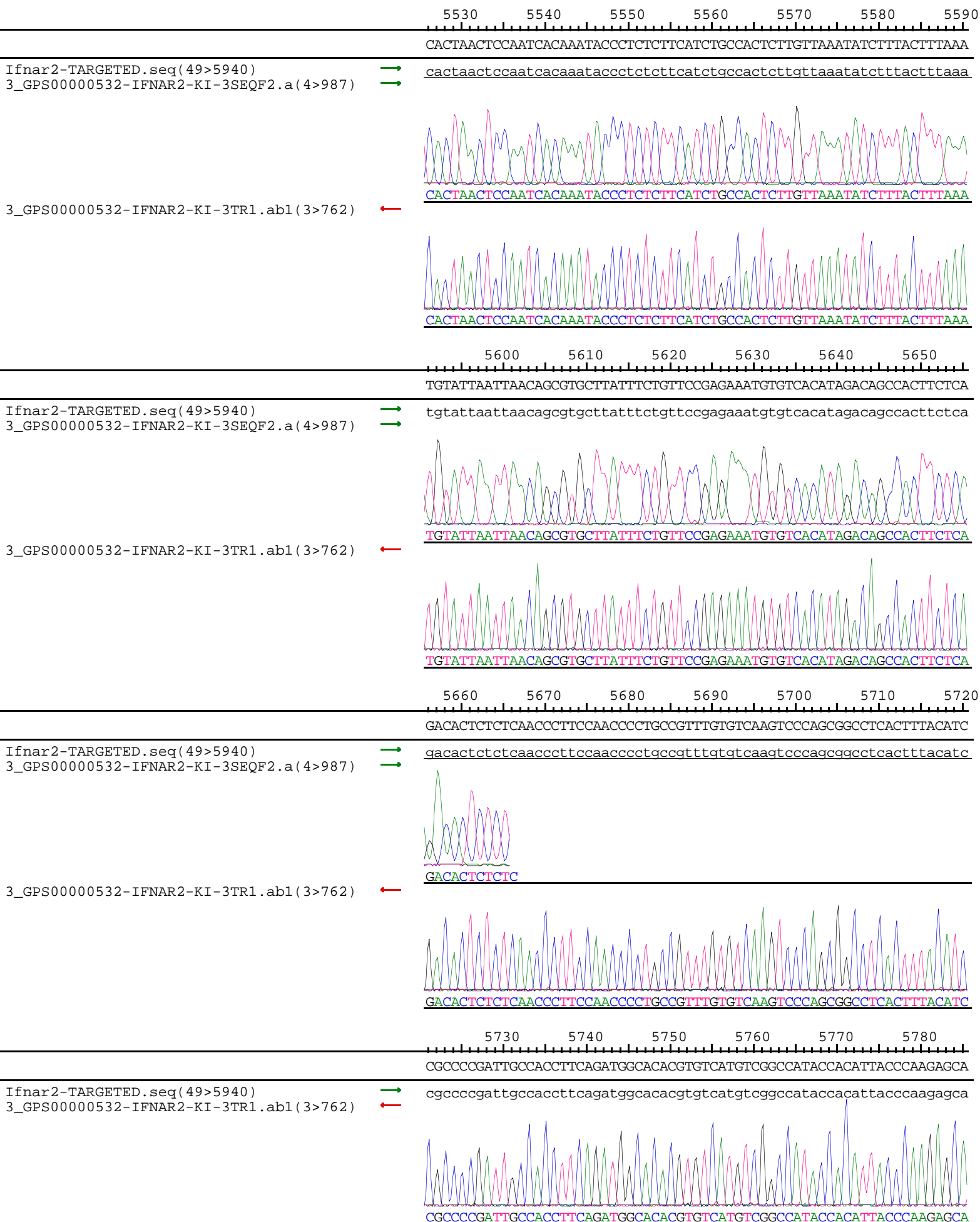

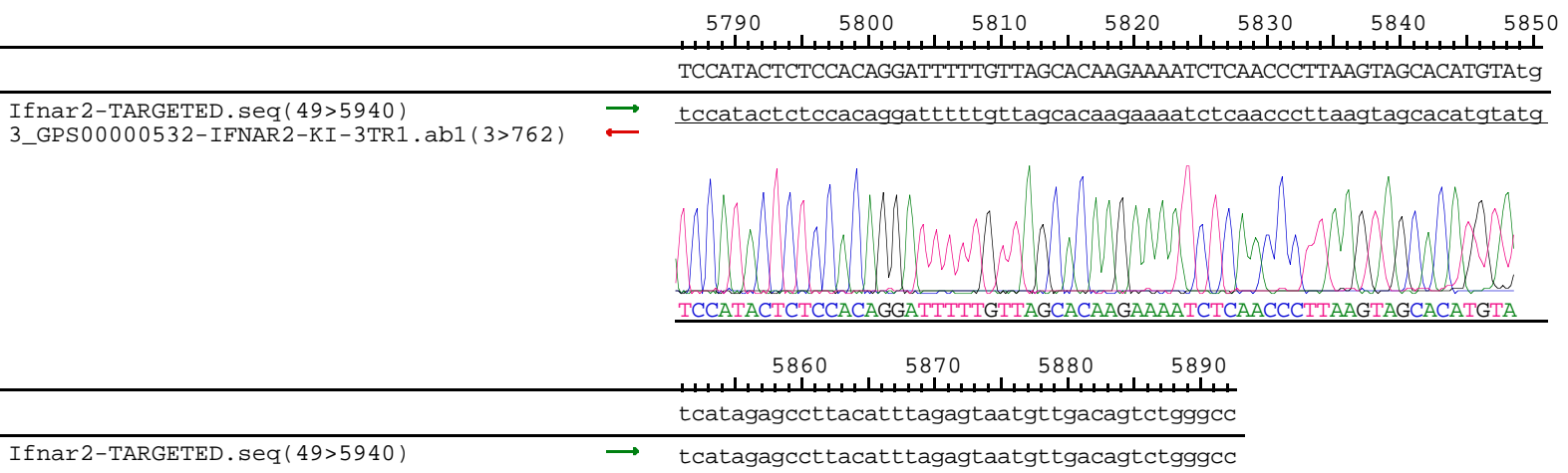

Supplement: Supplementary file 5 — Supplementary Data 2 [file 41467_2023_43078_MOESM5_ESM.pdf]
