# Supplementary material for: Interferon stimulated immune profile changes in a humanized mouse model of HBV infection
Source: Nat Commun. 2023 Nov 15;14:7393. doi: 10.1038/s41467-023-43078-5 (PMC10652013; doi:10.1038/s41467-023-43078-5)
Supplement: Supplementary file 7 — Reporting Summary [file 41467_2023_43078_MOESM7_ESM.pdf]

nature portfolio

Corresponding author(s): Feng Li

Last updated by author(s): Sep 27, 2023

Reporting Summary

Nature Portfolio wishes to improve the reproducibility of the work that we publish. This form provides structure for consistency and transparency in reporting. For further information on Nature Portfolio policies, see our Editorial Policies and the Editorial Policy Checklist.

Statistics

For all statistical analyses, confirm that the following items are present in the figure legend, table legend, main text, or Methods section.

n/a	Confirmed	
☐	☒	The exact sample size (*n*) for each experimental group/condition, given as a discrete number and unit of measurement
☐	☒	A statement on whether measurements were taken from distinct samples or whether the same sample was measured repeatedly
☐	☒	The statistical test(s) used AND whether they are one- or two-sided<br>*Only common tests should be described solely by name; describe more complex techniques in the Methods section.*
☒	☐	A description of all covariates tested
☐	☒	A description of any assumptions or corrections, such as tests of normality and adjustment for multiple comparisons
☐	☒	A full description of the statistical parameters including central tendency (e.g. means) or other basic estimates (e.g. regression coefficient) AND variation (e.g. standard deviation) or associated estimates of uncertainty (e.g. confidence intervals)
☐	☒	For null hypothesis testing, the test statistic (e.g. *F*, *t*, *r*) with confidence intervals, effect sizes, degrees of freedom and *P* value noted<br>*Give P values as exact values whenever suitable.*
☒	☐	For Bayesian analysis, information on the choice of priors and Markov chain Monte Carlo settings
☒	☐	For hierarchical and complex designs, identification of the appropriate level for tests and full reporting of outcomes
☒	☐	Estimates of effect sizes (e.g. Cohen's *d*, Pearson's *r*), indicating how they were calculated

Our web collection on statistics for biologists contains articles on many of the points above.

Software and code

Policy information about availability of computer code

Data collection	Flow cytometric data were collected using Cytek SpectroFlo(v1.0.0.0) software.
Data analysis	Statistical analysis and graphic representations were performed with GraphPad Prism(v8.0.1) software.<br>Bulk RNA-sequencing data processing and analysis were performed with fastp(v0.23.1), STAR(v2.7.9a), HISAT2(v2.2.1), featureCounts(v2.0.1), edgeR (v.3.32.1) packages, fgsea (v.1.16.0) packages of R studio.<br>The single-cell RNA-sequencing data were processed using an open-source pipeline [https://github.com/MGI-tech-bioinformatics/DNBelab_C_Series_HT_scRNA-analysis-software]<br>The flow cytometric data were analyzed using FlowJo software(v10.7.1).<br>Continuous variables were expressed as median (interquartile range, IQR). Categorical variables were summarized as the counts and percentages in each category. Unpaired t test, One-way ANOVA tests or Kruskal–Wallis tests were applied to continuous variables as appropriate; chi-square test or Fisher's exact test were applied to categorical variables as appropriate, log-rank (Mantel-Cox) test was applied to virus RNA clearance, p<0.05 was considered statistically significant.

For manuscripts utilizing custom algorithms or software that are central to the research but not yet described in published literature, software must be made available to editors and reviewers. We strongly encourage code deposition in a community repository (e.g. GitHub). See the Nature Portfolio guidelines for submitting code & software for further information.

Data

Policy information about availability of data

All manuscripts must include a data availability statement. This statement should provide the following information, where applicable:

- Accession codes, unique identifiers, or web links for publicly available datasets
- A description of any restrictions on data availability
- For clinical datasets or third party data, please ensure that the statement adheres to our policy

All tissue-specific bulk RNA-seq and single-cell RNA-seq data pivotal to this study have been responsibly deposited in the National Center for Biotechnology Information Gene Expression Omnibus and are accessible via the accession code GSE237519 [https://www.ncbi.nlm.nih.gov/geo/query/acc.cgi?acc=GSE237519]. Complementary bulk RNA-seq data can be accessed in the Genome Sequence Archive (GSA) or GSA-Human under the BioProject accession PRJCA017918 [https://ngdc.cncb.ac.cn/bioproject/browse/PRJCA017918]. Additionally, databases/datasets such as the mouse mm10 genome [https://www.ncbi.nlm.nih.gov/datasets/genome/GCF_000001635.20/], human hg38 genome [https://www.ncbi.nlm.nih.gov/datasets/genome/GCF_000001405.26/], and KEGG pathways [https://www.genome.jp/kegg/pathway.html] used in the study are mentioned along with the appropriately accessible links/accession codes in the manuscript under the "Data availability" section and in this reporting summary. Source data are also provided with this paper. The remaining data that support our findings are available within the Article, Supplementary Information, or Source Data file.

Research involving human participants, their data, or biological material

Policy information about studies with human participants or human data. See also policy information about sex, gender (identity/presentation), and sexual orientation and race, ethnicity and racism.

Reporting on sex and gender	Sex was not considered in the study design since all the previously published studies have reported that chronic hepatitis B virus (HBV) functional cure by interferon occurs in both men and women.
Reporting on race, ethnicity, or other socially relevant groupings	See above
Population characteristics	Blood samples were collected from a healthy donor: Female, 35 years old.
Recruitment	The donor was enrolled as a healthy control at Guangzhou Eighth People's Hospital, Guangzhou Medical University, with informed consent.
Ethics oversight	The collection and processing of all samples was approved by the Ethics Committee of the Guangzhou Eighth People's Hospital, Guangzhou Medical University, Guangzhou, China (No. 202001134 and 202115202)

Note that full information on the approval of the study protocol must also be provided in the manuscript.

Field-specific reporting

Please select the one below that is the best fit for your research. If you are not sure, read the appropriate sections before making your selection.

☒ Life sciences ☐ Behavioural & social sciences ☐ Ecological, evolutionary & environmental sciences

For a reference copy of the document with all sections, see nature.com/documents/nr-reporting-summary-flat.pdf

Life sciences study design

All studies must disclose on these points even when the disclosure is negative.

Sample size	The sample sizes for our experiments were determined based on established standards and conventions within the field of biology, which typically recommend a minimum sample size of three to ensure reproducibility and reliability of the findings. While no formal statistical calculation was performed to predetermine the sample size, our chosen sample sizes align with the accepted norms in the field and have been deemed sufficient in similar studies to detect biologically significant effects. This approach ensures that our study is consistent with, and comparable to, relevant research in the discipline.
Data exclusions	In both bulk and single-cell RNA sequencing, only the reads that successfully passed the quality controls were included in the analysis. No exclusions were made in this study, except for the samples allocated for the HBsAg test in the eighth week(Figure4b), which had to be omitted due to a shortage arising from hemolysis.
Replication	For clarification, the mouse model utilized three replicates for qPCR, as indicated in Fig. 1, all of which successfully replicated the results. The bulk RNA-seq involved three biological replicates for each tissue-specific analysis, as depicted in Figs. 2 and 3, with successful replication in each case. In the human Peg-IFNα2 treatment in HuIFNAR mice, each group included five or six biologically independent mice, and the results were consistently replicated. For single-cell RNA-seq, each sample was pooled from three mice, and each group had two successful replicates. Replication was verified in individual biological replicates and studies, as detailed in each figure legend. Consequently, all reported experimental data underwent successful replication, affirming the reliability of our findings.

Randomization	A healthy donor's peripheral blood mononuclear cells (PBMC) were selected at random for the in vitro tests to minimize selection bias. For other experimental groups, the selection was based on distinct criteria such as time-point status and genotype (huIFNAR vs. wildtype), making randomization inapplicable.
Blinding	No blinding of experimental groups was performed, as different genotypes (e.g., wildtype vs. huIFNAR) and different treatments (e.g., Mock vs. IFNα2) were directly compared for variations in molecular and cellular responses. Given the objective nature of the comparisons and the clear delineation between genotypes and treatments, blinding was deemed unnecessary for the integrity of the study.

Reporting for specific materials, systems and methods

We require information from authors about some types of materials, experimental systems and methods used in many studies. Here, indicate whether each material, system or method listed is relevant to your study. If you are not sure if a list item applies to your research, read the appropriate section before selecting a response.

Materials & experimental systems

n/a	Involved in the study
☐	☒ Antibodies
☒	☐ Eukaryotic cell lines
☒	☐ Palaeontology and archaeology
☐	☒ Animals and other organisms
☒	☐ Clinical data
☒	☐ Dual use research of concern
☒	☐ Plants

Methods

n/a	Involved in the study
☐	☐ ChIP-seq
☐	☒ Flow cytometry
☐	☐ MRI-based neuroimaging

Antibodies

Antibodies used

Marker	Fluorochrome	Manufacturer	Dilution (μl/10^6 cell /100 μl)	Clone	Catalog No.
NK1.1	APC	Biolegend	1.0	PK136	108710
B220	Percp-Cy5.5	Biolegend	0.5	RA3-6B2	103236
TCRγ/δ	BV421	Biolegend	2.5	GL3	118120
F4/80	PE-Dazzle594	Biolegend	3.0	BM8	123146
CD49b	PE/Cy7	Biolegend	0.4	HMα2	103518
CD8	BV570	Biolegend	0.5	53-6.7	100740
CD25	BV605	Biolegend	2.5	PC61	102036
CD19	Pacific Blue	Biolegend	0.5	6D5	115523
Ly6c	APC-Fire750	Biolegend	0.4	HK1.4	128046
CD3	Alexa Fluor 488	Biolegend	0.6	145-2C11	100321
PD1	PE	Biolegend	5.0	RMP1-14	114118
CD62L	BV785	Biolegend	0.5	MEL-14	104440
CXCR5	BV650	Biolegend	5.0	L138D7	145517
CD4	BV750	Biolegend	2.5	GK1.5	100467
CD11c	SB436	Thermo	2.5	N418	62-0114-82
MHCII	AF700	Biolegend	1.0	M5/114.15.2	107622
CD44	BV480	BD Biosciences	2.0	IM7	566200
CD11b	BV711	BD Biosciences	3.0	M1/70	563168
CD49a	Alexa Fluor 647	BD Biosciences	5.0	Ha31/8	562113
CD45	Alexa Fluor 532	Thermo	0.3	30-F11	58-0451-82
FVS575V	BV570	BD Biosciences	0.2	/	565694

Validation

APC anti-mouse NK-1.1 Antibody https://www.biolegend.com/en-us/products/apc-anti-mouse-nk-1-1-antibody-427?pdf=true&displayInline=true&leftRightMargin=15&topBottomMargin=15&filename=APC%20anti-mouse%20NK-1.1%20Antibody.pdf&v=20230714033116

PerCP/Cyanine5.5 anti-mouse/human CD45R/B220 Antibody https://www.biolegend.com/en-us/products/percp-cyanine5-5-anti-mouse-human-cd45r-b220-antibody-4267?pdf=true&displayInline=true&leftRightMargin=15&topBottomMargin=15&filename=PerCP/Cyanine5.5%20anti-mouse/human%20CD45R/B220%20Antibody.pdf&v=20230223043110

Brilliant Violet 421™ anti-mouse TCR γ/δ Antibody https://www.biolegend.com/en-us/products/brilliant-violet-421-anti-mouse-tcr-gamma-delta-antibody-7249?pdf=true&displayInline=true&leftRightMargin=15&topBottomMargin=15&filename=Brilliant%20Violet%20421%E2%84%A2%20anti-mouse%20TCR%20%CE%B3/%CE%B4%20Antibody.pdf&v=20230223043110

PE/Dazzle™ 594 anti-mouse F4/80 Antibody https://www.biolegend.com/en-us/products/pe-dazzle-594-anti-mouse-f4-80-antibody-10262?pdf=true&displayInline=true&leftRightMargin=15&topBottomMargin=15&filename=PE/Dazzle%E2%84%A2%20594%20anti-mouse%20F4/80%20Antibody.pdf&v=20230114013553

PE/Cyanine7 anti-mouse CD49b Antibody https://www.biolegend.com/en-us/products/pe-cyanine7-anti-mouse-cd49b-antibody-12083?pdf=true&displayInline=true&leftRightMargin=15&topBottomMargin=15&filename=PE/Cyanine7%20anti-mouse%

20CD49b%20Antibody.pdf&v=20230223043110

Brilliant Violet 570™ anti-mouse CD8a Antibody https://www.biolegend.com/en-us/products/brilliant-violet-570-anti-mouse-cd8a-antibody-7377?pdf=true&displayInline=true&leftRightMargin=15&topBottomMargin=15&filename=Brilliant%20Violet%20570%E2%84%A2%20anti-mouse%20CD8a%20Antibody.pdf&v=20230714033116

Brilliant Violet 605™ anti-mouse CD25 Antibody https://www.biolegend.com/en-us/products/brilliant-violet-605-anti-mouse-cd25-antibody-7639?pdf=true&displayInline=true&leftRightMargin=15&topBottomMargin=15&filename=Brilliant%20Violet%20605%E2%84%A2%20anti-mouse%20CD25%20Antibody.pdf&v=20230714033116

Pacific Blue™ anti-mouse CD19 Antibody https://www.biolegend.com/en-us/products/pacific-blue-anti-mouse-cd19-antibody-2987?pdf=true&displayInline=true&leftRightMargin=15&topBottomMargin=15&filename=Pacific%20Blue%E2%84%A2%20anti-mouse%20CD19%20Antibody.pdf&v=20230726063409

APC/Fire™ 750 anti-mouse Ly-6C Antibody https://www.biolegend.com/en-us/products/apc-fire-750-anti-mouse-ly-6c-antibody-13338?pdf=true&displayInline=true&leftRightMargin=15&topBottomMargin=15&filename=APC/Fire%E2%84%A2%20750%20anti-mouse%20Ly-6C%20Antibody.pdf&v=20230803061558

Alexa Fluor® 488 anti-mouse CD3ε Antibody https://www.biolegend.com/en-us/products/alexa-fluor-488-anti-mouse-cd3epsilon-antibody-2676?pdf=true&displayInline=true&leftRightMargin=15&topBottomMargin=15&filename=Alexa%20Fluor%C2%AE%20488%20anti-mouse%20CD3%CE%B5%20Antibody.pdf&v=20230726063409

PE anti-mouse CD279 (PD-1) Antibody https://www.biolegend.com/en-us/products/pe-anti-mouse-cd279-pd-1-antibody-12737?pdf=true&displayInline=true&leftRightMargin=15&topBottomMargin=15&filename=PE%20anti-mouse%20CD279%20(PD-1)%20Antibody.pdf&v=20230114013553

Brilliant Violet 785™ anti-mouse CD62L Antibody https://www.biolegend.com/en-us/products/brilliant-violet-785-anti-mouse-cd62l-antibody-13525?pdf=true&displayInline=true&leftRightMargin=15&topBottomMargin=15&filename=Brilliant%20Violet%20785%E2%84%A2%20anti-mouse%20CD62L%20Antibody.pdf&v=20230803063053

Brilliant Violet 650™ anti-mouse CD185 (CXCR5) Antibody https://www.biolegend.com/en-us/products/brilliant-violet-650-anti-mouse-cd185-cxcr5-antibody-8809?pdf=true&displayInline=true&leftRightMargin=15&topBottomMargin=15&filename=Brilliant%20Violet%20650%E2%84%A2%20anti-mouse%20CD185%20(CXCR5)%20Antibody.pdf&v=20230803063053

Brilliant Violet 750™ anti-mouse CD4 Antibody https://www.biolegend.com/en-us/products/brilliant-violet-750-anti-mouse-cd4-antibody-15756?pdf=true&displayInline=true&leftRightMargin=15&topBottomMargin=15&filename=Brilliant%20Violet%20750%E2%84%A2%20anti-mouse%20CD4%20Antibody.pdf&v=20230804063012

CD11c Monoclonal Antibody (N418), Super Bright™ 436, eBioscience™ https://www.thermofisher.cn/order/genome-database/dataSheetPdf?producttype=antibody&productsubtype=antibody_primary&productId=62-0114-82&version=345

Alexa Fluor® 700 anti-mouse I-A/I-E Antibody https://www.biolegend.com/en-us/products/alexa-fluor-700-anti-mouse-i-a-i-e-antibody-3413?pdf=true&displayInline=true&leftRightMargin=15&topBottomMargin=15&filename=Alexa%20Fluor%C2%AE%20700%20anti-mouse%20I-A/I-E%20Antibody.pdf&v=20230114013553

BD Horizon™ BV480 Rat Anti-Mouse CD44 https://www.bdbiosciences.com/content/dam/bdb/products/global/reagents/flow-cytometry-reagents/research-reagents/single-color-antibodies-ruo/566200_base/pdf/566200.pdf

BD Horizon™ BV711 Rat Anti-CD11b https://www.bdbiosciences.com/content/dam/bdb/products/global/reagents/flow-cytometry-reagents/research-reagents/single-color-antibodies-ruo/563168_base/pdf/563168.pdf

BD Pharmingen™ Alexa Fluor® 647 Hamster Anti-Rat/Mouse CD49a https://www.bdbiosciences.com/content/dam/bdb/products/global/reagents/flow-cytometry-reagents/research-reagents/single-color-antibodies-ruo/562113_base/pdf/562113.pdf

CD45 Monoclonal Antibody (30-F11), Alexa Fluor™ 532, eBioscience™ https://www.thermofisher.cn/order/genome-database/dataSheetPdf?producttype=antibody&productsubtype=antibody_primary&productId=58-0451-82&version=345

BD Horizon™ Fixable Viability Stain 575V https://www.bdbiosciences.com/content/dam/bdb/products/global/reagents/flow-cytometry-reagents/research-reagents/single-color-antibodies-ruo/565694_base/pdf/565694.pdf

Animals and other research organisms

Policy information about studies involving animals; ARRIVE guidelines recommended for reporting animal research, and Sex and Gender in Research

Laboratory animals

The huIFNAR mice were established as illustrated in figure 1A. Female C57BL6 mice (4–10 weeks of age) were super-ovulated by intraperitoneal injection with 5 IU Pregnant Mare Serum Gonadotropin (PMSG), followed by a 5 IU Human Chorionic Gonadotropin (HCG) at 48 hours later. Experienced male C57BL6 mice (5 months of age) were mated with the superovulation female. 16 hours later. Plugged females were sacrificed 14-16 hours following mating. Oviducts were collected, and oocyte-cumulus complexes were released from the oviducts. Fertilized embryos with visible pronuclei were selected for pronuclear microinjection and transferred to microinjection dishes containing M2 medium under mineral oil. The CRISPR/Cas9, sgRNA and linearized huIFNAR DNA mixture was prepared and introduced into the pronuclei of fertilized embryos by microinjection using a continuous flow injection mode.
In addition to the establishment of the huIFNAR mice as illustrated in Figure 1A, we would like to provide further information on the housing conditions for the mice. The mice were housed in a controlled environment with a 12/12-hour light/dark cycle. The ambient

	temperature was maintained at 22±2°C, and the relative humidity was kept between 40% and 60%. The mice were kept in standard laboratory cages with ad libitum access to food and water.
Wild animals	No wild animals were used in the study.
Reporting on sex	Sex was not considered in the study design since all the previously published studies have reported that chronic hepatitis B virus (HBV) functional cure by interferon occurs in both men and women.
Field-collected samples	No field-collected samples were used in this study.
Ethics oversight	All animal experiments were conducted following Chinese guidelines for housing and care of laboratory animals and per protocols approved by the Institutional Animal Care and Use Committee (No. 2016-153) within the Guangdong Province Academy of Agricultural Sciences Animal Hygiene Institute. And all mouse experiments followed the guidelines developed by the National Centre for the Replacement, Refinement and Reduction of Animals in Research (NC3Rs).

Note that full information on the approval of the study protocol must also be provided in the manuscript.

Plants

Seed stocks	*Report on the source of all seed stocks or other plant material used. If applicable, state the seed stock centre and catalogue number. If plant specimens were collected from the field, describe the collection location, date and sampling procedures.*
Novel plant genotypes	*Describe the methods by which all novel plant genotypes were produced. This includes those generated by transgenic approaches, gene editing, chemical/radiation-based mutagenesis and hybridization. For transgenic lines, describe the transformation method, the number of independent lines analyzed and the generation upon which experiments were performed. For gene-edited lines, describe the editor used, the endogenous sequence targeted for editing, the targeting guide RNA sequence (if applicable) and how the editor was applied.*
Authentication	*Describe any authentication procedures for each seed stock used or novel genotype generated. Describe any experiments used to assess the effect of a mutation and, where applicable, how potential secondary effects (e.g. second site T-DNA insertions, mosiacism, off-target gene editing) were examined.*

ChIP-seq

Data deposition

☐ Confirm that both raw and final processed data have been deposited in a public database such as GEO.

☐ Confirm that you have deposited or provided access to graph files (e.g. BED files) for the called peaks.

Data access links<br>*May remain private before publication.*	*For "Initial submission" or "Revised version" documents, provide reviewer access links. For your "Final submission" document, provide a link to the deposited data.*
Files in database submission	*Provide a list of all files available in the database submission.*
Genome browser session<br>(e.g. UCSC)	*Provide a link to an anonymized genome browser session for "Initial submission" and "Revised version" documents only, to enable peer review. Write "no longer applicable" for "Final submission" documents.*

Methodology

Replicates	*Describe the experimental replicates, specifying number, type and replicate agreement.*
Sequencing depth	*Describe the sequencing depth for each experiment, providing the total number of reads, uniquely mapped reads, length of reads and whether they were paired- or single-end.*
Antibodies	*Describe the antibodies used for the ChIP-seq experiments; as applicable, provide supplier name, catalog number, clone name, and lot number.*
Peak calling parameters	*Specify the command line program and parameters used for read mapping and peak calling, including the ChIP, control and index files used.*
Data quality	*Describe the methods used to ensure data quality in full detail, including how many peaks are at FDR 5% and above 5-fold enrichment.*
Software	*Describe the software used to collect and analyze the ChIP-seq data. For custom code that has been deposited into a community repository, provide accession details.*

Flow Cytometry

Plots

Confirm that:

☒ The axis labels state the marker and fluorochrome used (e.g. CD4-FITC).

☒ The axis scales are clearly visible. Include numbers along axes only for bottom left plot of group (a 'group' is an analysis of identical markers).

☒ All plots are contour plots with outliers or pseudocolor plots.

☒ A numerical value for number of cells or percentage (with statistics) is provided.

Methodology

Sample preparation	Resuspend immune cells at 2×10^6–4×10^6cells/ml in 1ml of DPBS (BI, 02-023-1A) and stained with 0.3µl of FVS575V for 20 min at room temperature in the dark for live/dead. Cells were washed twice (400×g,5 min, 4°C) with FACS buffer (2%FBS/PBS). The cell pellet was resuspended in 90ul of FACS buffer. For Fc receptors blocking, add 4ul of anti-CD16/32 antibody and incubate for 15 min on ice. Stain the cells with CXCR5-BV650 for 10 min at RT in the dark and then incubated the cells with the other 19 kinds of surface receptor staining mix for another 30 min at RT in the dark. After incubation, cells were washed with FACS buffer twice and resuspended in 200µl FACS buffer.
Instrument	Cytek NL-CLC, Cytek Biosciences, USA
Software	FlowJo software, V10.7.1 (Tree Star, USA)
Cell population abundance	*Describe the abundance of the relevant cell populations within post-sort fractions, providing details on the purity of the samples and how it was determined.*
Gating strategy	Gating strategy is proved in the supplementary information.

☒ Tick this box to confirm that a figure exemplifying the gating strategy is provided in the Supplementary Information.

Magnetic resonance imaging

Experimental design

Design type	*Indicate task or resting state; event-related or block design.*
Design specifications	*Specify the number of blocks, trials or experimental units per session and/or subject, and specify the length of each trial or block (if trials are blocked) and interval between trials.*
Behavioral performance measures	*State number and/or type of variables recorded (e.g. correct button press, response time) and what statistics were used to establish that the subjects were performing the task as expected (e.g. mean, range, and/or standard deviation across subjects).*

Acquisition

Imaging type(s)	*Specify: functional, structural, diffusion, perfusion.*
Field strength	*Specify in Tesla*
Sequence & imaging parameters	*Specify the pulse sequence type (gradient echo, spin echo, etc.), imaging type (EPI, spiral, etc.), field of view, matrix size, slice thickness, orientation and TE/TR/flip angle.*
Area of acquisition	*State whether a whole brain scan was used OR define the area of acquisition, describing how the region was determined.*

Diffusion MRI ☐ Used ☐ Not used

Preprocessing

Preprocessing software	*Provide detail on software version and revision number and on specific parameters (model/functions, brain extraction, segmentation, smoothing kernel size, etc.).*
Normalization	*If data were normalized/standardized, describe the approach(es): specify linear or non-linear and define image types used for transformation OR indicate that data were not normalized and explain rationale for lack of normalization.*
Normalization template	*Describe the template used for normalization/transformation, specifying subject space or group standardized space (e.g. original Talairach, MNI305, ICBM152) OR indicate that the data were not normalized.*

Noise and artifact removal

Describe your procedure(s) for artifact and structured noise removal, specifying motion parameters, tissue signals and physiological signals (heart rate, respiration).

Volume censoring

Define your software and/or method and criteria for volume censoring, and state the extent of such censoring.

Statistical modeling & inference

Model type and settings

Specify type (mass univariate, multivariate, RSA, predictive, etc.) and describe essential details of the model at the first and second levels (e.g. fixed, random or mixed effects; drift or auto-correlation).

Effect(s) tested

Define precise effect in terms of the task or stimulus conditions instead of psychological concepts and indicate whether ANOVA or factorial designs were used.

Specify type of analysis: ☐ Whole brain ☐ ROI-based ☐ Both

Statistic type for inference

(See Eklund et al. 2016)

Specify voxel-wise or cluster-wise and report all relevant parameters for cluster-wise methods.

Correction

Describe the type of correction and how it is obtained for multiple comparisons (e.g. FWE, FDR, permutation or Monte Carlo).

Models & analysis

n/a	Involved in the study
☐	☐ Functional and/or effective connectivity
☐	☐ Graph analysis
☐	☐ Multivariate modeling or predictive analysis

Functional and/or effective connectivity

Report the measures of dependence used and the model details (e.g. Pearson correlation, partial correlation, mutual information).

Graph analysis

Report the dependent variable and connectivity measure, specifying weighted graph or binarized graph, subject- or group-level, and the global and/or node summaries used (e.g. clustering coefficient, efficiency, etc.).

Multivariate modeling and predictive analysis

Specify independent variables, features extraction and dimension reduction, model, training and evaluation metrics.
